# The Telomere-Telomerase System Is Detrimental to Health at High-Altitude

**DOI:** 10.3390/ijerph20031935

**Published:** 2023-01-20

**Authors:** Qadar Pasha, Manjari Rain, Sana Tasnim, Hema Kanipakam, Tashi Thinlas, Ghulam Mohammad

**Affiliations:** 1 Council of Scientific & Industrial Research-Institute of Genomics and Integrative Biology, New Delhi 110007, India; 2 Institute of Hypoxia Research, New Delhi 110067, India; 3 Academy of Scientific and Innovative Research (AcSIR), Ghaziabad 201002, Uttar Pradesh, India; 4 Department of Medicine, Sonam Norboo Memorial Hospital, Leh 194101, Ladakh, India

**Keywords:** telomere, telomerase, high-altitude, high-altitude pulmonary edema, adaptation, genetic predisposition

## Abstract

The hypobaric-hypoxia environment at high-altitude (HA, >2500 m) may influence DNA damage due to the production of reactive molecular species and high UV radiation. The telomere system, vital to chromosomal integrity and cellular viability, is prone to oxidative damages contributing to the severity of high-altitude disorders such as high-altitude pulmonary edema (HAPE). However, at the same time, it is suggested to sustain physical performance. This case-control study, comprising 210 HAPE-free (HAPE-f) sojourners, 183 HAPE-patients (HAPE-p) and 200 healthy highland natives (HLs) residing at ~3500 m, investigated telomere length, telomerase activity, and oxidative stress biomarkers. Fluidigm SNP genotyping screened 65 single nucleotide polymorphisms (SNPs) in 11 telomere-maintaining genes. Significance was attained at *p* ≤ 0.05 after adjusting for confounders and correction for multiple comparisons. Shorter telomere length, decreased telomerase activity and increased oxidative stress were observed in HAPE patients; contrarily, longer telomere length and elevated telomerase activity were observed in healthy HA natives compared to HAPE-f. Four SNPs and three haplotypes are associated with HAPE, whereas eight SNPs and nine haplotypes are associated with HA adaptation. Various gene-gene interactions and correlations between/among clinical parameters and biomarkers suggested the presence of a complex interplay underlining HAPE and HA adaptation physiology. A distinctive contribution of the telomere-telomerase system contributing to HA physiology is evident in this study. A normal telomere system may be advantageous in endurance training.

## 1. Introduction

Hypobaric hypoxia at high-altitude (HA, >2500 m) triggers oxidative stress that activates or even exaggerates several physiological pathways, disturbing the normal physiology of the body [1,2,3,4,5,6,7]. Elevated reactive oxygen species (ROS) and reactive nitrogen species are the basis of oxidative stress and may influence hypoxia-inducible factor-1 (HIF-1) mediated pulmonary leakage and also damage the sodium transport system in alveoli epithelium promoting fluid retention [8,9,10,11] to exaggerate HA-related disorders such as high-altitude pulmonary edema (HAPE [MIM: 178400]) [1,12,13]. ROS damage DNA, which is further aggravated when exposed to the high UV radiation that prevails at HA. DNA damage may occur as early as the first day of HA exposure [14]. The telomeric DNA, due to its richness in guanine, is more prone to oxidative damage than other regions of the DNA [15,16]. Since the telomere-telomerase system protects the chromosomes, shorter telomeres may decrease the viability of cells, leading to cellular dysfunction and disturbed vascular homeostasis. Telomere length has evolved as a potential candidate as a disease risk marker, with various studies reporting the association between shorter telomere length in leukocytes and age-related or stress-related diseases such as Alzheimer’s disease, diabetes, cardiovascular and pulmonary diseases, myocardial infarction, hypertension, chronic obstructive pulmonary disease (COPD) and others [17,18,19,20,21]. It clearly suggests that a normal telomere system benefits healthy people in several ways [22,23,24,25,26]. Exercise training has been demonstrated to maintain active telomerase and keep telomere length under control; thus, a longer telomere will be beneficial at altitude [23,24,25,26]. As a result, altitude training in a hypoxic environment is known to increase athletes’ exercise capacity and performance at sea level and HA [22,26,27]. However, the telomere system has yet to be investigated in detail at HA to substantiate the concept.

**Figure 1:**
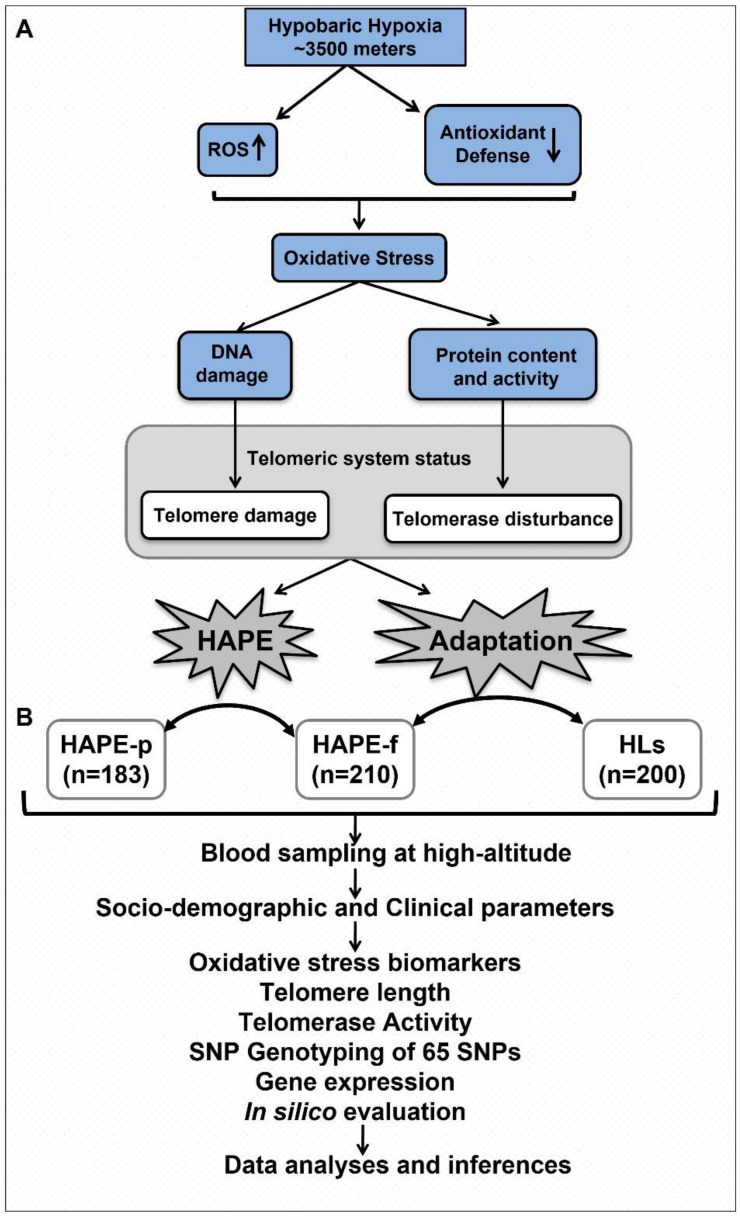
Schematic presentation of the study. (**A**) Hypothesis of the study. The telomere-telomerase system is influenced by oxidative stress under the hypobaric hypoxic environment of high-altitude. In a sequential process, they play a crucial role in HAPE pathophysiology and HA adaptation. (**B**) Study design. Study subjects were recruited, and their sampling was conducted at high-altitude (~3500 m above sea level). Socio-demographic and clinical data were collected at the time of recruitment. Plasma biomarkers namely, 8-isoPGF2α and total antioxidants, telomere length and telomerase activity were estimated, followed by SNP genotyping, gene expression and in silico analyses to evaluate the site-specific protein structure variations. All the data were analyzed using relevant statistical and bioinformatic tools and are described in the Methods section. ROS, reactive oxygen species; HAPE, high-altitude pulmonary edema; HAPE-p, HAPE patients; HAPE-f, HAPE-free healthy controls; HLs, high-land natives; SNPs, single nucleotide polymorphisms.

The telomere-telomerase system is comprised of telomeres, the shelterin complex and telomerase. It determines chromosome integrity and cell viability [28,29,30,31,32]. The shelterin complex masks the telomeric DNA from attrition-causing factors and regulates telomere extension and telomeric DNA replication [28,29,30,31]. Telomerase, a ribonucleoprotein, catalyzes telomere extension by adding six nucleotide repeats to telomeric ends [32]. Several components of the telomere-telomerase system, such as TERF1 interacting protein 2 (TINF2 [MIM: 604319]), telomerase reverse transcriptase (TERT [MIM: 187270]) and heat shock protein 90 (Hsp90/*HSP90AA1* [MIM: 140571]) regulate ROS production and oxidative stress [33,34,35,36,37]. We propose that functional impairment of shelterins and telomerase because of HA environmental stress and genetic variations may cause telomere attrition and promote HAPE and other HA disorders (Figure 1A). The present study hence comprehensively investigated the physiological and genetic significance of the telomere-telomerase system in HAPE and HA adaptation under the hypobaric hypoxic environment of HA. Physiological relevance was investigated by evaluating telomere length and telomerase activity along with the oxidative stress biomarkers, 8-iso prostaglandin F2α (8-isoPGF2α) and total antioxidant activity, and expression of the genes (Figure 1B). The genetic study included eleven genes, including shelterin genes and telomere-associated genes (Supplementary Table S2).

## 2. Materials and Methods

### 2.1. Study Subjects

The present cross-sectional case-control study was carried out in three well-characterized study groups, i.e., HAPE-free healthy controls (HAPE-f; *n* = 210), HAPE-patients (HAPE-p; *n* = 183), and high-land natives (HLs; *n* = 200). Samples were collected through the general outpatient clinic of SNM Hospital, Leh at ~3500 m after obtaining signed informed consent. The inclusion/exclusion criteria of subjects and data collection methods are detailed in the online Supplementary Methods, and the clinical characteristics of the study groups are summarized in Supplementary Table S1.

### 2.2. Ethics Approval

Ethical committees of the Council of Scientific and Industrial Research-Institute of Genomics and Integrative Biology (CSIR-IGIB), Delhi and Sonam Norboo Memorial (SNM) Hospital, Leh, Ladakh, Jammu and Kashmir approved the investigation. All procedures were performed in accordance with the Declaration of Helsinki.

### 2.3. Estimation of Relative Telomere Length

qRT-PCR was run with MESA BLUE qPCR Master Mix Plus for SYBR^®^ Assay No ROX (Eurogentec, Seraing, Belgium) on LightCycler 480 Real-Time PCR System (Roche Molecular Diagnostics, Pleasanton, CA, USA). The relative telomere length, T/S ratio, is the ratio of the telomere repeat copy number (T) to the copy number of a single copy gene (S) [38].

### 2.4. Estimation of Telomerase Activity and Oxidative Stress Markers

Telomerase activity was estimated by Quantitative Telomerase Detection Kit (Allied Biotech Inc., Ijamsville, MD, USA). The 8-isoPGF2α level was estimated using an ELISA kit (Enzo Life Sciences Inc., Farmingdale, New York, NY, USA) and total antioxidant activity spectrophotometrically (Cayman Chemical, Ann Arbor, MI, USA).

### 2.5. Genotyping of Single Nucleotide Polymorphisms

Single nucleotide polymorphisms (SNPs) were selected based on location, clinical and functional relevance, and thorough literature survey. HapMap and Indian Consortium data were referred for further selection and inspection for the presence of any tagged SNPs. The sixty-five selected SNPs included 6 *TERF1*, 5 *TERF2*, 7 *POT1*, 2 *TINF2*, 4 *ACD*, 6 *TERF2IP*, 16 *TERT*, 2 *TERC*, 9 *TEP1*, 5 *HSP90AA1* and 3 *PTGES3* (Supplementary Table S2). Fluidigm Dynamic Array Integrated Fluidic Circuit technique on a Biomark HD System (Fluidigm Corporation, South San Francisco, CA, USA) performed the genotyping.

### 2.6. Genetic Analyses

The Hardy–Weinberg equilibrium (HWE) for SNPs was checked using a χ^2^ goodness-of-fit test using EpiInfo™ ver. 6 (Center for Disease Control, Atlanta, GA, USA). Haploview 4.2 checked the Linkage-disequilibrium (LD) and the tagging efficiency of SNPs. Two or more SNPs were considered in LD with D′ ≥ 0.9 and r^2^ ≥ 0.9. Multivariate logistic regression analysis established the association of SNPs. The power of the study was calculated using the online tool OSSE-An Online Sample Size Estimator (http://osse.bii.a-star.edu.sg/calculation2.php (accessed on 14 May 2017)).

To avoid false-positive results due to ethnicity difference between HLs and HAPE-f/HAPE-p and to ascertain SNPs associating with HA adaptation, HLs were compared with two more populations, i.e., Han Chinese (CHB, Han Chinese in Beijing, China and CHS, Southern Han Chinese, China) and Japanese (JPT, Japanese in Tokyo, Japan). Genotype distribution data for CHB, CHS and JPT were procured from the 1000 Genomes Phase 3 project (www.ensembl.org (accessed on 2 June 2017)) [39]. Haplotypes were deduced from the genotypes of genes located at five chromosomes by the PHASE v. 2.1.1 program. The SNPs at the forward strand were recognized according to the contig position in the haplotypes (Supplementary Figure S1). An OR > 1 depicts risk, and OR < 1 depicts protection or adaptation.

### 2.7. Gene-Gene Interactions in HAPE and Adaptation

Gene-gene interactions for respective associated SNPs were evaluated by MDR 2.0 beta 8.4. The significant best models were selected based on higher scores of testing accuracy (TA ≤ 0.55) and cross-validation consistency (CVC = 9/10 or 10/10). A *p*-value ≤ 0.05 was considered statistically significant.

### 2.8. DNA-Protein Docking

Transcription factors (TFs) were identified using the dbSNP tool and PASTA program. The Hex docking program was used to dock TFs on the promoter region and to determine the binding affinities. PyMOL was used to visualize the docked structure. The 3D structure of TFs was retrieved from Protein Data Bank (PDB). The docking analysis was further cross-checked with the HADDOCK and CLusPRO.

### 2.9. Protein-Protein Interactions

STRING ver. 11.0 identified the protein-protein interactions of the respective 10 genes. *TERC* was excluded because it does not code a protein but is a constituent of the RNA component of telomerase.

### 2.10. Expression Analysis of Telomere Maintaining Genes

Differential gene expression was performed using qRT-PCR as detailed in the Supplementary Methods. The ΔΔCt calculated the relative change in transcript with 18S rRNA as the reference gene. Primer sequences are presented in Supplementary Table S3.

### 2.11. Statistical Analyses

Statistical tests were applied using Statistical Package for Social Sciences version 16.0 (SPSS 16.0 [IBM Inc., Armonk, NY, USA]), EpiInfo™ ver. 6 (Center for Disease Control, Atlanta, GA, USA) or Simple Interactive Statistical Analysis (SISA) online tool (http://www.quantitativeskills.com/sisa/ (accessed on 25 September 2017)). Unpaired Student’s t-tests compared the differences in baseline characteristics, demographic features, and gene expression between the two groups. Biomarkers were expressed as mean ± standard error (SE) and SISA calculated the statistical power. A general linear model (GLM) was applied to calculate age, gender and BMI-adjusted *p*-values. Multivariate logistic regression analyses of single locus and haplotype/multilocus yielded *p*-value, chi-square (χ^2^), odds ratio (OR) and 95% confidence interval (CI). The significance was attained after FDR correction (BenjaminiHochberg.xlsx calculator). Pearson’s Correlation was used to ascertain the correlations between clinical parameters and biomarkers and within biomarkers. Alleles and genotypes of SNPs associated with HAPE susceptibility and HA adaptation were correlated with clinical parameters and biomarkers. Bonferroni’s correction was applied for multiple comparisons. A *p*-value of ≤0.05 was considered statistically significant after adjusting with confounders.

## 3. Results

### 3.1. Low SaO_2_ Indicates HAPE

**Figure 2:**
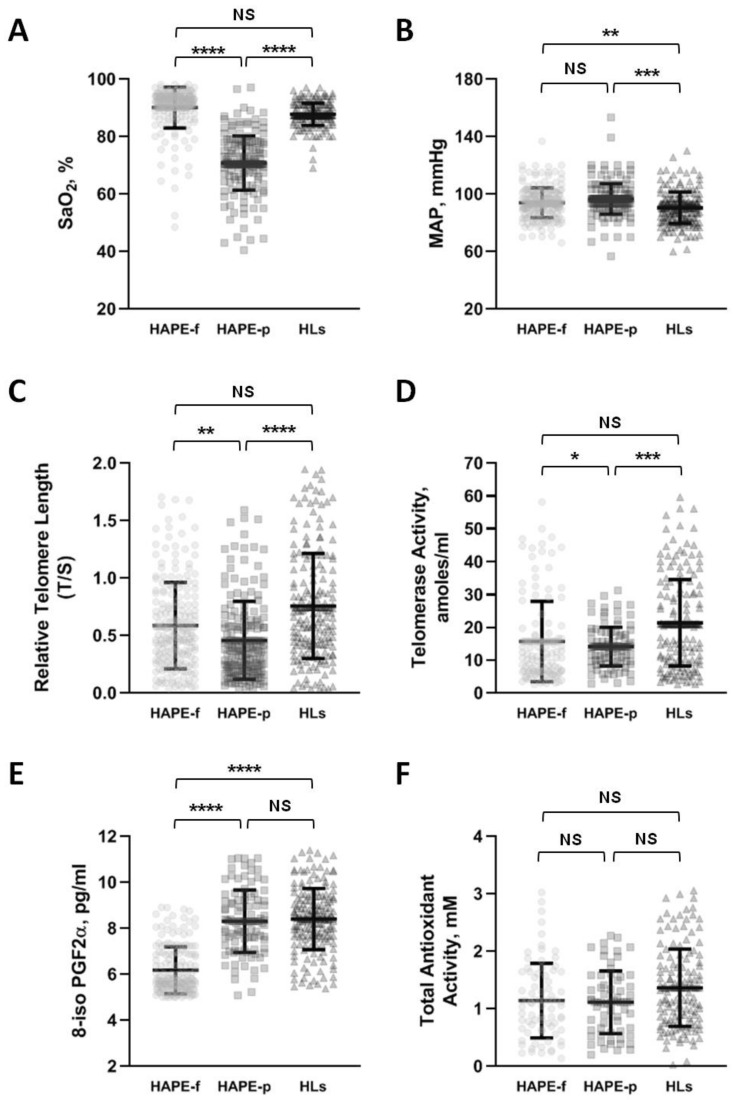
The clinical parameters and circulating biomarkers differ in the three study groups HAPE-f, HAPE-p and HLs. (**A**) Arterial oxygen saturation level is lowest in patients. (**B**) Mean arterial pressure has increased in patients whereas SaO_2_ and MAP present a reverse trend in both the control groups. (**C**) Relative Telomere Length is shorter in patients. (**D**) Telomerase Activity decreased in patients causing the shortening of the telomere length. (**E**) 8-isoprostaglandin F2α level is increased in patients reflecting stress. (**F**) Total Antioxidant Activity decreased in patients. The increased stressor and decreased antioxidant complement the two clinical parameters and the shortened telomere length and the telomerase activity in HAPE patients compared to the reverse trend in the two control groups suggesting their contribution to HAPE pathophysiology. The data are represented as mean ± SD. SPSS 16.0 was used to obtain *p*-values after adjusting with age, gender, and BMI by performing UNIANOVA. A *p*-value of ≤ 0.05 was considered statistically significant. * represents *p* ≤ 0.05, ** represents *p* ≤ 0.01, *** represents *p* ≤ 0.001 and **** represents *p* ≤ 0.0001. HAPE-f, HAPE-free healthy controls; HAPE-p, HAPE-patients; HLs, High-land natives; SaO_2_, arterial oxygen saturation; MAP, mean arterial pressure; T/S, ratio between telomere repeat copy number (T) and the copy number of single copy gene (S); 8-iso PGF2α, 8-isoprostaglandin F2α; p, *p*-value.

HAPE-p had lower SaO_2_ levels than HAPE-f. The HLs of Tibeto-Burman origin had SaO_2_ levels close to HAPE-f but higher than HAPE-p (Figure 2A). In addition, HAPE-p had increased mean arterial pressure (MAP) compared to HLs (Figure 2B) and was non-significantly higher than HAPE-f.

### 3.2. Aberrant Telomere-Telomerase System Inclined with HAPE Susceptibility

Among the three study groups, HAPE-p had the shortest relative telomere length (Figure 2C), which complies with the lower telomerase activity (Figure 2D). In contrast, longer telomere length and higher telomerase activity were observed in HLs. The relative telomere length was the longest in the HLs compared to the two sojourn groups, i.e., HAPE-p and HAPE-f (Figure 2C). However, the difference between the two healthy groups was modest. The telomerase activity was in agreement with longer telomere length in HLs compared to HAPE-p and HAPE-f (Figure 2D).

### 3.3. The Oxidative System Shows Stronger Influence

The levels of 8-isoPGF2α were elevated (Figure 2E), whereas the total antioxidant activity was slightly lower (Figure 2F) in HAPE-p compared to HAPE-f. The HLs present an interesting status of 8-isoPGF2α and total antioxidant activity. Similar to HAPE-p, the 8-isoPGF2α levels were higher in HLs than HAPE-f (Figure 2E) and were supported by the ~20% increase in the total antioxidant activity than in the two sojourn groups (Figure 2F). This suggests that HLs are under continuous stress due to an active oxidative process.

### 3.4. Genetic Association with HAPE and HA Adaptation

Sixty-five SNPs with six genes of telomere-associated shelterins, namely *TERF1*, *TERF2*, *POT1*, *TINF2*, *ACD* and *TERF2IP* (also known as *RAP1*) and five genes of telomerase system, namely *TERT*, *TERC*, *TEP1*, *HSP90AA1* and *PTGES3* were genotyped. Sixteen SNPs with <95% call rate or being non-polymorphic and six SNPs deviating from HWE in HAPE-f were excluded from the study. The remaining 43 SNPs were not consistently in LD or tagged among each other (D′ ≥ 0.90, r^2^ ≥ 0.90) in the three study groups (Supplementary Figure S2A–G). These were screened and analyzed for genotype and allele distributions and are detailed in Supplementary Tables S4–S13; however, genotypic and allelic distributions of the significant SNPs are presented in Table 1. Subtables B in Supplementary Tables S4–S12 represent comparisons between the HLs against the Han Chinese (CHB+CHS) and Japanese (JPT) populations (1000Genomes).

Three genes with respective alleles such as *TERF2IP* rs59297469T, *TERC* rs2293607G and *TEP1* rs2228036T, associated with HAPE risk (Table 1 and Supplementary Tables S8A, S10A and S11A), while *ACD* rs72556537A associated with protection against HAPE (Table 1 and Supplementary Table S7A). Eight alleles *TERF1* rs2975843T and rs10099824G, *TERF2* rs3785073G and rs153058C, *POT1* rs7794637C, *ACD* rs6979A and *TEP1* rs2184282G and rs4246977T were abundant in HLs and hence associated with HA adaptation (Table 1 and Supplementary Tables S4A, S5A, S6A, S7A and S11A). A comparison of the HLs with Han Chinese (CHB+CHS) and Japanese (JPT) populations provided mixed results, with few of the genes/SNPs having near similar distribution in the three populations (Subtables B in Supplementary Tables S4–S12).

### 3.5. Haplotypes Distribution Is Specific in HAPE and HA Adaptation

Seventy-two haplotypes with frequency > 2% were observed in eight telomere-maintaining genes across five chromosomes. Three haplotypes, namely, GAAA of *TERF1*, TCCCGACTTA and TTCCGATCTA of *TEP1*+*HSP90AA1*, were observed in HAPE protection (Table 2).

In highlanders, nine adaptive haplotypes were observed (Table 2). *TERT* was observed in the adaptive haplotype GATCGCCAG. The AAGG haplotype of *TERF1* included adaptive alleles *TERF1* rs2975843A and *TERF1* rs10099824G located at the second and fourth contig position, respectively. Five haplotypes of *ACD*+*TERF2*+*TERF2IP* were adaptive, i.e., AAAAATTCGCA, AAGTATCCGCA, AAGTATTCGCA, AAGTGTCCGCA and TGGTATCCGCA; these were comprised of adaptive alleles *ACD* rs6979A or *TERF2* rs3785073G or both at second and third position, respectively. Further, TCCCGGCTCA and TTCCGGCTCA in *TEP1*+*HSP90AA1* were adaptive, having adaptive alleles *TEP1* rs2184282C and *TEP1* rs4246977T at the seventh and eighth positions, respectively. The haplotypes could not be generated in CHB+CHS and JPT populations as the genotype data of only 49% of SNPs were available.

### 3.6. Gene-Gene Interactions

#### 3.6.1. Best Interaction Models for HAPE

**Figure 3:**
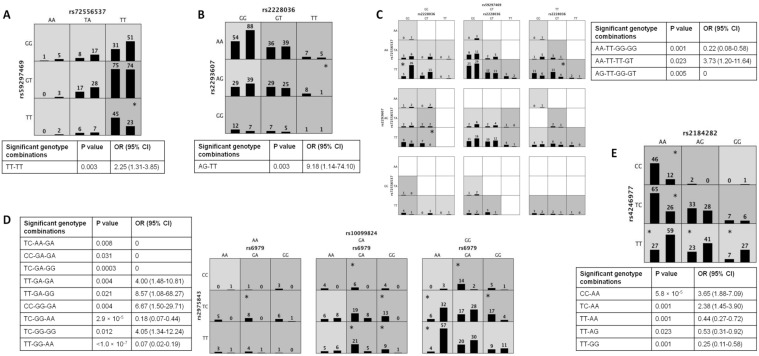
MDR generated distribution of genotype interactions in observed best models. In the Figure, sub-figures (**A**–**C**) represent a comparison of the two to four loci genotype interactions between the HAPE patients and HAPE-free controls. In sub-figures (**D**,**E**), two to three loci genotype interactions are compared between the two control groups, i.e., HAPE-f (HAPE-free healthy controls) and HLs (high-land natives). (**A**) HAPE-associating shelterin genes in a 2-locus best model. The TT-TT (*TPP1/ACD* rs72556537A/T-*RAP1/TERF2IP* rs59297469G/T) interaction was observed as risk in which the protective allele TPP1 rs72556537A was absent. (**B**) HAPE-associating telomerase genes in a 2-locus best model. A risk-predicting TT-AG (*TEP1* rs2228036G/T-*TERC* rs2293607A/G) interaction was observed in which risk allele *TEP1* rs2228036T was present as homozygous genotype *TEP1* rs2228036TT and risk allele *TERC* rs2293607G was present as heterozygous rs2293607AG genotype. (**C**) HAPE-associating genes of the telomere-telomerase system in a 4-locus best model. The TT-GT-AA-TT (*RAP1* rs59297469G/T-*TEP1* rs2228036G/T-*TERC* rs2293607A/G-*TPP1* rs72556537A/T) risk-predicting interaction consisted of risk allele *RAP1* rs59297469T as homozygous rs59297469TT genotype and risk allele *TEP1* rs2228036T as heterozygous rs2228036GT genotype. The GG-GT-AG-TT interaction consisted of risk allele *TEP1* rs2228036T as heterozygous rs2228036GT genotype and risk allele *TERC* rs2293607G as heterozygous rs2293607AG genotype. (**D**) HA adaptation-associating shelterin genes and HA adaptation-associating genes of the telomere-telomerase system in a 3-locus best model. Two interactions, GG-AA-TC (*TERF1* rs10099824G/A-*TPP1* rs6979G/A-*TERF1* rs2975843T/C) and GG-AA-TT, predicted HA adaptation consisting of adaptive allele *TERF1* rs10099824G as homozygous rs10099824GG genotype and adaptive allele *TPP1* rs6979A as homozygous rs6979AA genotype. The adaptive allele *TERF1* rs2975843T was present as heterozygous rs2975843TC genotype in GG-AA-TC and as homozygous rs2975843TT genotype in GG-AA-TT. (**E**) HA adaptation-associating telomerase genes in a 2-locus best model. Three adaptive interactions, i.e., AA-TT, AG-TT. GG-TT (*TEP1* rs2184282A/G-*TEP1* rs4246977T/C) were obtained. The adaptive allele *TEP1* rs4246977T as homozygous rs4246977TT genotype is present in all the three adaptive interactions revealing a strong association with HA adaptation. In (**A**–**C**) presentation, the dark grey cells associate with HAPE risk, and the light grey cells associate with HAPE protection; the first bar in a cell represents the number of HAPE-p and the second bar represents the number of HAPE-f. In (**D**,**E**), the dark grey cells represent interactions enriched in HAPE-f and the light grey cells associate with HA adaptation; the first bar in a cell represents the number of HAPE-f and the second bar represents the number of HLs. * marked cells represent the significant genotype interactions, which are summarized in respective tables. The genotypes of the SNPs appear in alphabetical order. Chi-square test or Fisher’s exact test (when the number of samples was ≤5) was applied to calculate *p*-value, OR (odds ratio) and 95% CI (confidence interval) using SISA online tool.

In the shelterin genes, a 2-locus MDR model, TT-TT represented by SNPs *ACD* rs72556537T/A and *TERF2IP* rs59297469G/T emerged as the best disease-predicting model (TA = 0.59, CVC = 10/10, *p* = 0.003) (Figure 3A). The telomerase SNPs, *TEP1* rs2228036G/T and *TERC* rs2293607A/G also revealed a 2-locus best model, AG-TT, for disease prediction (TA = 0.55, CVC = 10/10, *p* = 0.003) (Figure 3B). When all the four HAPE-associating SNPs were included in the analysis, a 4-locus disease-predicting model, AA-TT-TT-GT, was observed as the best (TA = 0.55, CVC = 10/10, *p* < 0.023) (Figure 3C).

#### 3.6.2. Best Interaction Models for HA Adaptation

The HA adaptation predicting MDR model among shelterin genes was represented by a 3-locus model comprising *TERF1* rs10099824G/A, *TERF1* rs2975843T/C and *ACD* rs6979G/A (TA = 0.77, CVC = 10/10, *p* < 0.0001) (Figure 3D). The telomerase genes were represented by a 2-locus best model comprising SNPs *TEP1* rs2184282A/G and rs4246977T/C (TA = 0.65, CVC = 10/10, *p* < 0.0001) (Figure 3E). Interestingly, when all the HA adaptation-associating SNPs were combined, the same 3-locus model of shelterin genes emerged as the best model with the same genotype combinations suggesting a strong interaction among the three SNPs (TA = 0.77, CVC = 10/10, *p* < 0.0001) (Figure 3D); thus, a preference for shelterin genes was visible. MDR analysis among a total of 43 SNPs did not reveal any significant interacting model for HAPE susceptibility and HA adaptation.

### 3.7. Expression Profile Differed for Telomere Maintaining Genes

**Figure 4:**
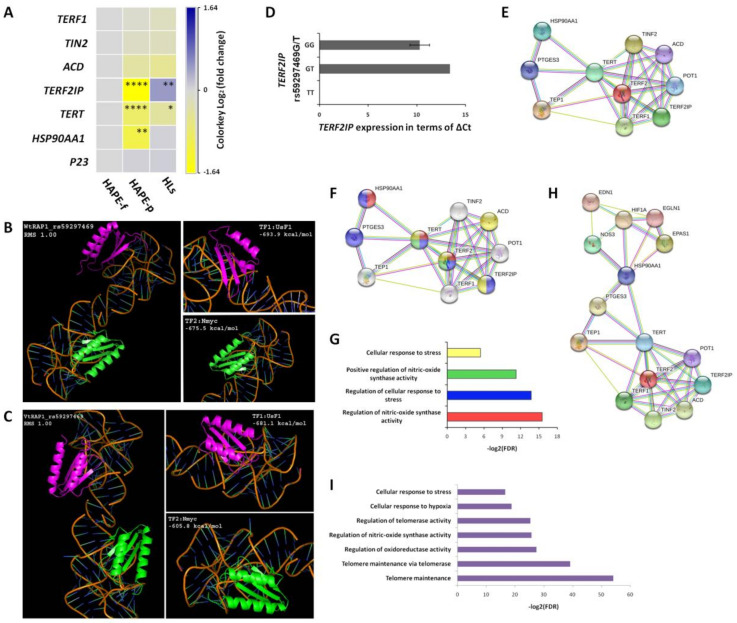
The gene expression, allele specificity with TFs and the genes/proteins interactions differentiate the physiological variations. (**A**) Heatmap of log2 fold-change value of differently expressed genes in the three study groups. *TERF2IP* was down-regulated by 3.1 folds in HAPE-p and up-regulated by 1.4 folds in HLs. *TERT* was down-regulated by 1.8 folds in HAPE-p and 1.3 folds in HLs. Further *HSP90AA1* was down-regulated 2.1 folds in HAPE-p. * represents *p* ≤ 0.05, ** represents *p* ≤ 0.01 and **** represents *p* ≤ 0.0001. (**B**) Docking image of TFs, USF1 and N-myc on *RAP1* or *TERF2IP* with wild type allele, rs59297469G (WtRAP1). (**C**) Docking image of TFs, USF1 and N-myc, on *RAP1* or *TERF2IP* with variant/risk type allele, rs59297469T (VtRAP1). Figure (**B**,**C**) differentiates the binding affinity for the same locus in the presence of risk and the protective allele that would influence the expression and function of the gene, which is depicted in the subsequent presentation. RMS, root mean square. (**D**) *RAP1* expression is significantly down-regulated with a higher ΔCt in the presence of risk allele *RAP1* rs59297469T compared to the protective allele rs59297469G. (**E**) Interactions among the telomere-maintaining proteins. (**F**) Telomere system shows stronger interactions among its proteins contributing to the regulation of other pathways such as the nitric oxide synthase (NOS) (marked red and blue) and cellular response to stress (marked green and yellow) which are depicted in subsequent presentation. (**G**) Enriched biological processes such as the nitric oxide synthase (NOS) and cellular response, which are important pathways in HAPE pathophysiology. (**H**) Interactions among the proteins of the telomere-maintaining and the hypoxia-maintaining systems in HA physiology influence several pathways. (**I**) Prominent biological processes as a result of the interaction of the proteins of the two major systems. In silico tools were applied to gain insight into the differential physiological system.

The expression profile of the 11 telomere-maintaining genes was examined in 18 HAPE-f, 10 HAPE-p and 17 HLs subjects. A significant differential expression was observed for genes *TERF2IP*, *TERT* and *HSP90AA1*. *TERF2IP* was down-regulated by 3.1 fold in HAPE-p and up-regulated by 1.4 fold in HLs (Figure 4A). The contrasting expression of *HSP90AA1* in HAPE-p and HLs identifies its importance in HA physiology. *TERT* was down-regulated by 1.8 fold in HAPE-p and 1.3 fold in HLs (Figure 4A). *HSP90AA1* was down-regulated 2.1 fold in HAPE-p but up-regulated by 1.1 fold in HLs (Figure 4A), which was in line with longer telomere length, higher telomerase activity and high levels of NO in HLs compared to HAPE-p.

### 3.8. In Silico Analysis Underlined the Association of Decreased TERF2IP Expression with Risk Alleles in HAPE

Among the genes with differential gene expression, the *TERF2IP* T allele of the promoter SNP rs59297469G/T emerged as prominent with HAPE risk. Hence, in silico tools were applied to determine the influence of this allele on *TERF2IP* (also designated as *RAP1*) expression, especially through TFs. Identifying TFs, and their binding to a gene are key steps to understanding the transcriptional regulation in the diseased condition. Here, two TFs, Upstream stimulatory factor 1 (USF1) and N-myc proto-oncogene protein (N-myc), emerged as significant. The TFs were further checked for the change in binding affinity in the presence of the wild type (Wt, rs59297469G) and variant/risk type (Vt, rs59297469T) allele on the *TERF2IP* promoter. TFs were docked on the specific promoter sites, and the Hex program identified the binding affinities, whereas PyMOL was used to visualize the docked structure. The 3D structure of USF1 (PDB ID: 1AN4) and N-myc (PDB ID: 5G1X) had a resolution of 2.90 Å and 1.72 Å, respectively. The Hex docking tool identified 650 clusters from 1000 solutions in 5.25 s with a radius cut-off of 20.0. A 250 × 250 × 250 grid was generated around the *TERF2IP*-TF complex with a prefix root mean square of −1.00. HADDOCK and CLusPRO supported the docking by Hex.

The binding affinity of WtRAP1-USF1 was −693.9 kcal/mol (Figure 4B), and of VtRAP1-USF1 was −681.1 kcal/mol (Figure 4C), while the binding affinity of WtRAP1-N-myc was −675.5 kcal/mol (Figure 4B) and of VtRAP1- N-myc was −605.5 kcal/mol (Figure 4C). Elevations in binding affinity scores of both the TFs when interacting with VtRAP1 suggested weaker intermolecular forces between TFs and *TERF2IP* as compared to the WtRAP1-TF complex, leading to shorter residence time at the binding site. Thus, in silico analysis suggested apparent transcription of *TERF2IP* in the presence of Wt rs59297469G, while down-regulation of *TERF2IP* in the presence of risk allele rs59297469T.

To further ascertain the association of risk allele rs59297469T with the down-regulation of *TERF2IP* in HAPE-p, we correlated the ΔCt values of each patient in the HAPE-p group with the rs59297469 genotypes. It was observed that the presence of risk allele rs59297469T in the GT genotype increased the ΔCt (Figure 4D) which meant *TERF2IP* expression decreased, which is in agreement with the findings of the in silico analysis. The TT genotype was not observed in the HAPE expression samples.

### 3.9. Protein-Protein Interactions

The interactions among the respective 10 shelterin proteins highlighted close bonding (Figure 4E). Biological functions related to telomere extension and its maintenance were among the top priority highlighting the relevance in the telomere-telomerase system. Regulation of NOS3 activity and stress response were inclined towards TERT, HSP90AA1 and TERF2; additionally, the stress response was also inclined towards ACD, TERF2IP and PTGES3 (Figure 4F,G).

To further identify interactions and protein networks associated with the telomere-telomerase system, we included a few of the most specific proteins of the vital HA physiology systems, namely, HIF-1-alpha (HIF1A), Egl-9 family hypoxia-inducible factor 1 (EGLN1), Endothelial PAS Domain Protein 1 (EPAS1), NOS3 and endothelin-1 (EDN1). These proteins bonded well with TERT, HSP90AA1 and PTGES3 of the telomere-telomerase system (Figure 4H). The enriched biological processes included telomere maintenance, telomerase activity, homeostasis, oxidoreductase activity, and stress response, which are essential for HA acclimatization and adaptation (Figure 4I).

### 3.10. Correlation between Biomarkers and Clinical Parameters

Correlation analyses among various clinical and biochemical parameters were mixed. The correlations were not encouraging between biomarkers and clinical parameters in HAPE-f and HAPE-p but they were in HLs. In HLs, total antioxidant activity correlated negatively with MAP (r = −0.25, *p* = 0.003) and positively with SaO_2_ (r = 0.33, *p* = 5.35 × 10^−5^), suggesting that with increased antioxidant activity, highlanders would have low MAP and heightened oxygenation, vital to the hypobaric hypoxic environment (Supplementary Table S14). Correlation analyses among the various biomarkers revealed an inverse correlation between total antioxidant activity and 8-isoPGF2α in HLs (r = −0.23, *p* = 0.005), indicating a healthy state under the given environment (Supplementary Table S15).

## 4. Discussion

The interplay between environmental, biochemical and genetic factors manipulates several physiological pathways; among these, the telomere-telomerase system is one of the crucial and sensitive systems. We expectedly observed its aberration under the hypobaric hypoxic environment of HA with shorter telomere length and lower telomerase activity influencing the health and disease cycle, HAPE susceptibility and HA adaptation. The telomere attrition may be because of increased ROS and UV radiation at HA. ROS are known to cause multiple types of DNA damage, including oxidized bases, and single-strand and double-strand breaks throughout the genome [40,41]. The consequence of such a development is chromosomal instability and decreased cellular viability, thereby increasing cellular dysfunction and diverting the body’s physiology toward disorder. The overall effect could be the dysfunction of proteins of the telomere-telomerase system contributing to disturbed body physiology. However, literature is scant on the permanent residents of HA or the sojourners. Nonetheless, it is known that shorter telomere length is associated with COPD risk, mortality in COPD and reduced pulmonary function [20,42]. Thus, shorter telomere length in HAPE patients may associate with poor pulmonary function. We could relate this phenomenon to the significantly differential SaO_2_ levels and ROS in HAPE and health. SaO_2_ drops on exposure to HA or a hypobaric environment but rises to near normal with acclimatization [12,14]. In individuals who fail to acclimatize, such as HAPE patients, the SaO_2_ levels are decreased significantly and could even lead to mortality if not treated immediately. SaO_2_ in both healthy sojourners and highlanders is generally near normal and not as low as in the HAPE patients [43,44,45]. Among the various factors influencing the body, oxygen saturation, and oxidative stress seems the most effective, other than the hypoxia-sensing pathway [13,43].

The relevance of oxidative stress biomarkers, i.e., 8-iso PGF2α and total antioxidant activity, was apparent in HAPE-p with elevated 8-isoPGF2α levels suggesting increased oxidants in these individuals, who had decreased enzyme activity and as an upshot, shorter telomeres. ROS are initially produced to stimulate a series of mechanisms allowing acclimatization and adaptation to environmental changes. However, the accumulation of ROS leads to oxidative stress and causes the oxidation of several biomolecules in a vascular system [46,47]. ROS scavenges NO and reduces its production, accompanied by a reduction in other vasodilators, thereby impairing normal vascular physiology [13,48]. We and others had previously reported decreased NO levels in HAPE patients and the association of NOS3 variants with the HAPE risk [1,7,13,49,50,51,52]. The observation also suggested that similar antioxidant activity in sojourn groups is optimal for maintaining ROS in healthy individuals but unable to normalize exaggerated ROS in HAPE, thus sustaining oxidative stress.

Genetic analyses of the telomere-telomerase system made equally credible revelations, with genes clearly associating with adaptation and maladaptation (disease). The promoter SNP rs59297469G/T of *TERF2IP*, the exonic SNPs *TERC* rs2293607A/G and *TEP1* rs2228036G/T strongly pointed to the shelterin and telomerase dysfunction and thereby the risk of HAPE. The subdued *TERF2IP* expression with the risk allele suggested its regulatory influence. We subsequently predicted allele-specific binding of TFs in the case of *TERF2IP* SNP rs59297469G/T which was corroborated by in silico analyses. The analysis identified two TFs, USF1 and N-myc, for the SNP, wherein each allele had a varied binding affinity for the TFs and thus varied transcription regulation, i.e., apparent transcription of *TERF2IP* in the presence of rs59297469G, while down-regulation in the presence of risk allele rs59297469T. This was further ascertained with the association analysis of the ΔCt values of rs59297469 genotypes for each patient that revealed elevated ΔCt in the presence of risk allele rs59297469T in the GT genotype of each patient, consequent to this decreased gene expression. Incidentally, the TT genotype was not observed in the evaluated samples to ascertain the double allelic effect. Needless to say, *TERF2IP* regulates telomere length and the expression of other genes [53,54]. Among the other relevant polymorphisms of genes, *TERC* SNP rs2293607A/G is present in the H/ACA domain of the TERC RNA, which is required for the accumulation and stability of the telomerase [55]. Whereas the *ACD/TPP1* 3’UTR SNP rs72556537T/A is associated with HAPE protection. TPP1 appears a crucial protein for acclimatization because it assists in the recruitment of telomerase onto telomeres and promotes telomerase processivity [29,31,56]. Likewise, the multiallelic involvement in protection was apparent with three protective haplotypes involving *TERF1*, *TEP1* and *HSP90AA1*, and also by gene-gene interactions.

Although the genetic association of *TERT* and *HSP90AA1* was not very prominent in HAPE susceptibility, both genes were significantly down-regulated in HAPE-p along with *TERF2IP*. Down-regulation of telomerase subunits *TERT* and *HSP90AA1* supported the decreased telomerase activity observed in HAPE-p. TERT prevents mitochondrial DNA damage, increases mitochondrial efficiency, and regulates ROS production under hypoxia; thus, its down-regulation not only suggests telomere shortening and telomerase insufficiency but also increased oxidative stress [34,35]. Down-regulated TERT may weaken protection against oxidative stress by increasing mitochondrial ROS production, reducing the protection of mitochondrial DNA from oxidative damage, and undermining mitochondrial function [34,35]. TERT also has transcriptional efficiency in influencing downstream genes that together may be crucial for HA physiology [57]. Whereas HSP90 is essential for the maintenance of telomere length, stabilization of telomerase structure, telomerase activity and regulation of NOS3 to produce NO or ROS [36,37,58,59]. Thus, HSP90AA1 can influence HA physiology by regulating the telomere-telomerase system, vascular homeostasis and oxidative stress pathways.

**Figure 5:**
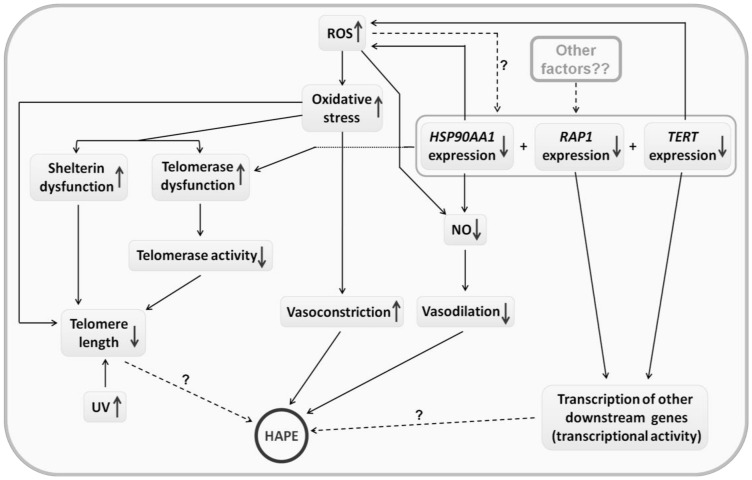
The proposed pathophysiology of HAPE. Hypobaric hypoxia is a stimulant to stress, which may in turn stimulate a normal physiological system to sustain a normal status which may not be possible in a few subjects. The same is depicted in the given graphical sketch. The expression of various proteins and genes, which are essential to regulate ROS production is dysregulated on exposure to HA. The telomere system is one of the major systems, whose genes/proteins are significantly disturbed. Down-regulation of *HSP90AA1*, *TERT* and the telomerase is observed and contributes to telomere attrition and may also enhance ROS production in a reversible process. Overall, in this milieu, the exaggerated ROS and the down-regulated candidate markers lead to the NOS3-mediated decrease in NO production and enhanced vasoconstriction leading to the disturbance of vascular homeostasis causing pulmonary hypertension, a hallmark of HAPE.

The protein-protein networking also revealed a complex interplay via association among various pathways. We observed deviations in highlanders compared to HAPE patients with longer telomere length, higher telomerase activity, 8-isoPGF2α levels, total antioxidant activity and SaO_2_ and normal MAP, suggesting an adaptive physiology [43,44]. The expression profile also suggested an altered physiology for HLs compared to HAPE. Up-regulation of *TERF2IP* and *HSP90AA1* and down-regulation of *TERT* expression were observed in HLs. Another important shelterin, *POT1*, represented by the intronic SNP rs7794637C/T, is associated with HA adaptation. It is a genetic marker for longevity, regulates telomere elongation by inhibiting telomerase, maintains telomere structure and inhibits the DNA damage response [16,60,61]. We further observed two SNPs, the intronic rs2184282A/G and the promoter rs4246977T/C from the telomerase subunit *TEP1* associating with adaptation. Interestingly, TEP1 can modulate telomere length, not via telomerase, but by alternative lengthening through associated telomere pathways [32]. Thus, the individual gene and each respective genotype emerged relevant to the telomere-telomerase system. In addition, the haplotypes and the multiallelic within genes and between genes interactions were amply visible in diseases and adaptation. Among the genes, *TERF1*, *TERF2*, *POT1*, *ACD*, *TEP1*, *TERF2IP*, *TERT* and *HSP90AA1* were at the forefront, whereas the gene-gene interactions showed a stronger association between *TERF1* and *TPP1*. Nevertheless, interactions of genes of the telomere-telomerase system with genes of other pathways such as vascular homeostasis may also exist in HA adaptation. A schematic representation of interactions of various molecules, including the telomere-telomerase system in HAPE pathophysiology, is proposed in Figure 5.

With the proposed role of shorter telomere length in HAPE pathophysiology (Figure 5), shorter telomere length among endurance athletes is observed as a stressor in their skeletal muscles [62]. On the contrary, reports have associated higher physical activity levels and exercise training with longer telomere length [23,24,25,26,27]. Our present findings would correlate with athletes that utilize altitude training to increase their exercise capacity; where being in a hypoxic environment would improve their performance at sea level or acclimatize to sports events at HA. A recent study reported that repeated and continuous exposure to HA might significantly alter oxidative stress and antioxidant capacity among HA visitors [63]. Another review highlighting the effect on telomere length and telomerase activity due to physical exercise concluded that regular aerobic activity, of moderate to vigorous intensity, preserves telomere length among athletes [27]. Thus, longer telomere length and higher telomerase activity in response to more physical activity at HA have the potential to improve/provide therapy in better adaptation at HA for mountain sports and high-altitude mountaineering.

The present study is not without limitations. First, despite the higher sample size of each cohort, replicating our findings in a much larger cohort will confirm our findings. Second, we could not investigate the HAPE patients post-recovery to obtain a veritable picture of the studied parameters and whether they were truly involved in the pathophysiology. It is, however, a difficult task to follow the patients, who are tourists, under mental trauma of becoming severely ill on vacations. They hence immediately leave the hospital upon recovery and refuse to participate in the follow-up study. A longitudinal study though would be beneficial. Third, we could not check the role of longer telomeres and telomerase activity in high-enduring sports persons; thus, replicating our findings in high-performance sports persons will confirm the role of the telomere system in athletes. Moreover, investigating multigenetic systems is desired, especially the haplotypes involving the short and long telomeres. Although we analyzed the variants (short-read telomeres) that provide an accuracy of greater than 99% (similar to long-read telomers) over a few megabases of the genome [64,65,66], investigating the long-range telomere haplotypes along with short-read telomeres in a disease state or high-endurance sports is desired. Lastly, additional experimental studies determining the cellular effect of significant genes with a human cell line would authenticate their functional consequence.

## 5. Conclusions

The present study reports the involvement of the telomere-telomerase system in HA physiology for the first time. The shorter telomere length and lower telomerase activity define HAPE; the longer telomere length and higher telomerase activity define health and adaptation at HA. Various regulatory, exonic or intronic SNPs and haplotypes of this system are crucial for HAPE susceptibility and HA adaptation. A complex interaction exists between these genes and genes of other pathways such as vascular homeostasis. A study involving more SNPs and genes from the telomere-telomerase system and different established pathways at HA might be helpful to unfold the proper framework of HAPE pathophysiology and HA adaptation. The telomere-telomerase system holds promise for future studies.

## Figures and Tables

**Table 1:** Genotype and allele distribution in HAPE-f, HAPE-p and HLs.

A. HAPE Risk/Protection										
Gene/ SNP	Genotype/ Allele	HAPE-f (*n* = 210)	HAPE-p (*n* = 183)	HLs (*n* = 200)	HAPE-p vs. HAPE-f		HLs vs. HAPE-f		HAPE-p vs. HLs	
					*p* Value	OR (95%CI)	*p* Value	OR (95%CI)	*p* Value	OR (95%CI)
*TERF2IP* rs59297469	GG	73 (34.8%)	40 (21.9%)	138 (69.0%)	Ref	-	Ref	-	Ref	-
	GT	105 (50.0%)	92 (50.2%)	57 (28.5%)	0.575	1.17 (0.68–2.01)	1.23 × 10^−4^	0.22 (0.10–0.47)	1.00 × 10^−10^	8.48 (4.43–16.20)
	TT	32 (15.2%)	51 (27.9%)	5 (2.5%)	0.037	1.98 (1.04–3.75)	8.62 × 10^−6^	0.02 (0.004–0.12)	1.03 × 10^−8^	69.64 (16.29–297.67)
	G	251 (59.8%)	172 (47.0%)	333 (83.2%)	Ref	-	Ref	-	Ref	-
	T	169 (40.2%)	194 (53.0%)	67 (16.8%)	0.082	1.33 (0.96–1.83)	2.25 × 10^−9^	0.16 (0.09–0.29)	3.19 × 10^−18^	6.81 (4.42–10.49)
*TERC* rs2293607	AA	132 (62.8%)	97 (53.0%)	32 (16.0%)	Ref	-	Ref	-	Ref	-
	AG	65 (31.0%)	66 (36.1%)	101 (50.5%)	0.099	1.51 (0.93–2.47)	1.30 × 10^−6^	9.04 (3.71–22.04)	3.16 × 10^−7^	0.18 (0.10–0.35)
	GG	13 (6.2%)	20 (10.9%)	67 (33.5%)	0.015	2.81 (1.22–6.46)	8.04 × 10^−9^	26.92 (8.79–82.43)	2.42 × 10^−9^	0.09 (0.04–0.19)
	A	329 (78.3%)	260 (71.0%)	165 (41.2%)	Ref	-	Ref	-	Ref	-
	G	91 (21.7%)	106 (29.0%)	235 (58.8%)	0.005	1.70 (1.18–2.45)	5.12 × 10^−12^	6.04 (3.62–10.06)	6.81 × 10^−12^	0.27 (0.18–0.39)
*TEP1* rs2228036	GG	134 (63.8%)	95 (52.0%)	127 (63.5%)	Ref	-	Ref	-	Ref	-
	GT	69 (32.9%)	72 (39.3%)	57 (28.5%)	0.581	1.15 (0.70–1.87)	0.267	0.67 (0.32–1.37)	0.040	1.77 (1.03–3.04)
	TT	7 (3.3%)	16 (8.7%)	16 (8.0%)	0.042	2.84 (1.04–7.77)	0.806	1.21 (0.26–5.65)	0.406	1.48 (0.59–3.73)
	G	337 (80.2%)	262 (71.6%)	311 (77.8%)	Ref	-	Ref	-	Ref	-
	T	83 (19.8%)	104 (28.4%)	89 (22.2%)	0.087	1.38 (0.95–2.01)	0.447	0.80 (0.45–1.42)	0.053	1.50 (0.99–2.27)
*ACD* rs72556537	TT	148 (70.4%)	151 (82.5%)	145 (72.5%)	Ref	-	Ref	-	Ref	-
	TA	52 (24.8%)	31 (17.0%)	48 (24.0%)	0.073	0.59 (0.34–1.05)	0.138	0.53 (0.23–1.23)	0.183	0.65 (0.34–1.23)
	AA	10 (4.8%)	1 (0.5%)	7 (3.5%)	0.038	0.11 (0.01–0.88)	0.346	1.89 (0.50–7.08)	0.008	0.05 (0.01–0.45)
	T	348 (82.9%)	333 (91.0%)	338 (84.5%)	Ref	-	Ref	-	Ref	-
	A	72 (17.1%)	33 (9.0%)	62 (15.5%)	0.003	0.48 (0.29–0.78)	0.778	0.91 (0.49–1.71)	0.002	0.43 (0.25–0.74)
**B. HA Adaptation**										
**Gene/** **SNP**	**Genotype/** **Allele**	**HAPE-f** **(*n* = 210)**	**HAPE-p** **(*n* = 183)**	**HLs** **(*n* = 200)**	**HAPE-p vs. HAPE-f**		**HLs vs. HAPE-f**		**HAPE-p vs. HLs**	
					***p* Value**	**OR (95%CI)**	***p* Value**	**OR (95%CI)**	***p* Value**	**OR (95%CI)**
*TERF1* rs2975843	TT	76 (36.2%)	61 (33.3%)	112 (56.0%)	Ref	-	Ref	-	Ref	-
	TC	97 (46.2%)	82 (44.8%)	81 (40.5%)	0.729	0.91 (0.55–1.52)	0.046	0.46 (0.22–0.99)	0.033	1.82 (1.05–3.16)
	CC	37 (17.6%)	40 (21.9%)	7 (3.5%)	0.806	1.08 (0.57–2.06)	0.007	0.15 (0.04–0.60)	2.98 × 10^−5^	9.07 (3.22–25.55)
	T	249 (59.3%)	204 (55.7%)	305 (76.2%)	Ref	-	Ref	-	Ref	-
	C	171 (40.7%)	162 (44.3%)	95 (23.8%)	0.882	1.02 (0.74–1.42)	0.003	0.46 (0.28–0.76)	1.35 × 10^−5^	2.35 (1.60–3.45)
*TERF1* rs10099824	GG	92 (43.8%)	81 (44.3%)	168 (84.0%)	Ref	-	Ref	-	Ref	-
	GA	87 (41.4%)	78 (42.6%)	28 (14.0%)	0.762	0.93 (0.56–1.53)	5.52 × 10^−6^	0.06 (0.02–0.20)	4.46 × 10^−9^	7.71 (3.90–15.27)
	AA	31 (14.8%)	24 (13.1%)	4 (2.0%)	0.256	0.67 (0.33–1.34)	4.49 × 10^−5^	0.02 (0.003–0.13)	1.77 × 10^−4^	11.08 (3.15–38.96)
	G	271 (64.5%)	240 (65.6%)	364 (91.0%)	Ref	-	Ref	-	Ref	-
	A	149 (35.5%)	126 (34.4%)	36 (9.0%)	0.285	0.83 (0.59–1.17)	7.28 × 10^−11^	0.07 (0.03–0.15)	7.05 × 10^−12^	5.96 (3.58–9.92)
*TERF2* rs3785073	GG	99 (47.2%)	76 (41.5%)	108 (54.0%)	Ref	-	Ref	-	Ref	-
	GA	83 (39.5%)	86 (47.0%)	80 (40.0%)	0.864	1.04 (0.64–1.71)	0.033	0.44 (0.20–0.94)	0.001	2.64 (1.51–4.62)
	AA	28 (13.3%)	21 (11.5%)	12 (6.0%)	0.899	0.96 (0.47–1.94)	0.018	0.16 (0.03–0.73)	0.070	2.70 (0.92–7.90)
	G	281 (66.9%)	238 (65.0%)	296 (74.0%)	Ref	-	Ref	-	Ref	-
	A	139 (33.1%)	128 (35.0%)	104 (26.0%)	0.994	1.00 (0.71–1.40)	0.002	0.43 (0.24–0.74)	0.001	1.94 (1.30–2.90)
*TERF2* rs153058	TT	129 (61.4%)	107 (58.5%)	92 (46.0%)	Ref	-	Ref	-	Ref	-
	TC	73 (34.8%)	65 (35.5%)	84 (42.0%)	0.680	1.11 (0.69–1.79)	0.178	1.61 (0.80–3.24)	0.047	0.58 (0.33–0.99)
	CC	8 (3.8%)	11 (6.0%)	24 (12.0%)	0.164	2.07 (0.74–5.79)	0.015	4.59 (1.35–15.63)	0.015	0.31 (0.12–0.80)
	T	331 (78.8%)	279 (76.2%)	268 (67.0%)	Ref	-	Ref	-	Ref	-
	C	89 (21.2%)	87 (23.8%)	132 (33.0%)	0.253	1.25 (0.85–1.82)	0.014	1.89 (1.14–3.15)	0.004	0.55 (0.37–0.83)
*POT1* rs7794637	CC	90 (42.9%)	63 (34.4%)	105 (52.5%)	Ref	-	Ref	-	Ref	-
	CT	95 (45.2%)	88 (48.1%)	82 (41.0%)	0.682	0.90 (0.54–1.49)	0.137	0.59 (0.29–1.19)	0.003	2.30 (1.32–4.00)
	TT	25 (11.9%)	32 (17.5%)	13 (6.5%)	0.297	1.43 (0.73–2.81)	0.010	0.18 (0.05–0.66)	0.003	4.22 (1.62–11.02)
	C	275 (65.5%)	214 (58.5%)	292 (73.0%)	Ref	-	Ref	-	Ref	-
	T	145 (34.5%)	152 (41.5%)	108 (27.0%)	0.480	1.13 (0.81–1.57)	0.010	0.51 (0.31–0.85)	2.62 × 10^−4^	2.04 (1.39–2.99)
*ACD* rs6979	GG	67 (31.9%)	54 (29.5%)	18 (9.0%)	Ref	-	Ref	-	Ref	-
	GA	110 (52.4%)	101 (55.2%)	74 (37.0%)	0.828	1.06 (0.64–1.74)	0.011	4.54 (1.41–14.62)	0.334	0.68 (0.31–1.49)
	AA	33 (15.7%)	28 (15.3%)	108 (54.0%)	0.982	1.01 (0.50–2.02)	3.05 × 10^−7^	41.88 (10.03–174.91)	3.74 × 10^−8^	0.07 (0.03–0.18)
	G	244 (58.1%)	209 (57.1%)	110 (27.5%)	Ref	-	Ref	-	Ref	-
	A	176 (41.9%)	157 (42.9%)	290 (72.5%)	0.844	1.03 (0.75–1.43)	3.34 × 10^−12^	7.34 (4.19–12.86)	5.62 × 10^−12^	0.26 (0.18–0.38)
*TEP1* rs2184282	AA	138 (65.7%)	134 (73.2%)	97 (48.5%)	Ref	-	Ref	-	Ref	-
	AG	58 (27.6%)	41 (22.4%)	69 (34.5%)	0.108	0.64 (0.37–1.10)	0.015	2.43 (1.18–4.97)	0.002	0.39 (0.22–0.70)
	GG	14 (6.7%)	8 (4.4%)	34 (17.0%)	0.550	0.74 (0.27–2.01)	0.004	4.60 (1.63–12.95)	2.38 × 10^−5^	0.11 (0.04–0.31)
	A	334 (79.5%)	309 (84.4%)	263 (65.8%)	Ref	-	Ref	-	Ref	-
	G	86 (20.5%)	57 (15.6%)	137 (34.2%)	0.128	0.72 (0.47–1.10)	2.95 × 10^−4^	2.52 (1.53–4.16)	7.81 × 10^−8^	0.31 (0.20–0.47)
*TEP1* rs4246977	TT	57 (27.1%)	45 (24.6%)	127 (63.5%)	Ref	-	Ref	-	Ref	-
	TC	105 (50.0%)	99 (54.1%)	60 (30.0%)	0.493	1.21 (0.70–2.11)	2.88 × 10^−4^	0.22 (0.10–0.50)	8.99 × 10^−7^	4.20 (2.37–7.44)
	CC	48 (22.9%)	39 (21.3%)	13 (6.5%)	0.525	0.81 (0.42–1.56)	4.58 × 10^−5^	0.09 (0.03–0.28)	8.50 × 10^−6^	6.76 (2.92–15.69)
	T	219 (52.1%)	189 (51.6%)	314 (78.5%)	Ref	-	Ref	-	Ref	-
	C	201 (47.9%)	177 (48.4%)	86 (21.5%)	0.538	0.90 (0.66–1.25)	2.68 × 10^−7^	0.25 (0.15–0.42)	1.35 × 10^−8^	3.08 (2.09–4.54)

SPSS 16.0 was used to obtain *p* values after adjusting with age and gender. Significance was maintained at ≤0.05 after FDR correction. HAPE-f, HAPE-free; HAPE-p, HAPE-patients; HLs, Highlanders; *n*, number of samples; OR, odds ratio; CI, confidence interval; Ref, reference.

**Table 2:** Distribution of haplotypes in the three study groups.

Gene	Haplotype	HAPE-p vs. HAPE-f			HLs vs. HAPE-f			HAPE-p vs. HLs		
		χ^2^	OR (95% CI)	*p*-Value	χ^2^	OR (95% CI)	*p*-Value	χ^2^	OR (95% CI)	*p*-Value
*TERF1*	AAGG	0.927	1.17 (0.85–1.63)	0.336	18.700	2.84 (1.77–4.56)	1.53 × 10^−5^	22.768	0.41 (0.29–0.59)	1.83 × 10^−6^
	GAAA *	5.190	0.58 (0.37–0.93)	0.023	10.145	0.22 (0.09–0.56)	0.001	4.581	2.18 (1.07–4.44)	0.032
*TPP1 + TERF2 + RAP1*	AAAAATTCGCA				6.133	4.71 (1.38–16.06)	0.013	7.470	0.05 (0.01–0.43)	0.006
	AAGTATCCGCA	1.369	0.66 (0.33–1.33)	0.242	6.893	2.42 (1.25–4.67)	0.009	12.218	0.37 (0.22–0.65)	4.73 × 10^−4^
	AAGTATTCGCA				11.734	5.49 (2.07–14.55)	0.001	12.983	0.14 (0.05–0.41)	3.14 × 10^−4^
	AAGTGTCCGCA	2.512	1.77 (0.87–3.57)	0.113	16.480	4.44 (2.16–9.12)	4.92 × 10^−5^	19.034	0.29 (0.17–0.51)	1.28 × 10^−5^
	TGGTATCCGCA	0.701	0.63 (0.21–1.87)	0.402	5.101	3.60 (1.18–10.93)	0.024			
*TERT*	GATCGCCAG	1.308	0.72 (0.41–1.26)	0.253	22.883	3.75 (2.18–6.44)	1.72 × 10^−6^	38.560	0.23 (0.15–0.37)	5.31 × 10^−10^
*TEP1 + HSP90AA1*	TCCCGACTTA *	6.003	0.23 (0.07–0.74)	0.014	0.043	0.89 (0.30–2.63)	0.835			
	TCCCGGCTCA	1.063	1.74 (0.61–4.97)	0.303	4.621	4.40 (1.14–16.98)	0.032	1.215	0.58 (0.22–1.52)	0.270
	TTCCGATCT *	8.066	0.18 (0.05–0.58)	0.005						
	TTCCGGCTCA				4.482	5.58 (1.14–27.44)	0.034	7.513	0.25 (0.09–0.68)	0.006

PHASE v2.1.1 software was used to generate haplotype combinations. SPSS 16.0 was used to obtain *p* values after adjusting with age and gender. Significance was maintained at ≤0.05 after FDR correction. * marked haplotypes are protective for HAPE and others are adaptive haplotypes. χ^2^, Chi-square; OR, Odds ratio; CI, Confidence Interval.

## Data Availability

Data are available on reasonable request. All data relevant to the study are included in the article or uploaded as supplementary information (Supplementary Methods, Supplementary Tables and Supplementary Figure).

## Supplementary Materials

The following supporting information can be downloaded at: https://www.mdpi.com/article/10.3390/ijerph20031935/s1, Figure S1: Position of each SNP and allele conversion followed in the haplotypes; Figure S2: Linkage disequilibrium plots of (A) *TERF1*, (B) *TERF2*, (C) *POT1*, (D) *ACD*, (E) *TERF2IP*, (F) *TERT*, and (G) *TEP1*; Table S1: Clinical characteristics of the three study groups; Table S2: Position of the selected SNPs on the genes; Table S3: qRT-PCR primers for gene expression profiling; Table S4: Genotype and allele distribution of four SNPs of *TERF1* [A] Comparison of genotype and allele distribution among HAPE-f, HAPE-p and HLs [B] Comparison of genotype and allele distribution in HLs with Han Chinese (CHB+CHS) population and Japanese (JPT) population; Table S5: Genotype and allele distribution of five SNPs of *TERF2* [A] Comparison of genotype and allele distribution among HAPE-f, HAPE-p and HLs [B] Comparison of genotype and allele distribution in HLs with Han Chinese (CHB+CHS) population and Japanese (JPT) population; Table S6: Genotype and allele distribution of seven SNPs of *POT1* [A] Comparison of genotype and allele distribution among HAPE-f, HAPE-p and HLs [B] Comparison of genotype and allele distribution in HLs with Han Chinese (CHB+CHS) population and Japanese (JPT) population; Table S7: Genotype and allele distribution of two SNPs of *ACD* [A] Comparison of genotype and allele distribution among HAPE-f, HAPE-p and HLs [B] Comparison of genotype and allele distribution in HLs with Han Chinese (CHB+CHS) population and Japanese (JPT) population; Table S8: Genotype and allele distribution of four SNPs of *TERF2IP* [A] Comparison of genotype and allele distribution among HAPE-f, HAPE-p and HLs [B] Comparison of genotype and allele distribution in HLs with Han Chinese (CHB+CHS) population and Japanese (JPT) population; Table S9: Genotype and allele distribution of nine SNPs of *TERT* [A] Comparison of genotype and allele distribution among HAPE-f, HAPE-p and HLs [B] Comparison of genotype and allele distribution in HLs with Han Chinese (CHB+CHS) population and Japanese (JPT) population; Table S10: Genotype and allele distribution of *TERC* SNP [A] Comparison of genotype and allele distribution among HAPE-f, HAPE-p and HLs [B] Comparison of genotype and allele distribution in HLs with Han Chinese (CHB+CHS) population and Japanese (JPT) population; Table S11: Genotype and allele distribution of nine SNPs of *TEP1* [A] Comparison of genotype and allele distribution among HAPE-f, HAPE-p and HLs [B] Comparison of genotype and allele distribution in HLs with Han Chinese (CHB+CHS) population and Japanese (JPT) population; Table S12: Genotype and allele distribution of *HSP90AA1* SNP [A] Comparison of genotype and allele distribution among HAPE-f, HAPE-p and HLs [B] Comparison of genotype and allele distribution in HLs with Han Chinese (CHB+CHS) population and Japanese (JPT) population; Table S13: Genotype and allele distribution and comparison of *PTGES3* SNPs in HAPE-f, HAPE-p and HLs; Table S14: Correlation analysis between biomarkers and clinical parameters in the three study groups; Table S15: Correlation analysis between the biomarkers in the three study groups.
