# Supplementary material for: The Telomere-Telomerase System Is Detrimental to Health at High-Altitude
Source: Int J Environ Res Public Health. 2023 Jan 20;20(3):1935. doi: 10.3390/ijerph20031935 (PMC9915065; doi:10.3390/ijerph20031935)
Supplement: Supplementary file 1 [file ijerph-20-01935-s001.zip › Supplementary Figures.pdf]

| | Gene | SNP | Observed alleles | Alleles as on forward strand |
|---|---|---|---|---|
| **Haplotype 1** *TERF1* | ***TERF1*** | rs2929589 | C/T | G/A |
| | | rs2975843 | T/C | A/G |
| | | rs2929586 | T/C | A/G |
| | | rs10099824 | G/A | G/A |
| **Haplotype 2** *POT1* | ***POT1*** | rs727505 | C/T | G/A |
| | | rs17246404 | C/T | C/T |
| | | rs7794637 | C/T | C/T |
| | | rs116916706 | C/A | C/A |
| | | rs4360236 | C/T | C/T |
| | | rs7782410 | G/A | G/A |
| | | rs10244797 | T/C | T/C |
| **Haplotype 3** *ACD+TERF2+TERF2IP* | ***ACD*** | rs72556537 | T/A | A/T |
| | | rs6979 | G/A | G/A |
| | ***TERF2*** | rs3785073 | G/A | G/A |
| | | rs8061352 | T/A | T/A |
| | | rs153058 | T/C | A/G |
| | | rs113062367 | T/C | T/C |
| | | rs4783704 | C/T | C/T |
| | ***TERF2IP*** | rs76937292 | C/T | C/T |
| | | rs59297469 | G/T | G/T |
| | | rs2233810 | C/A | C/A |
| | | rs4888444 | A/G | A/G |
| **Haplotype 4** *TERT* | ***TERT*** | rs2736122 | C/T | G/A |
| | | rs2075786 | T/C | A/G |
| | | rs4246742 | A/T | T/A |
| | | rs10069690 | C/T | C/T |
| | | rs2242652 | C/T | G/A |
| | | rs2853676 | G/A | C/T |
| | | rs2736098 | A/G | T/C |
| | | rs2853669 | C/T | G/A |
| | | rs7712562 | G/A | G/A |
| **Haplotype 5** *TEP1+HSP90AA1* | ***TEP1*** | rs2104978 | A/G | T/C |
| | | rs1713449 | C/T | C/T |
| | | rs2228041 | G/A | C/T |
| | | rs2228036 | G/T | C/A |
| | | rs1760898 | C/A | G/T |
| | | rs1760897 | T/C | A/G |
| | | rs2184282 | A/G | T/C |
| | | rs4246977 | T/C | T/C |
| | | rs75932631 | T/C | T/C |
| | ***HSP90AA1*** | rs8005905 | A/T | A/T |

Supplementary Figure S1: Position of each SNP and allele conversion followed in the haplotypes. SNPs are arranged according to contig position.

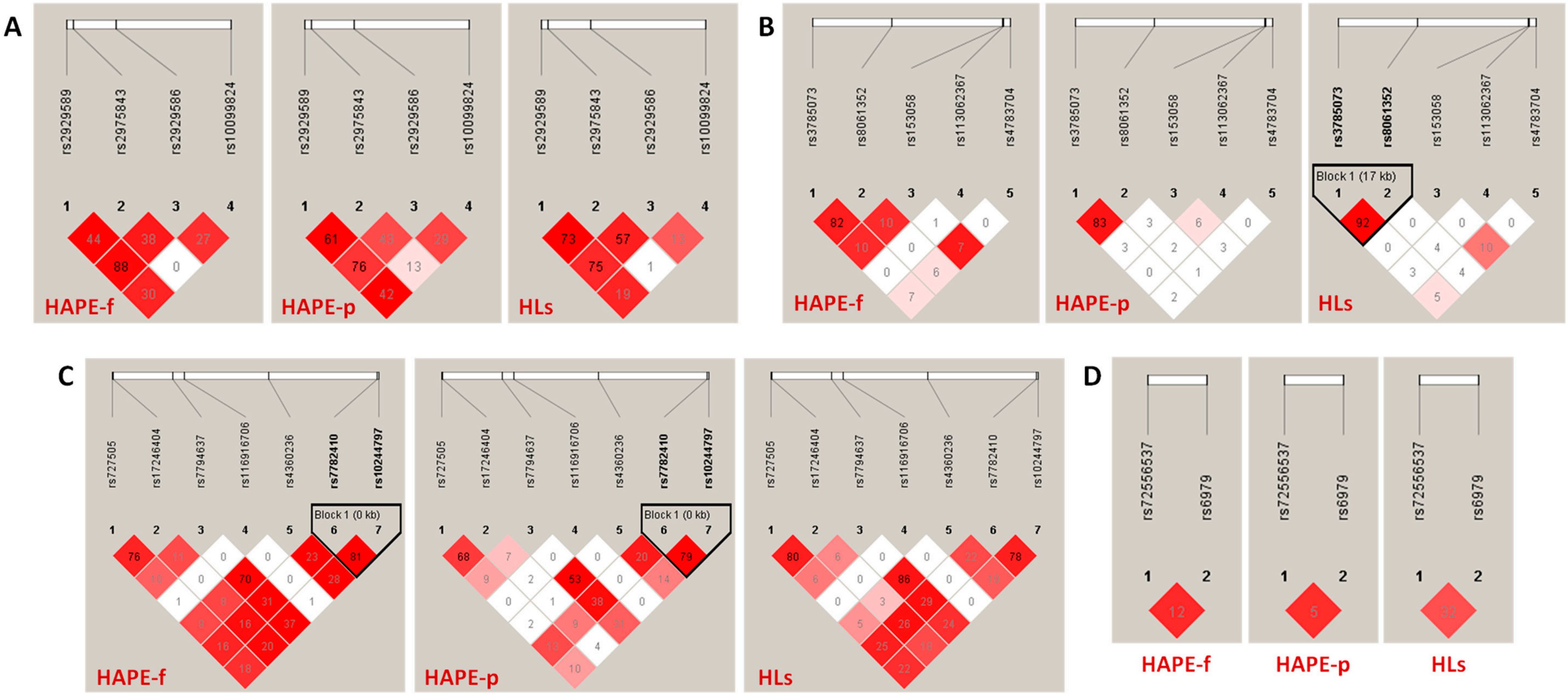
A
rs2929589
rs2975843
rs2929586
rs10099824
HAPE-f
HAPE-p
HLs
B
rs3785073
rs8061352
rs153058
rs113062367
rs4783704
Block 1 (17 kb)
HAPE-f
HAPE-p
HLs
C
rs727505
rs17246404
rs7794637
rs116916706
rs4360236
rs7782410
rs10244797
Block 1 (0 kb)
HAPE-f
HAPE-p
HLs
D
rs72556537
rs6979
HAPE-f
HAPE-p
HLs

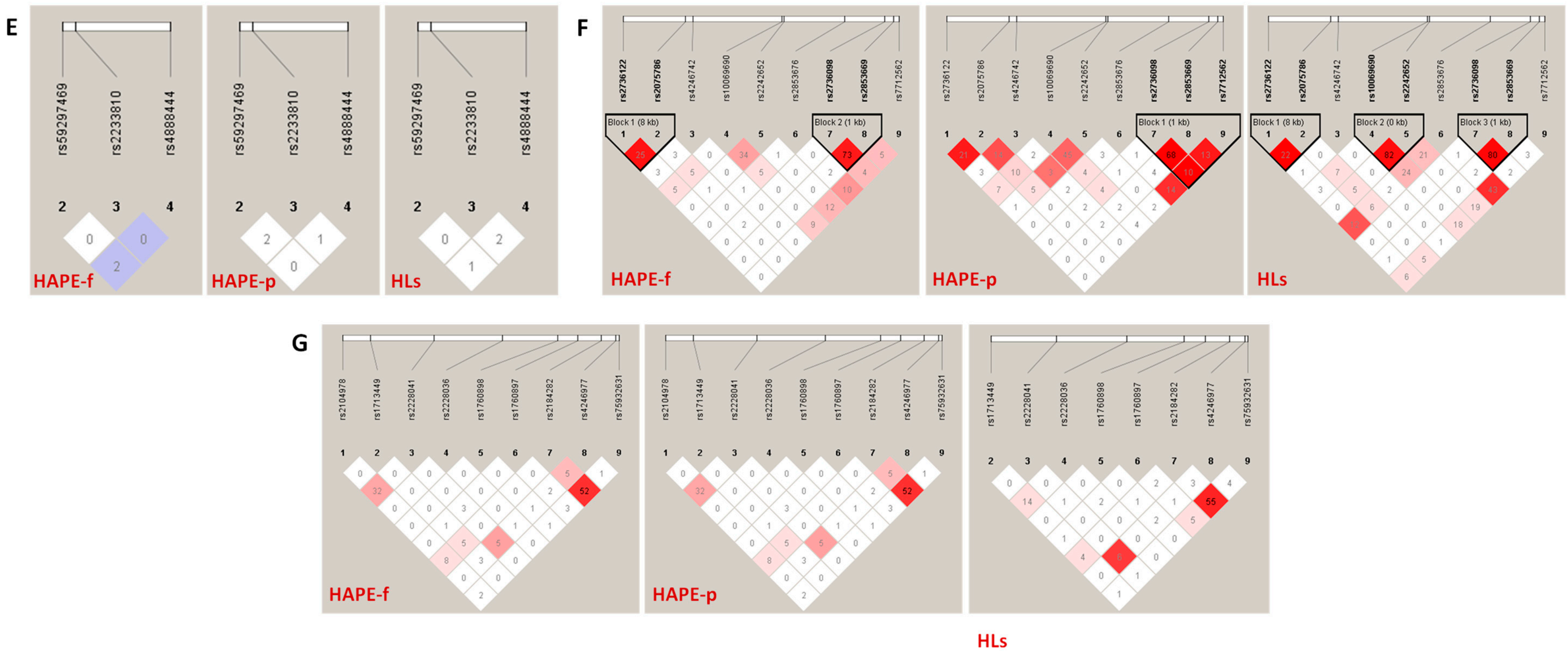


Supplementary Figure S2: Linkage disequilibrium plots of (A) *TERF1*, (B) *TERF2*, (C) *POT1*, (D) *ACD*, (E) *TERF2IP*, (F) *TERT*, and (G) *TEP1*. SNPs were arranged according to the contig position. Standard (*D'*/LOD) color scheme was kept for the LD plot. The values in the boxes represent the $r^2$ values. HAPE-f, HAPE-free; HAPE-p, HAPE-patients; HLs, highlanders; *TERF1*, telomeric repeat binding factor 1; *TERF2*, telomeric repeat binding factor 2; *POT1*, protection of telomere 1; *ACD*, ACD shelterin complex subunit and telomerase recruitment factor; *TERF2IP*, TERF2-interacting protein; *TERT*, telomerase reverse transcriptase; *TEP1*, telomerase associated protein 1.
