# Supplementary material for: The Telomere-Telomerase System Is Detrimental to Health at High-Altitude
Source: Int J Environ Res Public Health. 2023 Jan 20;20(3):1935. doi: 10.3390/ijerph20031935 (PMC9915065; doi:10.3390/ijerph20031935)
Supplement: Supplementary file 1 [file ijerph-20-01935-s001.zip › Supplementary Methods.pdf]

SUPPLEMENTARY METHODS

Study subjects

The present cross-sectional study was carried out in (1) HAPE-free healthy controls (HAPE-f; n=210) that included sojourners who did not acquire HAPE when exposed to HA; (2) HAPE-patients (HAPE-p; n=183), included sojourners who acquired HAPE on their first visit to HA, and (3) Highlanders (HLs; n=200), included the healthy natives of the HA region, residing at HA for generations. The recruited subjects were of age 15-55 years. The lowland groups, i.e., HAPE-f and HAPE-p were of Indo-Aryan ethnicity, while the highland natives, i.e., HLs were Tibeto-Burman. Subjects on any medication were excluded to obtain a true assessment of clinical and biochemical parameters. Any previous history of cardiopulmonary and other disorders was ruled out.

HAPE-f included healthy individuals who visited HA for recreational activities and/or recruited for duty at HA. They performed routine strenuous physical activities without having any HA-related disorders.

HAPE-p subjects were diagnosed on the basis of standard criteria, which included assessment of onset of typical clinical symptoms at HA. The clinical symptoms included hypoxemia, cough and dyspnea at rest, presence of pulmonary rales and cyanosis, and below normal SaO_2 level. Chest radiographic infiltrates consistent with pulmonary edema had confirmed the disorder. After recovery, HAPE-p was examined to exclude subjects with infectious diseases and any previous history of cardiopulmonary diseases. Subjects with other forms of HA-related disease were excluded from the study.

HLs were also healthy subjects residing at HA (~3,500 m) for generations. They were free from mountain disorders and any other disorders, including flu/cold.

Assessment of clinical parameters

Measurement of each clinical parameter was done in triplicates. The BP was measured in supine position after giving 5-10 min rest to the subjects, using a mercury sphygmomanometer. SaO_2 was measured by Finger-Pulse Oximeter 503 (Criticare Systems Inc, Waukesha, Wisconsin, USA). BMI

and mean arterial pressure (MAP) were calculated for each subject. Clinical characteristics of each group are summarized in Supplementary Table 1.

Sample collection

Five to seven ml of blood sample was collected in acid-citrate-dextrose anticoagulant containing falcon tube. The blood from HAPE patients was collected before the start of medication. The collected blood sample was centrifuged for 10 minutes at 1500 rpm at 4°C for plasma separation. The rest of the blood was used for genomic DNA isolation from peripheral blood leukocytes using salting out method. Plasma was aliquoted and stored at –80°C, whereas DNA was stored at –20°C.

Estimation of relative telomere length by qRT-PCR

Estimation of relative telomere length was done by calculating T/S ratio for each sample. T/S is ratio of telomere repeat copy number (T) to the copy number of single copy gene (S). Both T and S for a sample were calculated using qRT-PCR, performed for telomere repeat (Telo) and single copy gene (SCG), respectively. Genomic DNA of 10ng/ml was used in the qRT-PCR. The Telo reaction mixture included 5 µl of SYBR, 1 µl each of Telo1 and Telo2 primers, 2 µl of MQ water and 1 µl of genomic DNA. The SCG reaction mixture included 5 µl of SYBR, 0.3 µl of SCG1 primer, 0.7 µl of SCG2 primer, 3 µl of MQ water, and 1 µl of genomic DNA. The primers were diluted to a working concentration of 10 pmoles. The thermal cycle started with incubation at 95ºC for 10’, then 50 cycles of 95ºC for 15" and 60ºC for 1' followed by the melt curve cycle for both Telo and SCG PCRs.

Estimation of telomerase activity

Telomerase activity was estimated by Quantitative Telomerase Detection Kit (Allied Biotech Inc., Germantown, Maryland, USA). Protein content was estimated in each sample by bicinchoninic acid protein assay and samples were diluted to 1µg/µl by UV-treated and autoclaved Milli-Q water. The qRT-PCR was performed with 1µl of the samples as per the protocol. qRT-PCR was performed on StepOnePlus™ Real-Time PCR System (Applied Biosystems, Foster City, California, USA).

Single-locus analyses for SNP association

Supplementary Table 2 summarizes the position of the selected SNPs. The Hardy–Weinberg equilibrium (HWE) for SNPs was checked using a $\chi 2$ goodness-of-fit test using EpiInfo™ ver. 6. Linkage disequilibrium (LD) and tagging efficiency for SNPs were checked by Haploview 4.0. Two or more SNPs were considered to be in LD if D′ ≥0.9 and r2≥0.9 in HAPE-f, HAPE-p and HLs. Detailed statistical analyses to establish the association of SNPs were done by multivariate logistic regression analysis using Statistical Package for Social Sciences version 16.0 (SPSS 16.0). Multivariate logistic regression analysis yielded p-value, chi-square ($\chi 2$), odds ratio (OR) and 95% confidence interval (CI) with age and gender as confounding factors. FDR correction was applied to correct for multiple comparisons (BenjaminiHochberg.xlsx calculator). The power of the study was calculated using the online tool OSSE-An Online Sample Size Estimator (http://osse.bii.a-star.edu.sg/calculation2.php).

We performed three comparisons, including HAPE-p vs HAPE-f, HAPE-p vs HLs and HLs vs HAPE-f. An allele was said to be risk for HAPE if it had a significantly higher frequency in HAPE-p than HAPE-f and HLs and followed the trend HAPE-p>HAPE-f≥HLs. On the other hand, protective nature was confirmed when an allele was significantly over-represented in HAPE-f than HAPE-p and followed the trend HAPE-f≥HLs>HAPE-p.

To ascertain SNPs associating with HA adaptation, we compared HLs to two more populations. Genome-wide association study from our lab has revealed that HLs are genetically more close to Han Chinese (CHB, Han Chinese in Beijing, China; CHS, Southern Han Chinese, China) and Japanese (JPT, Japanese in Tokyo, Japan) populations (unpublished data). Hence, HLs genotype data was compared to CHB+CHS and JPT populations in addition to HAPE-f and HAPE-p to ascertain adaptive alleles. These two populations were included in the study to avoid positive results obtained due to the ethnicity difference between HLs and HAPE-f/HAPE-p. Genotype distribution data for CHB, CHS and JPT populations were procured from the 1000 Genome Phase 3 project (1000 Genomes Project Consortium, 2010; www.ensembl.org).

An allele was considered adaptive if it was significantly over-represented in HLs and followed the trend HLs>HAPE-f≥HAPE-p trend. Additionally, we considered that there should be differences in the

genotype and allele distribution of the identified SNP in HLs from CHB+CHS and JPT distributions to define a SNP explicitly adaptive to HLs.

Haplotype Analyses

Haplotypes were deduced from genotypes using the program PHASE v. 2.1.1, a model-based Bayesian method. The SNPs were represented as those observed at forward strand and were ordered in haplotypes according to their contig position (Supplementary Figure 1). The haplotypes whose frequency was <2% were excluded from the statistical analyses. Multivariate logistic regression analysis was done to calculate the p value, OR and 95% CI using SPSS 16.0 after adjusting with age and gender. We applied FDR correction to correct for multiple comparisons.

HAPE-p vs HAPE-f comparison was performed to determine haplotypes associating with HAPE susceptibility, while HLs vs HAPE-f comparison was performed to determine haplotypes associating with HA adaptation. Furthermore, HAPE-p vs HLs comparison was performed to support the findings of first two comparisons. We could not generate haplotypes in CHB+CHS and JPT populations as the genotype data of only 49% SNPs was available. An OR>1 depicts risk and OR<1 depicts protection or adaptation. Criteria to decipher haplotype's risk, protective or adaptive features were similar to that of interpreting SNPs'/alleles' features. Additionally, in predicting risk if a haplotype frequency follows the HAPE-p>HAPE-f>HLs trend, then it was considered as a risk haplotype when the difference between HAPE-f and HLs was ≤2%. Similarly, if a haplotype frequency follows the HLs>HAPE-f>HAPE-p trend then that haplotype was considered adaptive when the difference between HAPE-f and HAPE-p was ≤2%.

Gene-gene interactions

MDR 2.0 beta 8.4 software was used for this analysis. The significant best models obtained by this software were selected on the basis of higher scores of testing accuracy (TA) and cross-validation consistency (CVC). A p value of ≤0.05 was considered statistically significant. CVC signifies the number of times the interacting model was found significant among ten cross-validation tests.

Interaction model with a CVC of 9/10 or preferably 10/10 was considered for selecting the best model. Among the different models with 9/10 or 10/10 CVC, the best model to predict disease or adaptation was selected with the highest TA value. TA signifies the accuracy of interaction, and it should be at least 0.55 or more.

Gene expression of telomere maintaining genes

Total RNA was extracted from 2ml blood using the TRI reagent (Molecular Research Centre, Cincinnati, Ohio). The quantity and quality of the RNA were determined on a NanoDrop 2000 UV-visible spectrophotometer (Thermo Scientific, Waltham, Massachusetts, USA) and the integrity was checked on 1% agarose gel. cDNA template was synthesized from 1 μg RNA using an EZ-first strand cDNA synthesis kit (Biological Industries, Kibbutz Beit Haemek, Israel). Primers were designed by Pearl Primer software (Supplementary Table 3) and were used at a working concentration of 3 pmoles. qRT-PCR was performed in duplicate and was repeated three times for each gene and each sample. It was performed on LightCycler 480 Real Time PCR System (Roche Molecular Diagnostics, Pleasanton, California, USA) using a MESA BLUE qPCR Master Mix Plus for SYBR® Assay No ROX (Eurogentec, Liege, Belgium). The PCR cycle started with incubation at 95ºC for 5', then 40 cycles of 95ºC for 15'' and 60ºC for 1' followed by the melt curve cycle. The ΔΔCt method was used to calculate the relative change in transcript with 18S rRNA (RN18S1) as the reference gene.
