# Supplementary material for: The Telomere-Telomerase System Is Detrimental to Health at High-Altitude
Source: Int J Environ Res Public Health. 2023 Jan 20;20(3):1935. doi: 10.3390/ijerph20031935 (PMC9915065; doi:10.3390/ijerph20031935)
Supplement: Supplementary file 1 [file ijerph-20-01935-s001.zip › Supplementary Tables.pdf]

Supplementary Table S1: Clinical characteristics of the three study groups

**Clinical characteristics**	**HAPE-f (n=210)**	**HAPE-p (n=183)**	**HLs (n=200)**	**P value**		
				**HAPE-p vs HAPE-f**	**HLs vs HAPE-f**	**HAPE-p vs HLs**
Age, years	26.5±7.4	34.8±8.8	39.2±10.7	<0.001	<0.001	<0.001
Gender	M = 203 (97%)<br>F = 7 (3%)	M = 156 (85%)<br>F = 27 (15%)	M = 53 (26%)<br>F = 147 (74%)	<0.001	<0.001	<0.001
BMI, kg/m^2	19.7±2.9	25.7±5.0	21.2±3.4	<0.001	<0.001	<0.001
SBP, mmHg	120.1±13.6	122.8±13.1	119.3±14.5	0.004	0.056	0.369
DBP, mmHg	80.8±9.9	83.6±11.6	76.1±10.5	0.602	0.0004	1.81×10^{-5}
MAP, mmHg	93.9±10.4	96.7±10.6	90.5±11.0	0.117	0.002	0.001
SaO_2, %	90.1±7.1	70.8±9.4	87.7±3.9	3.60×10^{-43}	0.235	3.86×10^{-46}

The data are represented as mean±SD (standard deviation) and gender is represented as number of samples (percentage). Unpaired Student's t-test was used to compare age, gender, and BMI between any two groups using Epi Info™ ver. 6. General linear model was used to compare SBP, DBP, MAP and SaO_2 between the two groups after adjusting with age, gender, and BMI using SPSS 16.0. A P value of ≤0.05 was considered statistically significant. n, number of samples; M, male; F, female; BMI, body mass index; SBP, systolic blood pressure; DBP, diastolic blood pressure; MAP, mean arterial pressure; SaO_2, arterial oxygen saturation.

Supplementary Table S2: Position of the selected SNPs on the genes

Gene	SNPs					
	Promoter	5'UTR	3'UTR	Exonic	Intronic	Total number of SNPs
TERF1	2	-	1	1	2	6
TERF2	3	-	-	-	2	5
POT1	2	-	2	1	2	7
TINF2	2	-	-	-	-	2
ACD	1	-	1	2	-	4
TERF2IP	4	1	-	1	-	6
TERC	-	-	-	2	-	2
TERT	6	-	-	4	6	16
TEP1	2	-	-	6	1	9
HSP90AA1	1	-	2	2	-	5
PTGES3	2	-	1	-	-	3

Supplementary Table S3: qRT-PCR primers for gene expression profiling

Gene	Forward Primer	Reverse Primer
TERF1	5' GCAGAATACCTGTTTCAAAGAG 3'	5' GTTTCCCTCTCCATATTTCCT 3'
TERF2	5' ATATGAGACCTTCCAGCAG 3'	5' TTCCTTCCCTGTACTTGAG 3'
POT1	5' GATTTAAGTCACATCCATCGG 3'	5' GAAAGCTTCCAACCTTCAG 3'
TINF2	5' GAGAAAGCACTGCCTACAC 3'	5' TCCACACCATATTGTCTCCA 3'
ACD	5' TGGAGTTCAAGGAGTTTGTAG 3'	5' GTGGCTCATACTCATACTGGA 3'
TERF2IP	5' CCAGATTTGCCTGAAGAAGAG 3'	5' CTCTCATCCACCACAACCT 3'
TERT	5' GAATCAGACAGCACTTGAAGAG 3'	5' GTAGTCCATGTTCACAATCGG 3'
TERC	5' TAACTGAGAAGGGCGTAGG 3'	5' TTGCTCTAGAATGAACGGTG 3'
TEP1	5' CGGAAATTTGAGTCTTCACCT 3'	5' GCCAAGATGAGAAAGTGACC 3'
HSP90AA1	5' AGATAAACCCTGACCATTCC 3'	5' CTTCATCAATACCCAGACCA 3'
PTGES3	5' GATCCAAATGATTCCAAGCA 3'	5' TCTGAATCATCTTCCCAGTC 3'
RN18S1	5' - GCTTAATTTGACTCAACAC - 3'	5' - AGCTATCAATCTGTCAAT C - 3'

All the primers were designed to work at 60ºC annealing temperature.

Supplementary Table S4: Genotype and allele distribution of 4 SNPs of *TERF1*

[A] Comparison of genotype and allele distribution among HAPE-f, HAPE-p and HLs.

TERF1											
**SNP**	**Genetic Model**	**Genotype/ Allele**	**HAPE-f (n=210)**	**HAPE-p (n=183)**	**HLs (n=200)**	**HAPE-p vs HAPE-f**		**HLs vs HAPE-f**		**HAPE-p vs HLs**	
						**p value**	**OR (95%CI)**	**p value**	**OR (95%CI)**	**p value**	**OR (95%CI)**
rs2929589	Codominant	CC	75 (35.7%)	60 (32.8%)	15 (7.5%)	Ref	-	Ref	-	Ref	-
		CT	101 (48.1%)	83 (45.4%)	89 (44.5%)	0.975	0.99 (0.60-1.65)	0.005	4.15 (1.52-11.31)	0.002	0.31 (0.14-0.66)
		TT	34 (16.2%)	40 (21.9%)	96 (48.0%)	0.180	1.54 (0.82-2.92)	5.90×10^{-6}	10.83 (3.86-30.35)	1.48×10^{-6}	0.13 (0.06-0.30)
	Additive	C	251 (59.8%)	203 (55.5%)	119 (29.8%)	Ref	-	Ref	-	Ref	-
		T	169 (40.2%)	163 (44.5%)	281 (70.2%)	0.227	1.22 (0.88-1.68)	1.66×10^{-6}	3.27 (2.01-5.31)	1.94×10^{-7}	0.38 (0.26-0.54)
	Dominant	CC	75 (35.7%)	60 (32.8%)	15 (7.5%)	Ref	-	Ref	-	Ref	-
		CT+TT	135 (64.3%)	123 (67.2%)	185 (92.5%)	0.608	1.13 (0.70-1.82)	2.13×10^{-4}	6.69 (2.45-18.30)	4.22×10^{-5}	0.22 (0.11-0.45)
	Recessive	CC+CT	176 (83.8%)	143 (78.1%)	104 (52.0%)	Ref	-	Ref	-	Ref	-
		TT	34 (16.2%)	40 (21.9%)	96 (48.0%)	0.125	1.57 (0.88-2.80)	9.05×10^{-5}	4.19 (2.04-8.58)	3.74×10^{-5}	0.32 (0.18-0.55)
	Overdominant	CC+TT	109 (51.9%)	100 (54.6%)	111 (55.5%)	Ref	-	Ref	-	Ref	-
		CT	101 (48.1%)	83 (45.4%)	89 (44.5%)	0.477	0.85 (0.54-1.33)	0.654	0.86 (0.45-1.65)	0.551	1.17 (0.70-1.94)
rs2975843	Codominant	TT	76 (36.2%)	61 (33.3%)	112 (56.0%)	Ref	-	Ref	-	Ref	-
		TC	97 (46.2%)	82 (44.8%)	81 (40.5%)	0.729	0.91 (0.55-1.52)	0.046	0.46 (0.22-0.99)	0.033	1.82 (1.05-3.16)
		CC	37 (17.6%)	40 (21.9%)	7 (3.5%)	0.806	1.08 (0.57-2.06)	0.007	0.15 (0.04-0.60)	2.98×10^{-5}	9.07 (3.22-25.55)
	Additive	T	249 (59.3%)	204 (55.7%)	305 (76.2%)	Ref	-	Ref	-	Ref	-
		C	171 (40.7%)	162 (44.3%)	95 (23.8%)	0.882	1.02 (0.74-1.42)	0.003	0.46 (0.28-0.76)	1.35×10^{-5}	2.35 (1.60-3.45)
	Dominant	TT	76 (36.2%)	61 (33.3%)	112 (56.0%)	Ref	-	Ref	-	Ref	-
		TC+CC	134 (63.8%)	122 (66.7%)	88 (44.0%)	0.895	0.97 (0.60-1.56)	0.010	0.42 (0.21-0.81)	0.001	2.37 (1.42-3.96)
	Recessive	TT+TC	173 (82.4%)	143 (78.1%)	193 (96.5%)	Ref	-	Ref	-	Ref	-
		CC	37 (17.6%)	40 (21.9%)	7 (3.5%)	0.677	1.13 (0.64-1.98)	0.022	0.22 (0.06-0.80)	1.94×10^{-4}	6.29 (2.39-16.55)
	Overdominant	TT+CC	113 (53.8%)	101 (55.2%)	119 (59.5%)	Ref	-	Ref	-	Ref	-
		TC	97 (46.2%)	82 (44.8%)	81 (40.5%)	0.647	0.90 (0.57-1.42)	0.285	0.70 (0.36-1.35)	0.513	1.19 (0.71-1.97)

SNP	Genetic Model	Genotype/ Allele	HAPE-f (n=210)	HAPE-p (n=183)	HLs (n=200)	HAPE-p vs HAPE-f		HLs vs HAPE-f		HAPE-p vs HLs	
						p value	OR (95%CI)	p value	OR (95%CI)	p value	OR (95%CI)
rs2929586	Codominant	TT	77 (36.7%)	60 (32.8%)	18 (9.0%)	Ref	-	Ref	-	Ref	-
		TC	101 (48.1%)	83 (45.3%)	89 (44.5%)	0.933	0.98 (0.59-1.62)	0.054	2.42 (0.99-5.92)	0.014	0.40 (0.19-0.83)
		CC	32 (15.2%)	40 (21.9%)	93 (46.5%)	0.123	1.66 (0.87-3.17)	2.95×10^{-5}	8.38 (3.09-22.72)	1.22×10^{-5}	0.18 (0.08-0.39)
	Additive	T	255 (60.7%)	203 (55.5%)	125 (31.2%)	Ref	-	Ref	-	Ref	-
		C	165 (39.3%)	163 (44.5%)	275 (68.8%)	0.169	1.25 (0.91-1.73)	6.29×10^{-6}	3.00 (1.86-4.84)	2.58×10^{-6}	0.42 (0.29-0.60)
	Dominant	TT	77 (36.7%)	60 (32.8%)	18 (9.0%)	Ref	-	Ref	-	Ref	-
		TC+CC	133 (63.3%)	123 (67.2%)	182 (91.0%)	0.583	1.14 (0.71-1.83)	0.002	3.94 (1.65-9.41)	3.84×10^{-4}	0.29 (0.15-0.57)
	Recessive	TT+TC	178 (84.8%)	143 (78.1%)	107 (53.5%)	Ref	-	Ref	-	Ref	-
		CC	32 (15.2%)	40 (21.9%)	93 (46.5%)	0.071	1.71 (0.95-3.08)	4.80×10^{-5}	4.59 (2.20-9.56)	1.11E-04	0.34 (0.20-0.59)
	Overdominant	TT+CC	109 (51.9%)	100 (54.7%)	111 (55.5%)	Ref	-	Ref	-	Ref	-
		TC	101 (48.1%)	83 (45.3%)	89 (44.5%)	0.386	0.82 (0.52-1.29)	0.302	0.71 (0.37-1.36)	0.454	1.21 (0.73-2.02)
rs10099824	Codominant	GG	92 (43.8%)	81 (44.3%)	168 (84.0%)	Ref	-	Ref	-	Ref	-
		GA	87 (41.4%)	78 (42.6%)	28 (14.0%)	0.762	0.93 (0.56-1.53)	5.52×10^{-6}	0.06 (0.02-0.20)	4.46×10^{-9}	7.71 (3.90-15.27)
		AA	31 (14.8%)	24 (13.1%)	4 (2.0%)	0.256	0.67 (0.33-1.34)	4.49×10^{-5}	0.02 (0.003-0.13)	1.77×10^{-4}	11.08 (3.15-38.96)
	Additive	G	271 (64.5%)	240 (65.6%)	364 (91.0%)	Ref	-	Ref	-	Ref	-
		A	149 (35.5%)	126 (34.4%)	36 (9.0%)	0.285	0.83 (0.59-1.17)	7.28×10^{-11}	0.07 (0.03-0.15)	7.05×10^{-12}	5.96 (3.58-9.92)
	Dominant	GG	92 (43.8%)	81 (44.3%)	168 (84.0%)	Ref	-	Ref	-	Ref	-
		GA+AA	118 (56.2%)	102 (55.7%)	32 (16.0%)	0.527	0.86 (0.55-1.36)	2.37×10^{-8}	0.06 (0.02-0.16)	6.54×10^{-11}	8.04 (4.30-15.02)
	Recessive	GG+GA	179 (85.2%)	159 (86.9%)	196 (98.0%)	Ref	-	Ref	-	Ref	-
		AA	31 (14.8%)	24 (13.1%)	4 (2.0%)	0.230	0.66 (0.33-1.30)	1.95×10^{-4}	0.03 (0.01-0.20)	0.003	6.70 (1.89-23.70)
	Overdominant	GG+AA	123 (58.6%)	105 (57.4%)	172 (86.0%)	Ref	-	Ref	-	Ref	-
		GA	87 (41.4%)	78 (42.6%)	28 (14.0%)	0.851	1.04 (0.66-1.65)	3.20×10^{-5}	0.13 (0.05-0.34)	9.06×10^{-8}	5.88 (3.07-11.25)

SPSS 16.0 was used to obtain p values after adjusting with age and gender. Significance was maintained at ≤0.05 after FDR correction. *TERF1*, telomere repeat binding factor 1; HAPE-f, HAPE-free; HAPE-p, HAPE-patients; HLs, Highlanders; n, number of samples; OR, odds ratio; CI, confidence interval; Ref, reference.

[B] Comparison of genotype and allele distribution in HLs with Han Chinese (CHB+CHS) population and Japanese (JPT) population.

TERF1								
**SNP**	**Genotype/ Allele**	**CHB+CHS (Ref, n=204)**	**JPT (Ref, n=104)**	**HLs (n=200)**	**HLs vs CHB+CHS**		**HLs vs JPT**	
					**p value**	**OR (95%CI)**	**p value**	**OR (95%CI)**
rs2929589	CC	13 (6.3%)	5 (4.8%)	15 (7.5%)	Ref	-	Ref	-
	CT	86 (41.3%)	33 (31.7%)	89 (44.5%)	0.790	0.90 (0.40-2.00)	1.000	0.90 (0.30-2.67)
	TT	109 (52.4%)	66 (63.5%)	96 (48.0%)	0.503	0.76 (0.35-1.69)	0.227	0.48 (0.17-1.40)
	C	112 (26.9%)	43 (20.7%)	119 (29.8%)	Ref	-	Ref	-
	T	304 (73.1%)	165 (79.3%)	281 (70.2%)	0.370	0.87 (0.64-1.18)	0.016	0.62 (0.41-0.92)
rs2975843	TT	141 (67.8%)	82 (78.8%)	112 (56.0%)	Ref	-	Ref	-
	TC	64 (30.8%)	22 (21.2%)	81 (40.5%)	0.026	1.59 (1.06-2.40)	3.24×10^{-4}	2.70 (1.55-4.68)
	CC	3 (1.4%)	0 (0.0%)	7 (3.5%)	0.193	2.94 (0.74-11.62)	*	-
	T	346 (83.2%)	186 (89.4%)	305 (76.3%)	Ref	-	Ref	-
	C	70 (16.8%)	22 (10.6%)	95 (23.8%)	0.014	1.54 (1.09-2.17)	9.30×10^{-5}	2.63 (1.60-4.33)
rs2929586	TT	14 (6.7%)	7 (6.7%)	18 (9.0%)	Ref	-	Ref	-
	TC	90 (43.3%)	39 (37.5%)	89 (44.5%)	0.496	0.77 (0.36-1.64)	0.806	0.89 (0.34-2.30)
	CC	104 (50.0%)	58 (55.8%)	93 (46.5%)	0.342	0.70 (0.33-1.48)	0.318	0.62 (0.25-1.59)
	T	118 (28.4%)	53 (25.5%)	125 (31.3%)	Ref	-	Ref	-
	C	298 (71.6%)	155 (74.5%)	275 (68.8%)	0.368	0.87 (0.65-1.18)	0.138	0.75 (0.52-1.10)
rs10099824	GG	151 (72.6%)	77 (74.0%)	168 (84.0%)	Ref	-	Ref	-
	GA	52 (25.0%)	25 (24.0%)	28 (14.0%)	0.005	0.48 (0.29-0.81)	0.029	0.51 (0.28-0.94)
	AA	5 (2.4%)	2 (1.9%)	4 (2.0%)	0.741	0.72 (0.19-2.73)	1.000	0.92 (0.16-5.11)
	G	354 (85.1%)	179 (86.1%)	364 (91.0%)	Ref	-	Ref	-
	A	62 (14.9%)	29 (13.9%)	36 (9.0%)	0.010	0.56 (0.37-0.87)	0.061	0.61 (0.36-1.03)

SISA online tool was used to obtain p values. Significance was maintained at ≤0.05 after FDR correction. *Analysis could not be performed because the representatives of a genotype were absent in a study group. *TERF1*, telomere repeat binding factor 1; CHB+CHS, Han Chinese population comprising of Han Chinese in Beijing (CHB) and Southern Han Chinese (CHS); JPT, Japanese population; HLs, highlanders; Ref, reference; n, number of samples; OR, odds ratio; CI, confidence interval.

Supplementary Table S5: Genotype and allele distribution of 5 SNPs of *TERF2*

[A] Comparison of genotype and allele distribution among HAPE-f, HAPE-p and HLs.

TERF2											
**SNP**	**Genetic Model**	**Genotype/ Allele**	**HAPE-f (n=210)**	**HAPE-p (n=183)**	**HLs (n=200)**	**HAPE-p vs HAPE-f**		**HLs vs HAPE-f**		**HAPE-p vs HLs**	
						**p value**	**OR (95%CI)**	**p value**	**OR (95%CI)**	**p value**	**OR (95%CI)**
rs3785073	Codominant	GG	99 (47.2%)	76 (41.5%)	108 (54.0%)	Ref	-	Ref	-	Ref	-
		GA	83 (39.5%)	86 (47.0%)	80 (40.0%)	0.864	1.04 (0.64-1.71)	0.033	0.44 (0.20-0.94)	0.001	2.64 (1.51-4.62)
		AA	28 (13.3%)	21 (11.5%)	12 (6.0%)	0.899	0.96 (0.47-1.94)	0.018	0.16 (0.03-0.73)	0.070	2.70 (0.92-7.90)
	Additive	G	281 (66.9%)	238 (65.0%)	296 (74.0%)	Ref	-	Ref	-	Ref	-
		A	139 (33.1%)	128 (35.0%)	104 (26.0%)	0.994	1.00 (0.71-1.40)	0.002	0.43 (0.24-0.74)	0.001	1.94 (1.30-2.90)
	Dominant	GG	99 (47.2%)	76 (41.5%)	108 (54.0%)	Ref	-	Ref	-	Ref	-
		GA+AA	111 (52.8%)	107 (58.5%)	92 (46.0%)	0.874	1.04 (0.66-1.63)	0.009	0.40 (0.20-0.79)	3.31×10^{-4}	2.67 (1.56-4.55)
	Recessive	GG+GA	182 (86.7%)	162 (88.5%)	188 (94.0%)	Ref	-	Ref	-	Ref	-
		AA	28 (13.3%)	21 (11.5%)	12 (6.0%)	0.823	0.92 (0.47-1.84)	0.041	0.21 (0.05-0.94)	0.298	1.66 (0.64-4.32)
	Overdominant	GG+AA	127 (60.5%)	97 (53.0%)	120 (60.0%)	Ref	-	Ref	-	Ref	-
		GA	83 (39.5%)	86 (47.0%)	80 (40.0%)	0.756	1.08 (0.68-1.70)	0.124	0.58 (0.29-1.16)	0.002	2.35 (1.36-4.05)
rs8061352	Codominant	TT	101 (48.1%)	70 (38.2%)	107 (53.5%)	Ref	-	Ref	-	Ref	-
		TA	82 (39.0%)	94 (51.4%)	80 (40.0%)	0.021	1.78 (1.09-2.89)	0.501	0.78 (0.38-1.60)	1.67×10^{-4}	2.92 (1.67-5.11)
		AA	27 (12.9%)	19 (10.4%)	13 (6.5%)	0.693	1.16 (0.56-2.39)	0.116	0.37 (0.11-1.28)	0.090	2.47 (0.87-7.04)
	Additive	T	284 (67.6%)	234 (63.9%)	294 (73.5%)	Ref	-	Ref	-	Ref	-
		A	136 (32.4%)	132 (36.1%)	106 (26.5%)	0.155	1.28 (0.91-1.79)	0.116	0.65 (0.38-1.11)	0.001	1.97 (1.32-2.93)
	Dominant	TT	101 (48.1%)	70 (38.2%)	107 (53.5%)	Ref	-	Ref	-	Ref	-
		TA+AA	109 (51.9%)	113 (61.8%)	93 (46.5%)	0.036	1.63 (1.03-2.59)	0.248	0.68 (0.35-1.31)	1.12×10^{-4}	2.87 (1.68-4.91)
	Recessive	TT+TA	183 (87.1%)	164 (89.6%)	187 (93.5%)	Ref	-	Ref	-	Ref	-
		AA	27 (12.9%)	19 (10.4%)	13 (6.5%)	0.769	0.90 (0.44-1.82)	0.155	0.38 (0.10-1.43)	0.440	1.45 (0.56-3.76)

SNP	Genetic Model	Genotype/ Allele	HAPE-f (n=210)	HAPE-p (n=183)	HLs (n=200)	HAPE-p vs HAPE-f		HLs vs HAPE-f		HAPE-p vs HLs	
						p value	OR (95%CI)	p value	OR (95%CI)	p value	OR (95%CI)
rs153058	Codominant	TT	129 (61.4%)	107 (58.5%)	92 (46.0%)	Ref	-	Ref	-	Ref	-
		TC	73 (34.8%)	65 (35.5%)	84 (42.0%)	0.680	1.11 (0.69-1.79)	0.178	1.61 (0.80-3.24)	0.047	0.58 (0.33-0.99)
		CC	8 (3.8%)	11 (6.0%)	24 (12.0%)	0.164	2.07 (0.74-5.79)	0.015	4.59 (1.35-15.63)	0.015	0.31 (0.12-0.80)
	Additive	T	331 (78.8%)	279 (76.2%)	268 (67.0%)	Ref	-	Ref	-	Ref	-
		C	89 (21.2%)	87 (23.8%)	132 (33.0%)	0.253	1.25 (0.85-1.82)	0.014	1.89 (1.14-3.15)	0.004	0.55 (0.37-0.83)
	Dominant	TT	129 (61.4%)	107 (58.5%)	92 (46.0%)	Ref	-	Ref	-	Ref	-
		TC+CC	81 (38.6%)	76 (41.5%)	108 (54.0%)	0.440	1.20 (0.76-1.89)	0.054	1.91 (0.99-3.67)	0.012	0.52 (0.31-0.86)
	Recessive	TT+TC	202 (96.2%)	172 (94.0%)	176 (88.0%)	Ref	-	Ref	-	Ref	-
		CC	8 (3.8%)	11 (6.0%)	24 (12.0%)	0.179	2.00 (0.73-5.47)	0.028	3.86 (1.16-12.82)	0.038	0.38 (0.15-0.95)
	Overdominant	TT+CC	137 (65.2%)	118 (64.5%)	116 (58.0%)	Ref	-	Ref	-	Ref	-
		TC	73 (34.8%)	65 (35.5%)	84 (42.0%)	0.870	1.04 (0.65-1.67)	0.443	1.30 (0.67-2.51)	0.160	0.69 (0.41-1.16)
rs113062367	Codominant	TT	141 (67.1%)	128 (69.9%)	146 (73.0%)	Ref	-	Ref	-	Ref	-
		TC	63 (30.0%)	51 (27.9%)	49 (24.5%)	0.699	1.11 (0.66-1.85)	0.288	0.64 (0.29-1.45)	0.921	1.03 (0.58-1.83)
		CC	6 (2.9%)	4 (2.2%)	5 (2.5%)	0.051	0.17 (0.03-1.01)	0.052	0.14 (0.02-1.02)	0.710	0.73 (0.14-3.86)
	Additive	T	345 (82.1%)	307 (83.9%)	341 (85.2%)	Ref	-	Ref	-	Ref	-
		C	75 (17.9%)	59 (16.1%)	59 (14.8%)	0.493	0.86 (0.56-1.33)	0.064	0.54 (0.28-1.04)	0.868	0.96 (0.59-1.57)
	Dominant	TT	141 (67.1%)	128 (69.9%)	146 (73.0%)	Ref	-	Ref	-	Ref	-
		TC+CC	69 (32.9%)	55 (30.1%)	54 (27.0%)	0.871	0.96 (0.59-1.57)	0.159	0.59 (0.28-1.23)	0.977	0.99 (0.57-1.73)
	Recessive	TT+TC	204 (97.1%)	179 (97.8%)	195 (97.5%)	Ref	-	Ref	-	Ref	-
		CC	6 (2.9%)	4 (2.2%)	5 (2.5%)	0.043	0.17 (0.03-0.95)	0.029	0.10 (0.01-0.79)	0.608	0.63 (0.11-3.66)
	Overdominant	TT+CC	147 (70.0%)	132 (72.1%)	151 (75.5%)	Ref	-	Ref	-	Ref	-
		TC	63 (30.0%)	51 (27.9%)	49 (24.5%)	0.644	1.12 (0.68-1.85)	0.458	0.76 (0.36-1.58)	0.895	1.04 (0.59-1.83)

SNP	Genetic Model	Genotype/ Allele	HAPE-f (n=210)	HAPE-p (n=183)	HLs (n=200)	HAPE-p vs HAPE-f		HLs vs HAPE-f		HAPE-p vs HLs	
						p value	OR (95%CI)	p value	OR (95%CI)	p value	OR (95%CI)
rs4783704	Codominant	CC	121 (57.6%)	119 (65.0%)	103 (51.5%)	Ref	-	Ref	-	Ref	-
		CT	72 (34.3%)	52 (28.4%)	81 (40.5%)	0.344	0.79 (0.48-1.29)	0.452	1.31 (0.65-2.65)	0.143	0.66 (0.38-1.15)
		TT	17 (8.1%)	12 (6.6%)	16 (8.0%)	0.956	0.98 (0.40-2.38)	0.142	2.26 (0.76-6.68)	0.025	0.35 (0.14-0.88)
	Additive	C	314 (74.8%)	290 (79.2%)	287 (71.8%)	Ref	-	Ref	-	Ref	-
		T	106 (25.2%)	76 (20.8%)	113 (28.2%)	0.533	0.89 (0.61-1.30)	0.108	1.53 (0.91-2.56)	0.009	0.58 (0.38-0.87)
	Dominant	CC	121 (57.6%)	119 (65.0%)	103 (51.5%)	Ref	-	Ref	-	Ref	-
		CT+TT	89 (42.4%)	64 (35.0%)	97 (48.5%)	0.400	0.82 (0.52-1.30)	0.232	1.49 (0.77-2.88)	0.037	0.58 (0.35-0.97)
	Recessive	CC+CT	193 (91.9%)	171 (93.4%)	184 (92.0%)	Ref	-	Ref	-	Ref	-
		TT	17 (8.1%)	12 (6.6%)	16 (8.0%)	0.879	1.07 (0.45-2.56)	0.148	2.25 (0.75-6.75)	0.042	0.40 (0.16-0.97)
	Overdominant	CC+TT	138 (65.7%)	131 (71.6%)	119 (59.5%)	Ref	-	Ref	-	Ref	-
		CT	72 (34.3%)	52 (28.4%)	81 (40.5%)	0.335	0.79 (0.48-1.28)	0.694	1.15 (0.58-2.23)	0.321	0.76 (0.45-1.30)

SPSS 16.0 was used to obtain p values after adjusting with age and gender. Significance was maintained at ≤0.05 after FDR correction. *TERF2*, telomere repeat binding factor 2; HAPE-f, HAPE-free; HAPE-p, HAPE-patients; HLs, Highlanders; n, number of samples; OR, odds ratio; CI, confidence interval; Ref, reference.

[B] Comparison of genotype and allele distribution in HLs with Han Chinese (CHB+CHS) population and Japanese (JPT) population.

TERF2								
**SNP**	**Genotype/ Allele**	**CHB+CHS (Ref, n=204)**	**JPT (Ref, n=104)**	**HLs (n=200)**	**HLs vs CHB+CHS**		**HLs vs JPT**	
					**p value**	**OR (95%CI)**	**p value**	**OR (95%CI)**
rs3785073	GG	171 (82.2%)	86 (82.7%)	108 (54.0%)	Ref	-	Ref	-
	GA	34 (16.3%)	18 (17.3%)	80 (40.0%)	<0.000001	3.73 (2.33-5.95)	1.20×10^{-5}	3.54 (1.97-6.35)
	AA	3 (1.4%)	0 (0.0%)	12 (6.0%)	0.002	6.33 (1.75-22.96)	*	-
	G	376 (90.4%)	190 (91.3%)	296 (74.0%)	Ref	-	Ref	-
	A	40 (9.6%)	18 (8.7%)	104 (26.0%)	<0.000001	3.30 (2.23-4.90)	<0.000001	3.71 (2.18-6.32)
rs8061352	TT	171 (82.2%)	86 (82.7%)	107 (53.5%)	Ref	-	Ref	-
	TA	34 (16.3%)	18 (17.3%)	80 (40.0%)	<0.000001	3.76 (2.35-6.01)	1.10×10^{-5}	3.57 (1.99-6.41)
	AA	3 (1.4%)	0 (0.0%)	13 (6.5%)	0.001	6.93 (1.93-24.87)	*	-
	T	376 (90.4%)	190 (91.3%)	294 (73.5%)	Ref	-	Ref	-
	A	40 (9.6%)	18 (8.7%)	106 (26.5%)	<0.000001	3.39 (2.29-5.03)	<0.000001	3.81 (2.24-6.48)
rs153058	TT	65 (31.3%)	32 (30.8%)	92 (46.0%)	Ref	-	Ref	-
	TC	103 (49.5%)	52 (50.0%)	84 (42.0%)	0.011	0.58 (0.38-0.89)	0.032	0.56 (0.33-0.96)
	CC	40 (19.2%)	20 (19.2%)	24 (12.0%)	0.004	0.42 (0.23-0.77)	0.015	0.42 (0.20-0.86)
	T	233 (56.0%)	116 (55.8%)	268 (67.0%)	Ref	-	Ref	-
	C	183 (44.0%)	92 (44.2%)	132 (33.0%)	0.001	0.63 (0.47-0.83)	0.006	0.62 (0.44-0.88)
rs113062367	TT	208 (100%)	104 (100%)	146 (73.0%)	Ref	-	Ref	-
	TC	0 (0%)	0 (0%)	49 (24.5%)	*	-	*	-
	CC	0 (0%)	0 (0%)	5 (2.5%)	*	-	*	-
	T	416 (100.0%)	208 (100.0%)	341 (85.3%)	Ref	-	Ref	-
	C	0 (0.0%)	0 (0.0%)	59 (14.8%)	*	-	*	-

SNP	Genotype/ Allele	CHB+CHS (Ref, n=204)	JPT (Ref, n=104)	HLs (n=200)	HLs vs CHB+CHS		HLs vs JPT	
					p value	OR (95%CI)	p value	OR (95%CI)
rs4783704	CC	138 (66.3%)	80 (76.9%)	103 (51.5%)	Ref	-	Ref	-
	CT	64 (30.8%)	23 (22.1%)	81 (40.5%)	0.012	1.70 (1.12-2.57)	2.45×10^{-4}	2.74 (1.58-4.73)
	TT	6 (2.9%)	1 (1.0%)	16 (8.0%)	0.007	3.57 (1.35-9.45)	0.002	12.43 (1.61-95.70)
	C	340 (81.7%)	183 (88.0%)	287 (71.8%)	Ref	-	Ref	-
	T	76 (18.3%)	25 (12.0%)	113 (28.3%)	0.001	1.76 (1.27-2.45)	6.00×10^{-6}	2.88 (1.80-4.62)

SISA online tool was used to obtain p values. Significance was maintained at ≤0.05 after FDR correction. *Analysis could not be performed because the representatives of a genotype and/or allele were absent in a study group. *TERF2*, telomere repeat binding factor 2; CHB+CHS, Han Chinese population comprising of Han Chinese in Beijing (CHB) and Southern Han Chinese (CHS); JPT, Japanese population; HLs, highlanders; Ref, reference; n, number of samples; OR, odds ratio; CI, confidence interval.

Supplementary Table S6: Genotype and allele distribution of 7 SNPs of *POT1*

[A] Comparison of genotype and allele distribution among HAPE-f, HAPE-p and HLs.

POT1											
**SNP**	**Genetic Model**	**Genotype/ Allele**	**HAPE-f (n=210)**	**HAPE-p (n=183)**	**HLs (n=200)**	**HAPE-p vs HAPE-f**		**HLs vs HAPE-f**		**HAPE-p vs HLs**	
						**p value**	**OR (95%CI)**	**p value**	**OR (95%CI)**	**p value**	**OR (95%CI)**
rs727505	Codominant	CC	116 (55.3%)	95 (51.9%)	113 (56.5%)	Ref	-	Ref	-	Ref	-
		CT	83 (39.5%)	84 (45.9%)	71 (35.5%)	0.010	1.88 (1.16-3.05)	0.773	1.11 (0.54-2.28)	0.083	1.59 (0.94-2.69)
		TT	11 (5.2%)	4 (2.2%)	16 (8.0%)	0.258	0.47 (0.13-1.74)	0.988	1.01 (0.27-3.76)	0.112	0.34 (0.09-1.28)
	Additive	C	315 (75.0%)	274 (74.9%)	297 (74.2%)	Ref	-	Ref	-	Ref	-
		T	105 (25.0%)	92 (25.1%)	103 (25.8%)	0.204	1.27 (0.88-1.83)	0.865	1.05 (0.60-1.82)	0.754	1.07 (0.71-1.61)
	Dominant	CC	116 (55.3%)	95 (51.9%)	113 (56.5%)	Ref	-	Ref	-	Ref	-
		CT+TT	94 (44.7%)	88 (48.1%)	87 (43.5%)	0.031	1.67 (1.05-2.66)	0.824	1.08 (0.55-2.11)	0.223	1.37 (0.83-2.28)
	Recessive	CC+CT	199 (94.8%)	179 (97.8%)	184 (92.0%)	Ref	-	Ref	-	Ref	-
		TT	11 (5.2%)	4 (2.2%)	16 (8.0%)	0.134	0.35 (0.09-1.38)	0.971	0.97 (0.23-4.09)	0.053	0.27 (0.07-1.02)
	Overdominant	CC+TT	127 (60.5%)	99 (54.1%)	129 (64.5%)	Ref	-	Ref	-	Ref	-
		CT	83 (39.5%)	84 (45.9%)	71 (35.5%)	0.006	1.94 (1.21-3.10)	0.805	1.09 (0.55-2.17)	0.038	1.73 (1.03-2.92)
rs17246404	Codominant	CC	122 (58.1%)	107 (58.5%)	120 (60.0%)	Ref	-	Ref	-	Ref	-
		CT	76 (36.2%)	65 (35.5%)	60 (30.0%)	0.456	1.20 (0.74-1.95)	0.359	0.70 (0.33-1.49)	0.159	1.49 (0.86-2.58)
		TT	12 (5.7%)	11 (6.0%)	20 (10.0%)	0.686	0.82 (0.31-2.16)	0.884	0.91 (0.27-3.10)	0.477	0.71 (0.28-1.83)
	Additive	C	320 (76.2%)	279 (76.2%)	300 (75.0%)	Ref	-	Ref	-	Ref	-
		T	100 (23.8%)	87 (23.8%)	100 (25.0%)	0.871	1.03 (0.71-1.50)	0.438	0.80 (0.46-1.40)	0.725	1.08 (0.71-1.63)
	Dominant	CC	122 (58.1%)	107 (58.5%)	120 (60.0%)	Ref	-	Ref	-	Ref	-
		CT+TT	88 (41.9%)	76 (41.5%)	80 (40.0%)	0.625	1.12 (0.71-1.77)	0.363	0.73 (0.37-1.44)	0.325	1.30 (0.77-2.17)
	Recessive	CC+CT	198 (94.3%)	172 (94.0%)	180 (90.0%)	Ref	-	Ref	-	Ref	-
		TT	12 (5.7%)	11 (6.0%)	20 (10.0%)	0.532	0.73 (0.27-1.97)	0.949	0.96 (0.26-3.47)	0.314	0.62 (0.24-1.58)
	Overdominant	CC+TT	134 (63.8%)	118 (64.5%)	140 (70.0%)	Ref	-	Ref	-	Ref	-
		CT	76 (36.2%)	65 (35.5%)	60 (30.0%)	0.424	1.21 (0.76-1.94)	0.357	0.71 (0.35-1.46)	0.109	1.56 (0.91-2.69)

SNP	Genetic Model	Genotype/ Allele	HAPE-f (n=210)	HAPE-p (n=183)	HLs (n=200)	HAPE-p vs HAPE-f		HLs vs HAPE-f		HAPE-p vs HLs	
						p value	OR (95%CI)	p value	OR (95%CI)	p value	OR (95%CI)
rs7794637	Codominant	CC	90 (42.9%)	63 (34.4%)	105 (52.5%)	Ref	-	Ref	-	Ref	-
		CT	95 (45.2%)	88 (48.1%)	82 (41.0%)	0.682	0.90 (0.54-1.49)	0.137	0.59 (0.29-1.19)	0.003	2.30 (1.32-4.00)
		TT	25 (11.9%)	32 (17.5%)	13 (6.5%)	0.297	1.43 (0.73-2.81)	0.010	0.18 (0.05-0.66)	0.003	4.22 (1.62-11.02)
	Additive	C	275 (65.5%)	214 (58.5%)	292 (73.0%)	Ref	-	Ref	-	Ref	-
		T	145 (34.5%)	152 (41.5%)	108 (27.0%)	0.480	1.13 (0.81-1.57)	0.010	0.51 (0.31-0.85)	2.62×10^{-4}	2.04 (1.39-2.99)
	Dominant	CC	90 (42.9%)	63 (34.4%)	105 (52.5%)	Ref	-	Ref	-	Ref	-
		CT+TT	120 (57.1%)	120 (65.6%)	95 (47.5%)	0.901	1.03 (0.65-1.64)	0.037	0.49 (0.25-0.96)	4.82×10^{-4}	2.54 (1.51-4.29)
	Recessive	CC+CT	185 (88.1%)	151 (82.5%)	187 (93.5%)	Ref	-	Ref	-	Ref	-
		TT	25 (11.9%)	32 (17.5%)	13 (6.5%)	0.239	1.46 (0.78-2.75)	0.037	0.30 (0.10-0.93)	0.027	2.58 (1.11-5.98)
	Overdominant	CC+TT	115 (54.8%)	95 (51.9%)	118 (59.0%)	Ref	-	Ref	-	Ref	-
		CT	95 (45.2%)	88 (48.1%)	82 (41.0%)	0.466	0.84 (0.53-1.33)	0.438	0.77 (0.40-1.49)	0.039	1.72 (1.03-2.89)
rs116916706	Codominant	CC	155 (73.8%)	130 (71.0%)	143 (71.5%)	Ref	-	Ref	-	Ref	-
		CA	49 (23.3%)	50 (27.3%)	54 (27.0%)	0.370	1.28 (0.75-2.19)	0.758	1.14 (0.49-2.68)	0.552	1.19 (0.67-2.13)
		AA	6 (2.9%)	3 (1.7%)	3 (1.5%)	0.140	0.28 (0.05-1.52)	0.093	0.15 (0.02-1.37)	0.175	0.28 (0.04-1.76)
	Additive	C	359 (85.5%)	310 (84.7%)	340 (85.0%)	Ref	-	Ref	-	Ref	-
		A	61 (14.5%)	56 (15.3%)	60 (15.0%)	0.935	0.98 (0.62-1.55)	0.360	0.72 (0.36-1.45)	0.942	0.98 (0.59-1.62)
	Dominant	CC	155 (73.8%)	130 (71.0%)	143 (71.5%)	Ref	-	Ref	-	Ref	-
		CA+AA	55 (26.2%)	53 (29.0%)	57 (28.5%)	0.664	1.12 (0.67-1.86)	0.731	0.87 (0.41-1.88)	0.774	1.09 (0.62-1.90)
	Recessive	CC+CA	204 (97.1%)	180 (98.3%)	197 (98.5%)	Ref	-	Ref	-	Ref	-
		AA	6 (2.9%)	3 (1.7%)	3 (1.5%)	0.110	0.24 (0.04-1.37)	0.077	0.14 (0.02-1.24)	0.170	0.28 (0.05-1.73)
	Overdominant	CC+AA	161 (76.7%)	133 (72.7%)	146 (73.0%)	Ref	-	Ref	-	Ref	-
		CA	49 (23.3%)	50 (27.3%)	54 (27.0%)	0.325	1.30 (0.77-2.19)	0.715	1.16 (0.52-2.56)	0.504	1.22 (0.68-2.16)

SNP	Genetic Model	Genotype/ Allele	HAPE-f (n=210)	HAPE-p (n=183)	HLs (n=200)	HAPE-p vs HAPE-f		HLs vs HAPE-f		HAPE-p vs HLs	
						p value	OR (95%CI)	p value	OR (95%CI)	p value	OR (95%CI)
rs4360236	Codominant	CC	110 (52.4%)	97 (53.0%)	108 (54.0%)	Ref	-	Ref	-	Ref	-
		CT	81 (38.6%)	68 (37.2%)	77 (38.5%)	0.664	0.90 (0.54-1.48)	0.675	0.86 (0.42-1.77)	0.542	1.18 (0.69-2.00)
		TT	19 (9.0%)	18 (9.8%)	15 (7.5%)	0.997	1.00 (0.47-2.14)	0.100	0.39 (0.13-1.20)	0.673	0.83 (0.34-2.01)
	Additive	C	301 (71.7%)	262 (71.6%)	293 (73.2%)	Ref	-	Ref	-	Ref	-
		T	119 (28.3%)	104 (28.4%)	107 (26.8%)	0.804	0.96 (0.67-1.36)	0.114	0.66 (0.39-1.11)	0.954	1.01 (0.68-1.50)
	Dominant	CC	110 (52.4%)	97 (53.0%)	108 (54.0%)	Ref	-	Ref	-	Ref	-
		CT+TT	100 (47.6%)	86 (47.0%)	92 (46.0%)	0.721	0.92 (0.59-1.45)	0.313	0.71 (0.37-1.38)	0.685	1.11 (0.67-1.84)
	Recessive	CC+CT	191 (91.0%)	165 (90.2%)	185 (92.5%)	Ref	-	Ref	-	Ref	-
		TT	19 (9.0%)	18 (9.8%)	15 (7.5%)	0.948	1.03 (0.49-2.17)	0.099	0.40 (0.13-1.19)	0.558	0.77 (0.32-1.84)
	Overdominant	CC+TT	129 (61.4%)	115 (62.8%)	123 (61.5%)	Ref	-	Ref	-	Ref	-
		CT	81 (38.6%)	68 (37.2%)	77 (38.5%)	0.681	0.91 (0.57-1.45)	0.998	1.00 (0.51-1.98)	0.446	1.22 (0.73-2.06)
rs7782410	Codominant	GG	80 (38.1%)	70 (38.3%)	58 (29.0%)	Ref	-	Ref	-	Ref	-
		GA	103 (49.0%)	89 (48.6%)	96 (48.0%)	0.597	1.14 (0.70-1.88)	0.056	2.15 (0.98-4.72)	0.459	0.80 (0.45-1.43)
		AA	27 (12.9%)	24 (13.1%)	46 (23.0%)	0.610	1.19 (0.60-2.36)	0.002	4.20 (1.67-10.58)	0.003	0.32 (0.15-0.69)
	Additive	G	263 (62.6%)	229 (62.6%)	212 (53.0%)	Ref	-	Ref	-	Ref	-
		A	157 (37.4%)	137 (37.4%)	188 (47.0%)	0.490	1.12 (0.81-1.56)	0.001	2.26 (1.40-7.63)	0.006	0.60 (0.42-0.86)
	Dominant	GG	80 (38.1%)	70 (38.3%)	58 (29.0%)	Ref	-	Ref	-	Ref	-
		GA+AA	130 (61.9%)	113 (61.7%)	142 (71.0%)	0.535	1.16 (0.73-1.85)	0.007	2.73 (1.31-5.71)	0.093	0.63 (0.37-1.08)
	Recessive	GG+GA	183 (87.1%)	159 (86.9%)	154 (77.0%)	Ref	-	Ref	-	Ref	-
		AA	27 (12.9%)	24 (13.1%)	46 (23.0%)	0.614	1.19 (0.61-2.30)	0.007	3.06 (1.36-6.87)	0.003	0.36 (0.19-0.71)
	Overdominant	GG+AA	107 (51.0%)	94 (51.4%)	104 (52.0%)	Ref	-	Ref	-	Ref	-
		GA	103 (49.0%)	89 (48.6%)	96 (48.0%)	0.793	1.06 (0.68-1.67)	0.643	1.17 (0.61-2.26)	0.474	1.20 (0.73-1.99)

SNP	Genetic Model	Genotype/ Allele	HAPE-f (n=210)	HAPE-p (n=183)	HLs (n=200)	HAPE-p vs HAPE-f		HLs vs HAPE-f		HAPE-p vs HLs	
						p value	OR (95%CI)	p value	OR (95%CI)	p value	OR (95%CI)
rs10244797	Codominant	TT	68 (32.4%)	64 (35.0%)	64 (32.0%)	Ref	-	Ref	-	Ref	-
		TC	109 (51.9%)	86 (47.0%)	90 (45.0%)	0.858	1.05 (0.63-1.75)	0.422	1.37 (0.64-2.94)	0.954	1.02 (0.57-1.81)
		CC	33 (15.7%)	33 (18.0%)	46 (23.0%)	0.596	1.19 (0.63-2.23)	0.109	2.02 (0.85-4.79)	0.224	0.65 (0.32-1.31)
	Additive	T	245 (58.3%)	214 (58.5%)	218 (54.5%)	Ref	-	Ref	-	Ref	-
		C	175 (41.7%)	152 (41.5%)	182 (45.5%)	0.565	1.10 (0.80-1.52)	0.090	1.49 (0.94-2.38)	0.269	0.82 (0.57-1.17)
	Dominant	TT	68 (32.4%)	64 (35.0%)	64 (32.0%)	Ref	-	Ref	-	Ref	-
		TC+CC	142 (67.6%)	119 (65.0%)	136 (68.0%)	0.752	1.08 (0.67-1.74)	0.222	1.55 (0.77-3.15)	0.652	0.88 (0.52-1.51)
	Recessive	TT+TC	177 (84.3%)	150 (82.0%)	154 (77.0%)	Ref	-	Ref	-	Ref	-
		CC	33 (15.7%)	33 (18.0%)	46 (23.0%)	0.502	1.23 (0.68-2.23)	0.124	1.87 (0.84-4.13)	0.161	0.64 (0.34-1.20)
	Overdominant	TT+CC	101 (48.1%)	97 (53.0%)	110 (55.0%)	Ref	-	Ref	-	Ref	-
		TC	109 (51.9%)	86 (47.0%)	90 (45.0%)	0.836	0.95 (0.61-1.50)	0.935	0.97 (0.50-1.89)	0.481	1.20 (0.72-1.98)

SPSS 16.0 was used to obtain p values after adjusting with age and gender. Significance was maintained at ≤0.05 after FDR correction. *POT1*, protection of telomere 1; HAPE-f, HAPE-free; HAPE-p, HAPE-patients; HLs, Highlanders; n, number of samples; OR, odds ratio; CI, confidence interval; Ref, reference.

[B] Comparison of genotype and allele distribution in HLs with Han Chinese (CHB+CHS) population and Japanese (JPT) population.

POT1								
**SNP**	**Genotype/ Allele**	**CHB+CHS (Ref, n=204)**	**JPT (Ref, n=104)**	**HLs (n=200)**	**HLs vs CHB+CHS**		**HLs vs JPT**	
					**p value**	**OR (95%CI)**	**p value**	**OR (95%CI)**
rs727505	CC	89 (42.8%)	47 (45.2%)	113 (56.5%)	Ref	-	Ref	-
	CT	94 (45.2%)	45 (43.3%)	71 (35.5%)	0.014	0.59 (0.39-0.90)	0.101	0.66 (0.40-1.09)
	TT	25 (12.0%)	12 (11.5%)	16 (8.0%)	0.048	0.50 (0.25-1.00)	0.156	0.55 (0.24-1.26)
	C	272 (65.4%)	139 (66.8%)	297 (74.3%)	Ref	-	Ref	-
	T	144 (34.6%)	69 (33.2%)	103 (25.8%)	0.006	0.66 (0.48-0.89)	0.054	0.70 (0.49-1.01)
rs17246404	CC	89 (42.8%)	47 (45.2%)	120 (60.0%)	Ref	-	Ref	-
	CT	94 (45.2%)	45 (43.3%)	60 (30.0%)	0.001	0.47 (0.31-0.72)	0.013	0.52 (0.31-0.87)
	TT	25 (12.0%)	12 (11.5%)	20 (10.0%)	0.113	0.59 (0.31-1.14)	0.288	0.65 (0.30-1.44)
	C	272 (65.4%)	139 (66.8%)	300 (75.0%)	Ref	-	Ref	-
	T	144 (34.6%)	69 (33.2%)	100 (25.0%)	0.003	0.63 (0.47-0.85)	0.033	0.67 (0.47-0.97)
rs7794637	CC	143 (68.8%)	70 (67.3%)	105 (52.5%)	Ref	-	Ref	-
	CT	60 (28.8%)	27 (26.0%)	82 (41.0%)	0.003	1.86 (1.23-2.83)	0.008	2.02 (1.19-3.44)
	TT	5 (2.4%)	7 (6.7%)	13 (6.5%)	0.024	3.54 (1.23-10.24)	0.665	1.24 (0.47-3.26)
	C	346 (83.2%)	167 (80.3%)	292 (73.0%)	Ref	-	Ref	-
	T	70 (16.8%)	41 (19.7%)	108 (27.0%)	4.35×10^{-4}	1.83 (1.30-2.57)	0.047	1.51 (1.00-2.26)
rs116916706	CC	208 (100%)	104 (100%)	143 (71.5%)	Ref	-	Ref	-
	CA	0 (0%)	0 (0%)	54 (27.0%)	*	-	*	-
	AA	0 (0%)	0 (0%)	3 (1.5%)	*	-	*	-
	C	416 (100.0%)	208 (100.0%)	340 (85.0%)	Ref	-	Ref	-
	A	0 (0.0%)	0 (0.0%)	60 (15.0%)	*	-	*	-

SNP	Genotype/ Allele	CHB+CHS (Ref, n=204)	JPT (Ref, n=104)	HLs (n=200)	HLs vs CHB+CHS		HLs vs JPT	
					p value	OR (95%CI)	p value	OR (95%CI)
rs4360236	CC	143 (68.8%)	70 (67.3%)	108 (54.0%)	Ref	-	Ref	-
	CT	59 (28.4%)	27 (26.0%)	77 (38.5%)	0.011	1.73 (1.13-2.63)	0.023	1.85 (1.09-3.15)
	TT	6 (2.9%)	7 (6.7%)	15 (7.5%)	0.012	3.31 (1.24-8.81)	0.495	1.39 (0.54-3.58)
	C	345 (82.9%)	167 (80.3%)	293 (73.3%)	Ref	-	Ref	-
	T	71 (17.1%)	41 (19.7%)	107 (26.8%)	0.001	1.77 (1.27-2.49)	0.055	1.49 (0.99-2.24)
rs7782410	GG	70 (33.7%)	36 (34.6%)	58 (29.0%)	Ref	-	Ref	-
	GA	102 (49.0%)	47 (45.2%)	96 (48.0%)	0.575	1.14 (0.73-1.77)	0.391	1.27 (0.74-2.18)
	AA	36 (17.3%)	21 (20.2%)	46 (23.0%)	0.127	1.54 (0.88-2.69)	0.363	1.36 (0.70-2.64)
	G	242 (58.2%)	119 (57.2%)	212 (53.0%)	Ref	-	Ref	-
	A	174 (41.8%)	89 (42.8%)	188 (47.0%)	0.137	1.23 (0.94-1.63)	0.323	1.19 (0.85-1.66)
rs10244797	TT	73 (35.1%)	39 (37.5%)	64 (32.0%)	Ref	-	Ref	-
	TC	102 (49.0%)	45 (43.3%)	90 (45.0%)	1.000	1.01 (0.65-1.56)	0.469	1.22 (0.71-2.08)
	CC	33 (15.9%)	20 (19.2%)	46 (23.0%)	0.103	1.59 (0.91-2.78)	0.314	1.40 (0.73-2.71)
	T	248 (59.6%)	123 (59.1%)	218 (54.5%)	Ref	-	Ref	-
	C	168 (40.4%)	85 (40.9%)	182 (45.5%)	0.140	1.23 (0.93-1.63)	0.275	1.21 (0.86-1.70)

SISA online tool was used to obtain p values. Significance was maintained at ≤0.05 after FDR correction. *Analysis could not be performed because the representatives of a genotype and/or allele were absent in a study group. *POT1*, protection of telomere 1; CHB+CHS, Han Chinese population comprising of Han Chinese in Beijing (CHB) and Southern Han Chinese (CHS); JPT, Japanese population; HLs, highlanders; Ref, reference; n, number of samples; OR, odds ratio; CI, confidence interval.

Supplementary Table S7: Genotype and allele distribution of 2 SNPs of *ACD*

[A] Comparison of genotype and allele distribution among HAPE-f, HAPE-p and HLs.

ACD											
**SNP**	**Genetic Model**	**Genotype/ Allele**	**HAPE-f (n=210)**	**HAPE-p (n=183)**	**HLs (n=200)**	**HAPE-p vs HAPE-f**		**HLs vs HAPE-f**		**HAPE-p vs HLs**	
						**p value**	**OR (95%CI)**	**p value**	**OR (95%CI)**	**p value**	**OR (95%CI)**
rs72556537	Codominant	TT	148 (70.4%)	151 (82.5%)	145 (72.5%)	Ref	-	Ref	-	Ref	-
		TA	52 (24.8%)	31 (17.0%)	48 (24.0%)	0.073	0.59 (0.34-1.05)	0.138	0.53 (0.23-1.23)	0.183	0.65 (0.34-1.23)
		AA	10 (4.8%)	1 (0.5%)	7 (3.5%)	0.038	0.11 (0.01-0.88)	0.346	1.89 (0.50-7.08)	0.008	0.05 (0.01-0.45)
	Additive	T	348 (82.9%)	333 (91.0%)	338 (84.5%)	Ref	-	Ref	-	Ref	-
		A	72 (17.1%)	33 (9.0%)	62 (15.5%)	0.003	0.48 (0.29-0.78)	0.778	0.91 (0.49-1.71)	0.002	0.43 (0.25-0.74)
	Dominant	TT	148 (70.4%)	151 (82.5%)	145 (72.5%)	Ref	-	Ref	-	Ref	-
		TA+AA	62 (29.6%)	32 (17.5%)	55 (27.5%)	0.015	0.51 (0.29-0.88)	0.369	0.71 (0.34-1.49)	0.027	0.50 (0.27-0.92)
	Recessive	TT+TA	200 (95.2%)	182 (99.5%)	193 (96.5%)	Ref	-	Ref	-	Ref	-
		AA	10 (4.8%)	1 (0.5%)	7 (3.5%)	0.046	0.12 (0.01-0.97)	0.265	2.10 (0.57-7.72)	0.007	0.05 (0.01-0.43)
	Overdominant	TT+AA	158 (75.2%)	152 (83.0%)	152 (76.0%)	Ref	-	Ref	-	Ref	-
		TA	52 (24.8%)	31 (17.0%)	48 (24.0%)	0.115	0.63 (0.36-1.12)	0.115	0.51 (0.23-1.18)	0.278	0.71 (0.38-1.32)
rs6979	Codominant	GG	67 (31.9%)	54 (29.5%)	18 (9.0%)	Ref	-	Ref	-	Ref	-
		GA	110 (52.4%)	101 (55.2%)	74 (37.0%)	0.828	1.06 (0.64-1.74)	0.011	4.54 (1.41-14.62)	0.334	0.68 (0.31-1.49)
		AA	33 (15.7%)	28 (15.3%)	108 (54.0%)	0.982	1.01 (0.50-2.02)	3.05×10^{-7}	41.88 (10.03-174.91)	3.74×10^{-8}	0.07 (0.03-0.18)
	Additive	G	244 (58.1%)	209 (57.1%)	110 (27.5%)	Ref	-	Ref	-	Ref	-
		A	176 (41.9%)	157 (42.9%)	290 (72.5%)	0.844	1.03 (0.75-1.43)	3.34×10^{-12}	7.34 (4.19-12.86)	5.62×10^{-12}	0.26 (0.18-0.38)
	Dominant	GG	67 (31.9%)	54 (29.5%)	18 (9.0%)	Ref	-	Ref	-	Ref	-
		GA+AA	143 (68.1%)	129 (70.5%)	182 (91.0%)	0.820	1.06 (0.65-1.72)	1.42×10^{-5}	11.69 (3.85-35.48)	4.92×10^{-4}	0.29 (0.14-0.58)
	Recessive	GG+GA	177 (84.3%)	155 (84.7%)	92 (46.0%)	Ref	-	Ref	-	Ref	-
		AA	33 (15.7%)	28 (15.3%)	108 (54.0%)	0.929	1.03 (0.55-1.91)	2.52×10^{-10}	16.20 (6.83-38.39)	2.87×10^{-12}	0.11 (0.06-0.20)
	Overdominant	GG+AA	100 (47.6%)	82 (44.8%)	126 (63.0%)	Ref	-	Ref	-	Ref	-
		GA	110 (52.4%)	101 (55.2%)	74 (37.0%)	0.883	1.03 (0.66-1.63)	0.004	0.36 (0.18-0.73)	2.63×10^{-5}	3.23 (1.87-5.58)

SPSS 16.0 was used to obtain p values after adjusting with age and gender. Significance was maintained at ≤0.05 after FDR correction. *ACD,* ACD shelterin complex subunit and telomerase recruitment factor; HAPE-f, HAPE-free; HAPE-p, HAPE-patients; HLs, Highlanders; n, number of samples; OR, odds ratio; CI, confidence interval; Ref, reference.

[B] Comparison of genotype and allele distribution in HLs with Han Chinese (CHB+CHS) population and Japanese (JPT) population.

ACD								
**SNP**	**Genotype/ Allele**	**CHB+CHS (Ref, n=204)**	**JPT (Ref, n=104)**	**HLs (n=200)**	**HLs vs CHB+CHS**		**HLs vs JPT**	
					**p value**	**OR (95%CI)**	**p value**	**OR (95%CI)**
rs72556537	TT	0 (0%)	0 (0%)	145 (72.5%)	Ref	-	Ref	-
	TA	0 (0%)	4 (3.8%)	48 (24.0%)	*	-	*	-
	AA	208 (100%)	100 (96.2%)	7 (3.5%)	*	-	*	-
	T	0 (0.0%)	4 (1.9%)	338 (84.5%)	Ref	-	Ref	-
	A	416 (100.0%)	204 (98.1%)	62 (15.5%)	*	-	<0.000001	0.004 (0.001-0.01)
rs6979	GG	4 (1.9%)	4 (3.8%)	18 (9.0%)	Ref	-	Ref	-
	GA	50 (24.0%)	22 (21.2%)	74 (37.0%)	0.056	3.04 (0.97-9.52)	0.779	1.34 (0.41-4.37)
	AA	154 (74.0%)	78 (75.0%)	108 (54.0%)	2.50×10^{-4}	0.16 (0.05-0.47)	0.031	0.31 (0.10-0.95)
	G	58 (13.9%)	30 (14.4%)	110 (27.5%)	Ref	-	Ref	-
	A	358 (86.1%)	178 (85.6%)	290 (72.5%)	2.00×10^{-6}	0.43 (0.30-0.61)	2.80×10^{-4}	0.44 (0.29-0.69)

SISA online tool was used to obtain p values. Significance was maintained at ≤0.05 after FDR correction. *Analysis could not be performed because the representatives of a genotype and/or allele were absent in a study group. *ACD,* ACD shelterin complex subunit and telomerase recruitment factor; CHB+CHS, Han Chinese population comprising of Han Chinese in Beijing (CHB) and Southern Han Chinese (CHS); JPT, Japanese population; HLs, highlanders; Ref, reference; n, number of samples; OR, odds ratio; CI, confidence interval.

Supplementary Table S8: Genotype and allele distribution of 4 SNPs of *TERF2IP*

[A] Comparison of genotype and allele distribution among HAPE-f, HAPE-p and HLs.

TERF2IP											
**SNP**	**Genetic Model**	**Genotype/ Allele**	**HAPE-f (n=210)**	**HAPE-p (n=183)**	**HLs (n=200)**	**HAPE-p vs HAPE-f**		**HLs vs HAPE-f**		**HAPE-p vs HLs**	
						**p value**	**OR (95%CI)**	**p value**	**OR (95%CI)**	**p value**	**OR (95%CI)**
rs76937292	Codominant	CC	202 (96.2%)	169 (92.3%)	190 (95.0%)	Ref	-	Ref	-	Ref	-
		CT	4 (1.9%)	6 (3.3%)	2 (1.0%)	0.226	0.38 (0.08-1.82)	3.29×10^{-4}	0.01 (0.001-0.11)	0.227	3.25 (0.48-22.06)
		TT	4 (1.9%)	8 (4.4%)	8 (4.0%)	0.655	1.38 (0.34-5.59)	0.306	0.36 (0.05-2.56)	0.931	0.95 (0.27-3.33)
	Additive	C	408 (97.1%)	344 (94.0%)	382 (95.5%)	Ref	-	Ref	-	Ref	-
		T	12 (2.9%)	22 (6.0%)	18 (4.5%)	0.913	1.05 (0.46-2.41)	0.004	0.20 (0.06-0.60)	0.655	1.20 (0.54-2.67)
	Dominant	CC	202 (96.2%)	169 (92.3%)	190 (95.0%)	Ref	-	Ref	-	Ref	-
		CT+TT	8 (3.8%)	14 (7.7%)	10 (5.0%)	0.781	0.86 (0.30-2.47)	0.003	0.11 (0.02-0.47)	0.508	1.42 (0.50-4.02)
	Recessive	CC+CT	206 (98.1%)	175 (95.6%)	192 (96.0%)	Ref	-	Ref	-	Ref	-
		TT	4 (1.9%)	8 (4.4%)	8 (4.0%)	0.598	1.45 (0.37-5.70)	0.439	0.48 (0.07-3.10)	0.911	0.93 (0.26-3.30)
	Overdominant	CC+TT	206 (98.1%)	177 (96.7%)	198 (99.0%)	Ref	-	Ref	-	Ref	-
		CT	4 (1.9%)	6 (3.3%)	2 (1.0%)	0.288	0.44 (0.09-2.01)	0.001	0.01 (0.001-0.15)	0.230	3.23 (0.48-21.97)
rs5929469	Codominant	GG	73 (34.8%)	40 (21.9%)	138 (69.0%)	Ref	-	Ref	-	Ref	-
		GT	105 (50.0%)	92 (50.2%)	57 (28.5%)	0.575	1.17 (0.68-2.01)	1.23×10^{-4}	0.22 (0.10-0.47)	1.00×10^{-10}	8.48 (4.43-16.20)
		TT	32 (15.2%)	51 (27.9%)	5 (2.5%)	0.037	1.98 (1.04-3.75)	8.62×10^{-6}	0.02 (0.004-0.12)	1.03×10^{-8}	69.64 (16.29-297.67)
	Additive	G	251 (59.8%)	172 (47.0%)	333 (83.2%)	Ref	-	Ref	-	Ref	-
		T	169 (40.2%)	194 (53.0%)	67 (16.8%)	0.082	1.33 (0.96-1.83)	2.25×10^{-9}	0.16 (0.09-0.29)	3.19×10^{-18}	6.81 (4.42-10.49)
	Dominant	GG	73 (34.8%)	40 (21.9%)	138 (69.0%)	Ref	-	Ref	-	Ref	-
		GT+TT	137 (65.2%)	143 (78.1%)	62 (31.0%)	0.245	1.35 (0.82-2.23)	4.96×10^{-7}	0.14 (0.07-0.31)	1.00×10^{-14}	12.46 (6.58-23.60)
	Recessive	GG+GT	178 (84.8%)	132 (72.1%)	195 (97.5%)	Ref	-	Ref	-	Ref	-
		TT	32 (15.2%)	51 (27.9%)	5 (2.5%)	0.091	1.61 (0.93-2.80)	3.63×10^{-5}	0.03 (0.01-0.16)	1.12×10^{-6}	14.04 (4.85-40.66)
	Overdominant	GG+TT	105 (50.0%)	91 (49.8%)	143 (71.5%)	Ref	-	Ref	-	Ref	-
		GT	105 (50.0%)	92 (50.2%)	57 (28.5%)	0.738	0.93 (0.59-1.45)	0.013	0.42 (0.21-0.83)	6.76×10^{-6}	3.63 (2.07-6.36)

SNP	Genetic Model	Genotype/ Allele	HAPE-f (n=210)	HAPE-p (n=183)	HLs (n=200)	HAPE-p vs HAPE-f		HLs vs HAPE-f		HAPE-p vs HLs	
						p value	OR (95%CI)	p value	OR (95%CI)	p value	OR (95%CI)
rs2233810	Codominant	CC	180 (85.7%)	161 (88.0%)	188 (94.0%)	Ref	-	Ref	-	Ref	-
		CA	26 (12.4%)	17 (9.3%)	10 (5.0%)	0.852	0.93 (0.46-1.91)	0.025	0.13 (0.02-0.78)	0.049	2.94 (1.00-8.59)
		AA	4 (1.9%)	5 (2.7%)	2 (1.0%)	0.376	2.00 (0.43-9.35)	0.307	3.24 (0.34-30.78)	0.911	1.12 (0.16-7.71)
	Additive	C	386 (91.9%)	339 (92.6%)	386 (96.5%)	Ref	-	Ref	-	Ref	-
		A	34 (8.1%)	27 (7.4%)	14 (3.5%)	0.624	1.16 (0.64-1.09)	0.263	0.48 (0.13-1.74)	0.112	2.01 (0.85-4.76)
	Dominant	CC	180 (85.7%)	161 (88.0%)	188 (94.0%)	Ref	-	Ref	-	Ref	-
		CA+AA	30 (14.3%)	22 (12.0%)	12 (6.0%)	0.879	1.05 (0.55-2.03)	0.100	0.29 (0.07-1.27)	0.078	2.37 (0.91-6.18)
	Recessive	CC+CA	206 (98.1%)	178 (97.3%)	198 (99.0%)	Ref	-	Ref	-	Ref	-
		AA	4 (1.9%)	5 (2.7%)	2 (1.0%)	0.341	2.12 (0.45-9.94)	0.240	3.87 (0.41-36.88)	0.989	1.01 (0.15-7.09)
	Overdominant	CC+AA	184 (87.6%)	166 (90.7%)	190 (95.0%)	Ref	-	Ref	-	Ref	-
		CA	26 (12.4%)	17 (9.3%)	10 (5.0%)	0.800	0.91 (0.45-1.85)	0.025	0.15 (0.03-0.79)	0.051	2.86 (1.00-8.22)
rs4888444	Codominant	AA	199 (94.7%)	165 (90.2%)	187 (93.5%)	Ref	-	Ref	-	Ref	-
		AG	10 (4.8%)	17 (9.3%)	10 (5.0%)	0.259	1.68 (0.68-4.15)	0.458	1.69 (0.42-6.69)	0.206	1.89 (0.70-5.09)
		GG	1 (0.5%)	1 (0.5%)	3 (1.5%)	0.851	0.76 (0.04-13.71)	0.367	3.95 (0.20-78.34)	0.236	0.21 (0.02-2.80)
	Additive	A	408 (97.1%)	347 (94.8%)	384 (96.0%)	Ref	-	Ref	-	Ref	-
		G	12 (2.9%)	19 (5.2%)	16 (4.0%)	0.362	1.46 (0.65-3.32)	0.192	2.10 (0.69-6.44)	0.751	1.14 (0.50-2.64)
	Dominant	AA	199 (94.7%)	165 (90.2%)	187 (93.5%)	Ref	-	Ref	-	Ref	-
		AG+GG	11 (5.3%)	18 (9.8%)	13 (6.5%)	0.302	1.58 (0.66-3.75)	0.297	1.93 (0.56-6.68)	0.432	1.44 (0.58-3.59)
	Recessive	AA+AG	209 (99.5%)	182 (99.5%)	197 (98.5%)	Ref	-	Ref	-	Ref	-
		GG	1 (0.5%)	1 (0.5%)	3 (1.5%)	0.818	0.71 (0.04-13.00)	0.377	3.86 (0.19-77.57)	0.228	0.20 (0.02-2.71)
	Overdominant	AA+GG	200 (95.2%)	166 (90.7%)	190 (95.0%)	Ref	-	Ref	-	Ref	-
		AG	10 (4.8%)	17 (9.3%)	10 (5.0%)	0.255	1.69 (0.69-4.16)	0.477	1.65 (0.42-6.48)	0.194	1.93 (0.72-5.18)

SPSS 16.0 was used to obtain p values after adjusting with age and gender. Significance was maintained at ≤0.05 after FDR correction. *TERF2IP*, TERF2-interacting protein; HAPE-f, HAPE-free; HAPE-p, HAPE-patients; HLs, Highlanders; n, number of samples; OR, odds ratio; CI, confidence interval; Ref, reference.

[B] Comparison of genotype and allele distribution in HLs with Han Chinese (CHB+CHS) population and Japanese (JPT) population.

TERF2IP								
**SNP**	**Genotype/ Allele**	**CHB+CHS (Ref, n=204)**	**JPT (Ref, n=104)**	**HLs (n=200)**	**HLs vs CHB+CHS**		**HLs vs CHB+CHS**	
					**p value**	**OR (95%CI)**	**p value**	**OR (95%CI)**
rs76937292	CC	208 (100%)	104 (100%)	190 (95.0%)	Ref	-	Ref	-
	CT	0 (0%)	0 (0%)	2 (1.0%)	*	-	*	-
	TT	0 (0%)	0 (0%)	8 (4.0%)	*	-	*	-
	C	416 (100.0%)	208 (100.0%)	382 (95.5%)	Ref	-	Ref	-
	T	0 (0.0%)	0 (0.0%)	18 (4.5%)	*	-	*	-
rs59297469	GG	157 (75.5%)	80 (76.9%)	138 (69.0%)	Ref	-	Ref	-
	GT	45 (21.6%)	21 (20.2%)	57 (28.5%)	0.113	1.44 (0.92-2.27)	0.118	1.57 (0.89-2.79)
	TT	6 (2.9%)	3 (2.9%)	5 (2.5%)	1.000	0.95 (0.28-3.18)	1.000	0.97 (0.23-4.15)
	G	359 (86.3%)	181 (87.0%)	333 (83.3%)	Ref	-	Ref	-
	T	57 (13.7%)	27 (13.0%)	67 (16.8%)	0.225	1.27 (0.86-1.86)	0.223	1.35 (0.83-2.18)
rs2233810	CC	197 (94.7%)	102 (98.1%)	188 (94.0%)	Ref	-	Ref	-
	CA	11 (5.3%)	2 (1.9%)	10 (5.0%)	1.000	0.95 (0.40-2.30)	0.186	2.71 (0.58-12.62)
	AA	0 (0.0%)	0 (0.0%)	2 (1.0%)	*	-	*	-
	C	405 (97.4%)	206 (99.0%)	386 (96.5%)	Ref	-	Ref	-
	A	11 (2.6%)	2 (1.0%)	14 (3.5%)	0.478	1.34 (0.60-2.98)	0.105	3.74 (0.84-16.60)
rs4888444	AA	208 (100%)	104 (100%)	187 (93.5%)	Ref	-	Ref	-
	AG	0 (0%)	0 (0%)	10 (5.0%)	*	-	*	-
	GG	0 (0%)	0 (0%)	3 (1.5%)	*	-	*	-
	A	416 (100.0%)	208 (100.0%)	384 (96.0%)	Ref	-	Ref	-
	G	0 (0.0%)	0 (0.0%)	16 (4.0%)	*	-	*	-

SISA online tool was used to obtain p values. Significance was maintained at ≤0.05 after FDR correction. *Analysis could not be performed because the representatives of a genotype and/or allele were absent in a study group. *TERF2IP*, TERF2-interacting protein; CHB+CHS, Han Chinese population comprising of Han Chinese in Beijing (CHB) and Southern Han Chinese (CHS); JPT, Japanese population; HLs, highlanders; Ref, reference; n, number of samples; OR, odds ratio; CI, confidence interval.

Supplementary Table S9: Genotype and allele distribution of 9 SNPs of *TERT*

[A] Comparison of genotype and allele distribution among HAPE-f, HAPE-p and HLs.

TERT											
**SNP**	**Genetic Model**	**Genotype/ Allele**	**HAPE-f (n=210)**	**HAPE-p (n=183)**	**HLs (n=200)**	**HAPE-p vs HAPE-f**		**HLs vs HAPE-f**		**HAPE-p vs HLs**	
						**p value**	**OR (95%CI)**	**p value**	**OR (95%CI)**	**p value**	**OR (95%CI)**
rs2736122	Codominant	CC	128 (61.0%)	121 (66.1%)	176 (88.0%)	Ref	-	Ref	-	Ref	-
		CT	72 (34.2%)	52 (28.4%)	24 (12.0%)	0.312	0.77 (0.47-1.27)	3.54×10^{-4}	0.17 (0.07-0.46)	1.81×10^{-5}	4.59 (2.29-9.21)
		TT	10 (4.8%)	10 (5.5%)	0 (0.0%)	0.417	1.51 (0.56-4.05)	*	-	*	-
	Additive	C	328 (78.1%)	294 (80.3%)	376 (94.0%)	Ref	-	Ref	-	Ref	-
		T	92 (21.9%)	72 (19.7%)	24 (6.0%)	0.918	0.98 (0.66-1.45)	2.54×10^{-5}	0.16 (0.07-0.38)	1.80×10^{-6}	4.40 (2.40-8.09)
	Dominant	CC	128 (61.0%)	121 (66.1%)	176 (88.0%)	Ref	-	Ref	-	Ref	-
		CT+TT	82 (39.0%)	62 (33.9%)	24 (12.0%)	0.542	0.86 (0.54-1.38)	8.47×10^{-5}	0.15 (0.06-0.39)	3.81×10^{-6}	5.00 (2.53-9.90)
	Recessive	CC+CT	200 (95.2%)	173 (94.5%)	200 (100.0%)	Ref	-	Ref	-	Ref	-
		TT	10 (4.8%)	10 (5.5%)	0 (0.0%)	0.299	1.69 (0.63-4.53)	*	-	*	-
	Overdominant	CC+TT	138 (65.8%)	131 (71.6%)	176 (88.0%)	Ref	-	Ref	-	Ref	-
		CT	72 (34.2%)	52 (28.4%)	24 (12.0%)	0.255	0.75 (0.46-1.23)	0.001	0.20 (0.08-0.50)	3.13×10^{-5}	4.44 (2.20-8.97)
rs2075786	Codominant	TT	52 (24.8%)	47 (25.7%)	124 (62.0%)	Ref	-	Ref	-	Ref	-
		TC	111 (52.9%)	92 (50.3%)	64 (32.0%)	0.931	1.02 (0.59-1.79)	4.89×10^{-6}	0.15 (0.07-0.34)	2.53×10^{-7}	4.55 (2.56-8.10)
		CC	47 (22.3%)	44 (24.0%)	12 (6.0%)	0.576	1.20 (0.63-2.29)	1.44×10^{-5}	0.07 (0.02-0.23)	4.82×10^{-6}	7.27 (3.11-17.02)
	Additive	T	215 (51.2%)	186 (50.8%)	312 (78.0%)	Ref	-	Ref	-	Ref	-
		C	205 (48.8%)	180 (49.2%)	88 (22.0%)	0.543	1.10 (0.80-1.52)	6.09×10^{-9}	0.19 (0.11-0.33)	1.66×10^{-9}	3.34 (2.26-4.95)
	Dominant	TT	52 (24.8%)	47 (25.7%)	124 (62.0%)	Ref	-	Ref	-	Ref	-
		TC+CC	158 (75.2%)	136 (74.3%)	76 (38.0%)	0.807	1.07 (0.63-1.79)	5.60×10^{-8}	0.12 (0.06-0.26)	3.17×10^{-9}	5.31 (3.06-9.23)
	Recessive	TT+TC	163 (77.7%)	139 (76.0%)	188 (94.0%)	Ref	-	Ref	-	Ref	-
		CC	47 (22.3%)	44 (24.0%)	12 (6.0%)	0.443	1.23 (0.72-2.10)	0.001	0.12 (0.04-0.41)	0.003	3.44 (1.54-7.70)
	Overdominant	TT+CC	99 (47.1%)	91 (49.7%)	136 (68.0%)	Ref	-	Ref	-	Ref	-
		TC	111 (52.9%)	92 (50.3%)	64 (32.0%)	0.663	0.90 (0.58-1.42)	0.001	0.31 (0.15-0.63)	1.09×10^{-4}	2.93 (1.70-5.05)

SNP	Genetic Model	Genotype/ Allele	HAPE-f (n=210)	HAPE-p (n=183)	HLs (n=200)	HAPE-p vs HAPE-f		HLs vs HAPE-f		HAPE-p vs HLs	
						p value	OR (95%CI)	p value	OR (95%CI)	p value	OR (95%CI)
rs4246742	Codominant	AA	133 (63.4%)	121 (66.1%)	126 (63.0%)	Ref	-	Ref	-	Ref	-
		AT	66 (31.4%)	55 (30.1%)	55 (27.5%)	0.983	0.99 (0.60-1.64)	0.377	1.39 (0.67-2.91)	0.706	1.12 (0.63-1.96)
		TT	11 (5.2%)	7 (3.8%)	19 (9.5%)	0.600	0.75 (0.26-2.18)	0.594	1.39 (0.42-4.60)	0.121	0.41 (0.13-1.26)
	Additive	A	332 (79.0%)	297 (81.1%)	307 (76.8%)	Ref	-	Ref	-	Ref	-
		T	88 (21.0%)	69 (18.9%)	93 (23.2%)	0.719	0.93 (0.62-1.38)	0.317	1.32 (0.76-2.29)	0.384	0.82 (0.53-1.28)
	Dominant	AA	133 (63.4%)	121 (66.1%)	126 (63.0%)	Ref	-	Ref	-	Ref	-
		AT+TT	77 (36.6%)	62 (33.9%)	74 (37.0%)	0.852	0.96 (0.60-1.53)	0.327	1.40 (0.71-2.77)	0.817	0.94 (0.56-1.59)
	Recessive	AA+AT	199 (94.8%)	176 (96.2%)	181 (90.5%)	Ref	-	Ref	-	Ref	-
		TT	11 (5.2%)	7 (3.8%)	19 (9.5%)	0.593	0.75 (0.25-2.18)	0.670	1.30 (0.39-4.29)	0.111	0.41 (0.14-1.22)
	Overdominant	AA+TT	144 (68.6%)	128 (69.9%)	145 (72.5%)	Ref	-	Ref	-	Ref	-
		AT	66 (31.4%)	55 (30.1%)	55 (27.5%)	0.956	1.01 (0.62-1.65)	0.425	1.34 (0.65-2.77)	0.541	1.19 (0.68-2.07)
rs10069690	Codominant	CC	108 (51.4%)	98 (53.6%)	132 (66.0%)	Ref	-	Ref	-	Ref	-
		CT	87 (41.4%)	70 (38.2%)	60 (30.0%)	0.947	1.02 (0.63-1.64)	0.027	0.43 (0.21-0.91)	0.016	1.98 (1.14-3.46)
		TT	15 (7.2%)	15 (8.2%)	8 (4.0%)	0.255	1.65 (0.70-3.91)	0.401	0.49 (0.09-2.58)	0.161	2.26 (0.72-7.03)
	Additive	C	303 (72.1%)	266 (72.7%)	324 (81.0%)	Ref	-	Ref	-	Ref	-
		T	117 (27.9%)	100 (27.3%)	76 (19.0%)	0.410	1.16 (0.81-1.66)	0.036	0.53 (0.30-0.96)	0.011	1.76 (1.14-2.73)
	Dominant	CC	108 (51.4%)	98 (53.6%)	132 (66.0%)	Ref	-	Ref	-	Ref	-
		CT+TT	102 (48.6%)	85 (46.4%)	68 (34.0%)	0.683	1.10 (0.70-1.73)	0.022	0.44 (0.22-0.89)	0.009	2.03 (1.19-3.45)
	Recessive	CC+CT	195 (92.8%)	168 (91.8%)	192 (96.0%)	Ref	-	Ref	-	Ref	-
		TT	15 (7.2%)	15 (8.2%)	8 (4.0%)	0.229	1.67 (0.72-3.88)	0.629	0.66 (0.12-3.63)	0.321	1.80 (0.56-5.71)
	Overdominant	CC+TT	123 (58.6%)	113 (61.8%)	140 (70.0%)	Ref	-	Ref	-	Ref	-
		CT	87 (41.4%)	70 (38.2%)	60 (30.0%)	0.817	0.95 (0.60-1.50)	0.032	0.45 (0.22-0.93)	0.026	1.87 (1.08-3.23)

SNP	Genetic Model	Genotype/ Allele	HAPE-f (n=210)	HAPE-p (n=183)	HLs (n=200)	HAPE-p vs HAPE-f		HLs vs HAPE-f		HAPE-p vs HLs	
						p value	OR (95%CI)	p value	OR (95%CI)	p value	OR (95%CI)
rs2242652	Codominant	CC	125 (59.5%)	115 (62.8%)	135 (67.5%)	Ref	-	Ref	-	Ref	-
		CT	66 (31.4%)	53 (29.0%)	59 (29.5%)	0.565	0.86 (0.52-1.43)	0.040	0.45 (0.21-0.96)	0.359	1.30 (0.74-2.27)
		TT	19 (9.1%)	15 (8.2%)	6 (3.0%)	0.489	1.33 (0.60-2.95)	0.412	0.53 (0.11-2.44)	0.323	1.78 (0.57-5.64)
	Additive	C	316 (75.2%)	283 (77.3%)	329 (82.2%)	Ref	-	Ref	-	Ref	-
		T	104 (24.8%)	83 (22.7%)	71 (17.8%)	0.842	1.04 (0.72-1.51)	0.048	0.55 (0.30-0.99)	0.213	1.33 (0.85-2.08)
	Dominant	CC	125 (59.5%)	115 (62.8%)	135 (67.5%)	Ref	-	Ref	-	Ref	-
		CT+TT	85 (40.5%)	68 (37.2%)	65 (32.5%)	0.826	0.95 (0.60-1.51)	0.035	0.46 (0.22-0.95)	0.258	1.36 (0.80-2.32)
	Recessive	CC+CT	191 (90.9%)	168 (91.8%)	194 (97.0%)	Ref	-	Ref	-	Ref	-
		TT	19 (9.1%)	15 (8.2%)	6 (3.0%)	0.427	1.37 (0.63-2.96)	0.621	0.68 (0.15-3.14)	0.462	1.56 (0.48-5.12)
	Overdominant	CC+TT	144 (68.6%)	130 (71.0%)	141 (70.5%)	Ref	-	Ref	-	Ref	-
		CT	66 (31.4%)	53 (29.0%)	59 (29.5%)	0.467	0.83 (0.51-1.36)	0.049	0.46 (0.21-1.00)	0.413	1.26 (0.72-2.21)
rs2853676	Codominant	GG	97 (46.2%)	77 (42.1%)	118 (59.0%)	Ref	-	Ref	-	Ref	-
		GA	85 (40.5%)	75 (41.0%)	64 (32.0%)	0.361	1.26 (0.77-2.05)	0.018	0.41 (0.20-0.86)	0.009	2.13 (1.20-3.77)
		AA	28 (13.3%)	31 (16.9%)	18 (9.0%)	0.431	1.31 (0.67-2.57)	0.199	0.51 (0.18-1.43)	0.001	3.56 (1.64-7.71)
	Additive	G	279 (66.4%)	229 (62.6%)	300 (75.0%)	Ref	-	Ref	-	Ref	-
		A	141 (33.6%)	137 (37.4%)	100 (25.0%)	0.304	1.19 (0.85-1.66)	0.021	0.53 (0.31-0.91)	4.04×10^{-5}	2.31 (1.55-3.45)
	Dominant	GG	97 (46.2%)	77 (42.1%)	118 (59.0%)	Ref	-	Ref	-	Ref	-
		GA+AA	113 (53.8%)	106 (57.9%)	82 (41.0%)	0.277	1.29 (0.82-2.04)	0.013	0.42 (0.21-0.83)	0.001	2.51 (1.49-4.24)
	Recessive	GG+GA	182 (86.7%)	152 (83.1%)	182 (91.0%)	Ref	-	Ref	-	Ref	-
		AA	28 (13.3%)	31 (16.9%)	18 (9.0%)	0.656	1.16 (0.61-2.19)	0.479	0.68 (0.24-1.96)	0.004	3.19 (1.43-7.08)
	Overdominant	GG+AA	125 (59.5%)	108 (59.0%)	136 (68.0%)	Ref	-	Ref	-	Ref	-
		GA	85 (40.5%)	75 (41.0%)	64 (32.0%)	0.435	1.20 (0.76-1.91)	0.031	0.45 (0.22-0.93)	0.099	1.56 (0.92-2.64)

SNP	Genetic Model	Genotype/ Allele	HAPE-f (n=210)	HAPE-p (n=183)	HLs (n=200)	HAPE-p vs HAPE-f		HLs vs HAPE-f		HAPE-p vs HLs	
						p value	OR (95%CI)	p value	OR (95%CI)	p value	OR (95%CI)
rs2736098	Codominant	AA	57 (27.1%)	45 (24.6%)	19 (9.5%)	Ref	-	Ref	-	Ref	-
		AG	109 (51.9%)	91 (49.7%)	81 (40.5%)	0.757	1.09 (0.63-1.89)	0.096	2.35 (0.86-6.43)	0.062	0.48 (0.22-1.04)
		GG	44 (21.0%)	47 (25.7%)	100 (50.0%)	0.218	1.49 (0.79-2.81)	3.30×10^{-4}	6.99 (2.42-20.20)	2.03×10^{-4}	0.22 (0.10-0.49)
	Additive	A	223 (53.1%)	181 (49.5%)	119 (29.8%)	Ref	-	Ref	-	Ref	-
		G	197 (46.9%)	185 (50.5%)	281 (70.2%)	0.192	1.24 (0.90-1.70)	5.62×10^{-5}	2.70 (1.57-4.38)	5.013×10^{-05}	0.47 (0.32-0.67)
	Dominant	AA	57 (27.1%)	45 (24.6%)	19 (9.5%)	Ref	-	Ref	-	Ref	-
		AG+GG	153 (72.9%)	138 (75.4%)	181 (90.5%)	0.454	1.22 (0.73-2.05)	0.007	3.69 (1.43-9.52)	0.004	0.35 (0.17-0.71)
	Recessive	AA+AG	166 (79.0%)	136 (74.3%)	100 (50.0%)	Ref	-	Ref	-	Ref	-
		GG	44 (21.0%)	47 (25.7%)	100 (50.0%)	0.159	1.47 (0.86-2.50)	1.90×10^{-4}	3.89 (1.91-7.95)	3.68×10^{-4}	0.38 (0.22-0.65)
	Overdominant	AA+GG	101 (48.1%)	92 (50.3%)	119 (59.5%)	Ref	-	Ref		Ref	-
		AG	109 (51.9%)	91 (49.7%)	81 (40.5%)	0.593	0.88 (0.56-1.39)	0.176	0.64 (0.33-1.23)	0.210	1.38 (0.83-2.29)
rs2853669	Codominant	CC	67 (31.9%)	60 (32.8%)	25 (12.5%)	Ref	-	Ref	-	Ref	-
		CT	105 (50.0%)	93 (50.8%)	81 (40.5%)	0.821	0.94 (0.57-1.57)	0.129	2.04 (0.81-5.13)	0.064	0.53 (0.27-1.04)
		TT	38 (18.1%)	30 (16.4%)	94 (47.0%)	0.875	1.05 (0.54-2.05)	2.97×10^{-4}	5.98 (2.27-15.77)	1.88×10^{-6}	0.14 (0.06-0.31)
	Additive	C	239 (56.9%)	213 (58.2%)	131 (32.8%)	Ref	-	Ref	-	Ref	-
		T	181 (43.1%)	153 (41.8%)	269 (67.2%)	0.945	1.01 (0.73-1.40)	9.67×10^{-5}	2.57 (1.60-4.13)	1.48×10^{-7}	0.37 (0.26-0.54)
	Dominant	CC	67 (31.9%)	60 (32.8%)	25 (12.5%)	Ref	-	Ref	-	Ref	-
		CT+TT	143 (68.1%)	123 (67.2%)	175 (87.5%)	0.909	0.97 (0.60-1.57)	0.010	3.09 (1.31-7.30)	0.001	0.33 (0.17-0.63)
	Recessive	CC+CT	172 (81.9%)	153 (83.6%)	106 (53.0%)	Ref	-	Ref	-	Ref	-
		TT	38 (18.1%)	30 (16.4%)	94 (47.0%)	0.788	1.08 (0.60-1.96)	2.59×10^{-4}	3.93 (1.89-8.19)	8.95×10^{-7}	0.23 (0.13-0.42)
	Overdominant	CC+TT	105 (50.0%)	90 (49.2%)	119 (59.5%)	Ref	-	Ref	-	Ref	-
		CT	105 (50.0%)	93 (50.8%)	81 (40.5%)	0.756	0.93 (0.59-1.46)	0.259	0.69 (0.36-1.32)	0.082	1.57 (0.94-2.61)

SNP	Genetic Model	Genotype/ Allele	HAPE-f (n=210)	HAPE-p (n=183)	HLs (n=200)	HAPE-p vs HAPE-f		HLs vs HAPE-f		HAPE-p vs HLs	
						p value	OR (95%CI)	p value	OR (95%CI)	p value	OR (95%CI)
rs7712562	Codominant	GG	170 (81.0%)	149 (81.4%)	146 (73.0%)	Ref	-	Ref	-	Ref	-
		GA	35 (16.6%)	32 (17.5%)	45 (22.5%)	0.399	1.29 (0.71-2.36)	0.930	0.96 (0.41-2.28)	0.489	0.80 (0.43-1.50)
		AA	5 (2.4%)	2 (1.1%)	9 (4.5%)	0.437	0.47 (0.07-3.15)	0.408	0.47 (0.08-2.85)	0.301	0.37 (0.06-2.41)
	Additive	G	375 (89.3%)	330 (90.2%)	337 (84.2%)	Ref	-	Ref	-	Ref	-
		A	45 (10.7%)	36 (9.8%)	63 (15.8%)	0.810	1.07 (0.63-1.79)	0.478	0.77 (0.37-1.59)	0.252	0.73 (0.42-1.26)
	Dominant	GG	170 (81.0%)	149 (81.4%)	146 (73.0%)	Ref	-	Ref	-	Ref	-
		GA+AA	40 (19.0%)	34 (18.6%)	54 (27.0%)	0.581	1.17 (0.66-2.08)	0.674	0.84 (0.37-1.91)	0.352	0.75 (0.41-1.38)
	Recessive	GG+GA	205 (97.6%)	181 (98.9%)	191 (95.5%)	Ref	-	Ref	-	Ref	-
		AA	5 (2.4%)	2 (1.1%)	9 (4.5%)	0.406	0.45 (0.07-2.99)	0.373	0.43 (0.07-2.72)	0.345	0.40 (0.06-2.72)
	Overdominant	GG+AA	175 (83.4%)	151 (82.5%)	155 (77.5%)	Ref	-	Ref	-	Ref	-
		GA	35 (16.6%)	32 (17.5%)	45 (22.5%)	0.385	1.30 (0.72-2.35)	0.967	0.98 (0.41-2.33)	0.547	0.82 (0.44-1.55)

SPSS 16.0 was used to obtain p values after adjusting with age and gender. Significance was maintained at ≤0.05 after FDR correction. *TERT,* telomerase reverse transcriptase; HAPE-f, HAPE-free; HAPE-p, HAPE-patients; HLs, Highlanders; n, number of samples; OR, odds ratio; CI, confidence interval; Ref, reference.

[B] Comparison of genotype and allele distribution in HLs with Han Chinese (CHB+CHS) population and Japanese (JPT) population.

TERT								
**SNP**	**Genotype/ Allele**	**CHB+CHS (Ref, n=204)**	**JPT (Ref, n=104)**	**HLs (n=200)**	**HLs vs CHB+CHS**		**HLs vs JPT**	
					**p value**	**OR (95%CI)**	**p value**	**OR (95%CI)**
rs2736122	CC	183 (88.0%)	74 (71.2%)	176 (88.0%)	Ref	-	Ref	-
	CT	24 (11.5%)	30 (28.8%)	24 (12.0%)	1.000	1.04 (0.57-1.90)	2.66×10^{-4}	0.34 (0.18-0.61)
	TT	1 (0.5%)	0 (0.0%)	0 (0.0%)	*	-	*	-
	C	390 (93.8%)	178 (85.6%)	376 (94.0%)	Ref	-	Ref	-
	T	26 (6.3%)	30 (14.4%)	24 (6.0%)	0.882	0.96 (0.54-1.70)	0.001	0.38 (0.22-0.67)
rs2075786	TT	135 (64.9%)	59 (56.7%)	124 (62.0%)	Ref	-	Ref	-
	TC	66 (31.7%)	44 (42.3%)	64 (32.0%)	0.801	1.06 (0.69-1.61)	0.143	0.69 (0.42-1.13)
	CC	7 (3.4%)	1 (1.0%)	12 (6.0%)	0.198	1.87 (0.71-4.89)	0.115	5.71 (0.73-44.95)
	T	336 (80.8%)	162 (77.9%)	312 (78.0%)	Ref	-	Ref	-
	C	80 (19.2%)	46 (22.1%)	88 (22.0%)	0.328	1.18 (0.84-1.66)	1.000	0.99 (0.66-1.49)
rs4246742	AA	35 (16.8%)	13 (12.5%)	126 (63.0%)	Ref	-	Ref	-
	AT	97 (46.6%)	46 (44.2%)	55 (27.5%)	<0.000001	0.16 (0.10-0.26)	<0.000001	0.12 (0.06-0.25)
	TT	76 (36.5%)	45 (43.3%)	19 (9.5%)	<0.000001	0.07 (0.04-0.13)	<0.000001	0.04 (0.02-0.10)
	A	167 (40.1%)	72 (34.6%)	307 (76.8%)	Ref	-	Ref	-
	T	249 (59.9%)	136 (65.4%)	93 (23.3%)	<0.000001	0.20 (0.15-0.28)	<0.000001	0.16 (0.11-0.23)
rs10069690	CC	149 (71.6%)	63 (60.6%)	132 (66.0%)	Ref	-	Ref	-
	CT	55 (26.4%)	36 (34.6%)	60 (30.0%)	0.347	1.23 (0.80-1.90)	0.379	0.80 (0.48-1.33)
	TT	4 (1.9%)	5 (4.8%)	8 (4.0%)	0.241	2.26 (0.67-7.67)	0.761	0.76 (0.24-2.43)
	C	353 (84.9%)	162 (77.9%)	324 (81.0%)	Ref	-	Ref	-
	T	63 (15.1%)	46 (22.1%)	76 (19.0%)	0.143	1.31 (0.91-1.90)	0.363	0.83 (0.55-1.25)

SNP	Genotype/ Allele	CHB+CHS (Ref, n=204)	JPT (Ref, n=104)	HLs (n=200)	HLs vs CHB+CHS		HLs vs JPT	
					p value	OR (95%CI)	p value	OR (95%CI)
rs2242652	CC	149 (71.6%)	62 (59.6%)	135 (67.5%)	Ref	-	Ref	-
	CT	55 (26.4%)	37 (35.6%)	59 (29.5%)	0.446	1.18 (0.77-1.83)	0.230	0.73 (0.44-1.22)
	TT	4 (1.9%)	5 (4.8%)	6 (3.0%)	0.528	1.66 (0.46-5.99)	0.337	0.55 (0.16-1.88)
	C	353 (84.9%)	161 (77.4%)	329 (82.3%)	Ref	-	Ref	-
	T	63 (15.1%)	47 (22.6%)	71 (17.8%)	0.315	1.21 (0.83-1.75)	0.152	0.74 (0.49-1.12)
rs2853676	GG	160 (76.9%)	73 (70.2%)	118 (59.0%)	Ref	-	Ref	-
	GA	41 (19.7%)	24 (23.1%)	64 (32.0%)	0.001	2.12 (1.34-3.35)	0.074	1.65 (0.95-2.87)
	AA	7 (3.4%)	7 (6.7%)	18 (9.0%)	0.004	3.49 (1.41-8.62)	0.320	1.59 (0.63-3.99)
	G	361 (86.8%)	170 (81.7%)	300 (75.0%)	Ref	-	Ref	-
	A	55 (13.2%)	38 (18.3%)	100 (25.0%)	1.80×10^{-5}	2.19 (1.52-3.15)	0.060	1.49 (0.98-2.27)
rs2736098	AA	24 (11.5%)	8 (7.7%)	19 (9.5%)	Ref	-	Ref	-
	GA	106 (51.0%)	35 (33.7%)	81 (40.5%)	1.000	0.97 (0.50-1.88)	1.000	0.97 (0.39-2.44)
	GG	78 (37.5%)	61 (58.7%)	100 (50.0%)	0.157	1.62 (0.83-3.17)	0.410	0.69 (0.29-1.67)
	A	154 (37.0%)	51 (24.5%)	119 (29.8%)	Ref	-	Ref	-
	G	262 (63.0%)	157 (75.5%)	281 (70.3%)	0.028	1.39 (1.04-1.86)	0.173	0.77 (0.52-1.12)
rs2853669	CC	24 (11.5%)	8 (7.7%)	25 (12.5%)	Ref	-	Ref	-
	CT	107 (51.4%)	35 (33.7%)	81 (40.5%)	0.320	0.73 (0.39-1.37)	0.507	0.74 (0.30-1.80)
	TT	77 (37.0%)	61 (58.7%)	94 (47.0%)	0.625	1.17 (0.62-2.21)	0.102	0.49 (0.21-1.16)
	C	155 (37.3%)	51 (24.5%)	131 (32.8%)	Ref	-	Ref	-
	T	261 (62.7%)	157 (75.5%)	269 (67.3%)	0.177	1.22 (0.91-1.63)	0.036	0.67 (0.46-0.97)

SNP	Genotype/ Allele	CHB+CHS (Ref, n=204)	JPT (Ref, n=104)	HLs (n=200)	HLs vs CHB+CHS		HLs vs JPT	
					p value	OR (95%CI)	p value	OR (95%CI)
rs7712562	GG	175 (84.1%)	87 (83.7%)	146 (73.0%)	Ref	-	Ref	-
	GA	32 (15.4%)	15 (14.4%)	45 (22.5%)	0.041	1.69 (1.02-2.79)	0.074	1.79 (0.94-3.40)
	AA	1 (0.5%)	2 (1.9%)	9 (4.5%)	0.007	10.79 (1.35-86.15)	0.337	2.68 (0.57-12.70)
	G	382 (91.8%)	189 (90.9%)	337 (84.3%)	Ref	-	Ref	-
	A	34 (8.2%)	19 (9.1%)	63 (15.8%)	0.001	2.10 (1.35-3.27)	0.023	1.86 (1.08-3.20)

SISA online tool was used to obtain p values. Significance was maintained at ≤0.05 after FDR correction. *Analysis could not be performed because the representatives of an allele were absent in a study group. *TERT,* telomerase reverse transcriptase; CHB+CHS, Han Chinese population comprising of Han Chinese in Beijing (CHB) and Southern Han Chinese (CHS); JPT, Japanese population; HLs, highlanders; Ref, reference; n, number of samples; OR, odds ratio; CI, confidence interval.

Supplementary Table S10: Genotype and allele distribution of *TERC* SNP

[A] Comparison of genotype and allele distribution among HAPE-f, HAPE-p and HLs.

TERC											
**SNP**	**Genetic Model**	**Genotype/ Allele**	**HAPE-f (n=210)**	**HAPE-p (n=183)**	**HLs (n=200)**	**HAPE-p vs HAPE-f**		**HLs vs HAPE-f**		**HAPE-p vs HLs**	
						**p value**	**OR (95%CI)**	**p value**	**OR (95%CI)**	**p value**	**OR (95%CI)**
rs2293607	Codominant	AA	132 (62.8%)	97 (53.0%)	32 (16.0%)	Ref	-	Ref	-	Ref	-
		AG	65 (31.0%)	66 (36.1%)	101 (50.5%)	0.099	1.51 (0.93-2.47)	1.30×10^{-6}	9.04 (3.71-22.04)	3.16×10^{-7}	0.18 (0.10-0.35)
		GG	13 (6.2%)	20 (10.9%)	67 (33.5%)	0.015	2.81 (1.22-6.46)	8.04×10^{-9}	26.92 (8.79-82.43)	2.42×10^{-9}	0.09 (0.04-0.19)
	Additive	A	329 (78.3%)	260 (71.0%)	165 (41.2%)	Ref	-	Ref	-	Ref	-
		G	91 (21.7%)	106 (29.0%)	235 (58.8%)	0.005	1.70 (1.18-2.45)	5.12×10^{-12}	6.04 (3.62-10.06)	6.81×10^{-12}	0.27 (0.18-0.39)
	Dominant	AA	132 (62.8%)	97 (53.0%)	32 (16.0%)	Ref	-	Ref	-	Ref	-
		AG+GG	78 (37.2%)	86 (47.0%)	168 (84.0%)	0.020	1.73 (1.09-2.74)	5.97×10^{-9}	12.81 (5.43-30.26)	6.13×10^{-10}	0.14 (0.08-0.26)
	Recessive	AA+AG	197 (93.8%)	163 (89.1%)	133 (66.5%)	Ref	-	Ref	-	Ref	-
		GG	13 (6.2%)	20 (10.9%)	67 (33.5%)	0.030	2.46 (1.09-5.54)	2.40×10^{-6}	9.95 (3.83-25.84)	1.47×10^{-5}	0.23 (0.12-0.45)
	Overdominant	AA+GG	145 (69.0%)	117 (63.9%)	99 (49.5%)	Ref	-	Ref	-	Ref	-
		AG	65 (31.0%)	66 (36.1%)	101 (50.5%)	0.259	1.31 (0.82-2.12)	0.006	2.53 (1.30-4.93)	0.011	0.51 (0.31-0.86)

SPSS 16.0 was used to obtain p values after adjusting with age and gender. Significance was maintained at ≤0.05 after FDR correction. *TERC,* telomerase RNA component; HAPE-f, HAPE-free; HAPE-p, HAPE-patients; HLs, Highlanders; n, number of samples; OR, odds ratio; CI, confidence interval; Ref, reference.

[B] Comparison of genotype and allele distribution in HLs with Han Chinese (CHB+CHS) population and Japanese (JPT) population.

TERC								
**SNP**	**Genotype/ Allele**	**CHB+CHS (Ref, n=204)**	**JPT (Ref, n=104)**	**HLs (n=200)**	**HLs vs CHB+CHS**		**HLs vs JPT**	
					**p value**	**OR (95%CI)**	**p value**	**OR (95%CI)**
rs2293607	AA	40 (19.2%)	9 (8.7%)	32 (16.0%)	Ref	-	Ref	-
	AG	105 (50.5%)	53 (51.0%)	101 (50.5%)	0.503	1.20 (0.70-2.06)	0.128	0.54 (0.24-1.21)
	GG	63 (30.3%)	42 (40.4%)	67 (33.5%)	0.334	1.33 (0.75-2.37)	0.056	0.45 (0.20-1.03)
	A	185 (44.5%)	71 (34.1%)	165 (41.3%)	Ref	-	Ref	-
	G	231 (55.5%)	137 (65.9%)	235 (58.8%)	0.353	1.14 (0.86-1.51)	0.088	0.74 (0.52-1.05)

SISA online tool was used to obtain p values. Significance was maintained at ≤0.05 after FDR correction. *TERC,* telomerase RNA component; CHB+CHS, Han Chinese population comprising of Han Chinese in Beijing (CHB) and Southern Han Chinese (CHS); JPT, Japanese population; HLs, highlanders; Ref, reference; n, number of samples; OR, odds ratio; CI, confidence interval.

Supplementary Table S11: Genotype and allele distribution of 9 SNPs of *TEP1*

[A] Comparison of genotype and allele distribution among HAPE-f, HAPE-p and HLs.

TEP1										
**SNP**	**Genetic Model**	**Genotype/ Allele**	**HAPE-f (n=210)**	**HAPE-p (n=183)**	**HLs (n=200)**	**HAPE-p vs HAPE-f**		**HLs vs HAPE-f**		**HAPE-p vs HLs**	
						**p value**	**OR (95%CI)**	**p value**	**OR (95%CI)**	**p value**	**OR (95%CI)**
rs2104978	Codominant	AA	182 (86.7%)	138 (75.4%)	192 (96.0%)	Ref	-	Ref	-	Ref	-
		AG	23 (11.0%)	39 (21.3%)	3 (1.5%)	0.045	1.90 (1.02-3.56)	0.003	0.05 (0.01-0.36)	1.83×10^{-5}	18.91 (4.93-72.56)
		GG	5 (2.3%)	6 (3.3%)	5 (2.5%)	0.628	0.69 (0.15-3.14)	0.698	0.65 (0.07-5.77)	0.731	1.30 (0.29-5.71)
	Additive	A	387 (92.1%)	315 (86.1%)	387 (96.8%)	Ref	-	Ref	-	Ref	-
		G	33 (7.9%)	51 (13.9%)	13 (3.2%)	0.176	1.44 (0.85-2.42)	0.009	0.24 (0.08-0.70)	1.44×10^{-4}	4.31 (2.03-9.16)
	Dominant	AA	182 (86.7%)	138 (75.4%)	192 (96.0%)	Ref	-	Ref	-	Ref	-
		AG+GG	28 (13.3%)	45 (24.6%)	8 (4.0%)	0.081	1.68 (0.94-3.01)	0.005	0.15 (0.04-0.57)	2.66×10^{-5}	7.29 (2.88-18.40)
	Recessive	AA+AG	205 (97.6%)	177 (96.7%)	195 (97.5%)	Ref	-	Ref	-	Ref	-
		GG	5 (2.4%)	6 (3.3%)	5 (2.5%)	0.541	0.63 (0.14-2.81)	0.816	0.78 (0.09-6.43)	0.968	1.03 (0.24-4.50)
	Overdominant	AA+GG	187 (89.0%)	144 (78.7%)	197 (98.5%)	Ref	-	Ref	-	Ref	-
		AG	23 (11.0%)	39 (21.3%)	3 (1.5%)	0.037	1.93 (1.04-3.59)	0.003	0.06 (0.01-0.40)	1.93×10^{-5}	18.30 (4.82-69.42)
rs1713449	Codominant	CC	108 (51.4%)	109 (59.6%)	99 (49.5%)	Ref	-	Ref	-	Ref	-
		CT	78 (37.1%)	54 (29.5%)	69 (34.5%)	0.212	0.73 (0.44-1.20)	0.287	0.67 (0.33-1.39)	0.096	0.62 (0.35-1.09)
		TT	24 (11.5%)	20 (10.9%)	32 (16.0%)	0.442	0.74 (0.34-1.60)	0.997	1.00 (0.37-2.72)	0.176	0.60 (0.28-1.26)
	Additive	C	294 (70.0%)	272 (74.3%)	267 (66.8%)	Ref	-	Ref	-	Ref	-
		T	126 (30.0%)	94 (25.7%)	133 (33.2%)	0.211	0.80 (0.56-1.14)	0.667	0.90 (0.55-1.47)	0.045	0.67 (0.45-0.99)
	Dominant	CC	108 (51.4%)	109 (59.6%)	99 (49.5%)	Ref	-	Ref	-	Ref	-
		CT+TT	102 (48.6%)	74 (40.4%)	101 (50.5%)	0.186	0.74 (0.47-1.16)	0.417	0.76 (0.40-1.47)	0.056	0.61 (0.37-1.01)
	Recessive	CC+CT	186 (88.5%)	163 (89.1%)	168 (84.0%)	Ref	-	Ref	-	Ref	-
		TT	24 (11.5%)	20 (10.9%)	32 (16.0%)	0.662	0.85 (0.41-1.75)	0.723	1.18 (0.46-3.02)	0.323	0.69 (0.33-1.44)
	Overdominant	CC+TT	132 (62.9%)	129 (70.5%)	131 (65.5%)	Ref	-	Ref	-	Ref	-
		CT	78 (37.1%)	54 (29.5%)	69 (34.5%)	0.271	0.76 (0.47-1.23)	0.274	0.68 (0.34-1.35)	0.186	0.69 (0.40-1.19)

SNP	Genetic Model	Genotype/ Allele	HAPE-f (n=210)	HAPE-p (n=183)	HLs (n=200)	HAPE-p vs HAPE-f		HLs vs HAPE-f		HAPE-p vs HLs	
						p value	OR (95%CI)	p value	OR (95%CI)	p value	OR (95%CI)
rs2228041	Codominant	GG	181 (86.2%)	145 (79.2%)	192 (96.0%)	Ref	-	Ref	-	Ref	-
		GA	25 (11.9%)	33 (18.0%)	6 (3.0%)	0.319	1.38 (0.73-2.60)	0.002	0.08 (0.02-0.39)	0.001	6.41 (2.24-18.32)
		AA	4 (1.9%)	5 (2.7%)	2 (1.0%)	0.326	2.09 (0.48-9.17)	0.198	3.78 (0.50-28.66)	0.744	0.75 (0.14-4.12)
	Additive	G	387 (92.1%)	323 (88.3%)	390 (97.5%)	Ref	-	Ref	-	Ref	-
		A	33 (7.9%)	43 (11.7%)	10 (2.5%)	0.150	1.48 (0.87-2.51)	0.096	0.40 (0.13-1.18)	0.008	3.06 (1.34-6.98)
	Dominant	GG	181 (86.2%)	145 (79.2%)	192 (96.0%)	Ref	-	Ref	-	Ref	-
		GA+AA	29 (13.8%)	38 (20.8%)	8 (4.0%)	0.213	1.46 (0.81-2.64)	0.017	0.21 (0.06-0.76)	0.002	4.39 (1.73-11.12)
	Recessive	GG+GA	206 (98.1%)	178 (97.3%)	198 (99.0%)	Ref	-	Ref	-	Ref	-
		AA	4 (1.9%)	5 (2.7%)	2 (1.0%)	0.376	1.92 (0.45-8.16)	0.175	3.93 (0.54-28.37)	0.588	0.63 (0.11-3.41)
	Overdominant	GG+AA	185 (88.1%)	150 (82.0%)	194 (97.0%)	Ref	-	Ref	-	Ref	-
		GA	25 (11.9%)	33 (18.0%)	6 (3.0%)	0.347	1.35 (0.72-2.54)	0.002	0.09 (0.02-0.40)	4.81×10^{-4}	6.46 (2.27-18.40)
rs2228036	Codominant	GG	134 (63.8%)	95 (52.0%)	127 (63.5%)	Ref	-	Ref	-	Ref	-
		GT	69 (32.9%)	72 (39.3%)	57 (28.5%)	0.581	1.15 (0.70-1.87)	0.267	0.67 (0.32-1.37)	0.040	1.77 (1.03-3.04)
		TT	7 (3.3%)	16 (8.7%)	16 (8.0%)	0.042	2.84 (1.04-7.77)	0.806	1.21 (0.26-5.65)	0.406	1.48 (0.59-3.73)
	Additive	G	337 (80.2%)	262 (71.6%)	311 (77.8%)	Ref	-	Ref	-	Ref	-
		T	83 (19.8%)	104 (28.4%)	89 (22.2%)	0.087	1.38 (0.95-2.01)	0.447	0.80 (0.45-1.42)	0.053	1.50 (0.99-2.27)
	Dominant	GG	134 (63.8%)	95 (52.0%)	127 (63.5%)	Ref	-	Ref	-	Ref	-
		GT+TT	76 (36.2%)	88 (48.0%)	73 (36.5%)	0.266	1.30 (0.82-2.06)	0.321	0.71 (0.36-1.40)	0.034	1.74 (1.04-2.92)
	Recessive	GG+GT	203 (96.7%)	167 (91.3%)	184 (92.0%)	Ref	-	Ref	-	Ref	-
		TT	7 (3.3%)	16 (8.7%)	16 (8.0%)	0.047	2.73 (1.01-7.34)	0.831	1.19 (0.24-5.86)	0.600	1.28 (0.51-3.20)
	Overdominant	GG+TT	141 (67.1%)	111 (60.7%)	143 (71.5%)	Ref	-	Ref	-	Ref	-
		GT	69 (32.9%)	72 (39.3%)	57 (28.5%)	0.916	1.03 (0.64-1.65)	0.268	0.67 (0.33-1.36)	0.057	1.68 (0.98-2.87)

SNP	Genetic Model	Genotype/ Allele	HAPE-f (n=210)	HAPE-p (n=183)	HLs (n=200)	HAPE-p vs HAPE-f		HLs vs HAPE-f		HAPE-p vs HLs	
						p value	OR (95%CI)	p value	OR (95%CI)	p value	OR (95%CI)
rs1760898	Codominant	CC	85 (40.5%)	81 (44.3%)	90 (45.0%)	Ref	-	Ref	-	Ref	-
		CA	88 (41.9%)	77 (42.0%)	88 (44.0%)	0.859	0.96 (0.58-1.58)	0.336	0.68 (0.32-1.48)	0.617	1.15 (0.66-1.99)
		AA	37 (17.6%)	25 (13.7%)	22 (11.0%)	0.176	0.63 (0.33-1.23)	0.297	0.61 (0.24-1.55)	0.920	1.04 (0.48-2.26)
	Additive	C	258 (61.4%)	239 (65.3%)	268 (67.0%)	Ref	-	Ref	-	Ref	-
		A	162 (38.6%)	127 (34.7%)	132 (33.0%)	0.201	0.81 (0.58-1.12)	0.196	0.73 (0.45-1.18)	0.819	1.04 (0.72-1.52)
	Dominant	CC	85 (40.5%)	81 (44.3%)	90 (45.0%)	Ref	-	Ref	-	Ref	-
		CA+AA	125 (59.5%)	102 (55.7%)	110 (55.0%)	0.473	0.85 (0.54-1.33)	0.237	0.67 (0.35-1.30)	0.696	1.11 (0.67-1.83)
	Recessive	CC+CA	173 (82.4%)	158 (86.3%)	178 (89.0%)	Ref	-	Ref	-	Ref	-
		AA	37 (17.6%)	25 (13.7%)	22 (11.0%)	0.161	0.64 (0.34-1.20)	0.434	0.70 (0.28-1.72)	0.899	0.95 (0.44-2.06)
	Overdominant	CC+AA	122 (58.1%)	106 (58.0%)	112 (56.0%)	Ref	-	Ref	-	Ref	-
		CA	88 (41.9%)	77 (42.0%)	88 (44.0%)	0.741	1.08 (0.68-1.71)	0.537	0.81 (0.41-1.60)	0.634	1.13 (0.68-1.88)
rs1760897	Codominant	TT	92 (43.8%)	70 (38.3%)	73 (36.5%)	Ref	-	Ref	-	Ref	-
		TC	81 (38.6%)	80 (43.7%)	90 (45.0%)	0.481	1.20 (0.73-1.97)	0.640	1.18 (0.58-2.41)	0.991	1.00 (0.58-1.75)
		CC	37 (17.6%)	33 (18.0%)	37 (18.5%)	0.399	1.31 (0.70-2.47)	0.508	1.36 (0.55-3.35)	0.627	0.85 (0.43-1.67)
	Additive	T	265 (63.1%)	220 (60.1%)	236 (59.0%)	Ref	-	Ref	-	Ref	-
		C	155 (36.9%)	146 (39.9%)	164 (41.0%)	0.366	1.16 (0.84-1.62)	0.455	1.20 (0.75-1.91)	0.602	0.91 (0.63-1.30)
	Dominant	TT	92 (43.8%)	70 (38.3%)	73 (36.5%)	Ref	-	Ref	-	Ref	-
		TC+CC	118 (56.2%)	113 (61.7%)	127 (63.5%)	0.410	1.21 (0.76-1.93)	0.503	1.26 (0.65-2.44)	0.822	0.94 (0.56-1.58)
	Recessive	TT+TC	173 (82.4%)	150 (82.0%)	163 (81.5%)	Ref	-	Ref	-	Ref	-
		CC	37 (17.6%)	33 (18.0%)	37 (18.5%)	0.564	1.19 (0.66-2.15)	0.632	1.22 (0.53-2.81)	0.515	0.81 (0.43-1.53)
	Overdominant	TT+CC	129 (61.4%)	103 (56.3%)	110 (55.0%)	Ref	-	Ref	-	Ref	-
		TC	81 (38.6%)	80 (43.7%)	90 (45.0%)	0.708	1.09 (0.69-1.73)	0.769	1.10 (0.57-2.15)	0.769	1.08 (0.65-1.79)

SNP	Genetic Model	Genotype/ Allele	HAPE-f (n=210)	HAPE-p (n=183)	HLs (n=200)	HAPE-p vs HAPE-f		HLs vs HAPE-f		HAPE-p vs HLs	
						p value	OR (95%CI)	p value	OR (95%CI)	p value	OR (95%CI)
rs2184282	Codominant	AA	138 (65.7%)	134 (73.2%)	97 (48.5%)	Ref	-	Ref	-	Ref	-
		AG	58 (27.6%)	41 (22.4%)	69 (34.5%)	0.108	0.64 (0.37-1.10)	0.015	2.43 (1.18-4.97)	0.002	0.39 (0.22-0.70)
		GG	14 (6.7%)	8 (4.4%)	34 (17.0%)	0.550	0.74 (0.27-2.01)	0.004	4.60 (1.63-12.95)	2.38×10^{-5}	0.11 (0.04-0.31)
	Additive	A	334 (79.5%)	309 (84.4%)	263 (65.8%)	Ref	-	Ref	-	Ref	-
		G	86 (20.5%)	57 (15.6%)	137 (34.2%)	0.128	0.72 (0.47-1.10)	2.95×10^{-4}	2.52 (1.53-4.16)	7.81×10^{-8}	0.31 (0.20-0.47)
	Dominant	AA	138 (65.7%)	134 (73.2%)	97 (48.5%)	Ref	-	Ref	-	Ref	-
		AG+GG	72 (34.3%)	49 (26.8%)	103 (51.5%)	0.105	0.66 (0.40-1.09)	0.002	2.92 (1.49-5.73)	1.10×10^{-5}	0.30 (0.17-0.51)
	Recessive	AA+AG	196 (93.3%)	175 (95.6%)	166 (83.0%)	Ref	-	Ref	-	Ref	-
		GG	14 (6.7%)	8 (4.4%)	34 (17.0%)	0.723	0.84 (0.31-2.23)	0.021	3.12 (1.19-8.19)	3.29×10^{-4}	0.18 (0.07-0.46)
	Overdominant	AA+GG	152 (72.4%)	142 (77.6%)	131 (65.5%)	Ref	-	Ref	-	Ref	-
		AG	58 (27.6%)	41 (22.4%)	69 (34.5%)	0.124	0.66 (0.39-1.12)	0.086	1.82 (0.92-3.61)	0.023	0.52 (0.30-0.91)
rs4246977	Codominant	TT	57 (27.1%)	45 (24.6%)	127 (63.5%)	Ref	-	Ref	-	Ref	-
		TC	105 (50.0%)	99 (54.1%)	60 (30.0%)	0.493	1.21 (0.70-2.11)	2.88×10^{-4}	0.22 (0.10-0.50)	8.99×10^{-7}	4.20 (2.37-7.44)
		CC	48 (22.9%)	39 (21.3%)	13 (6.5%)	0.525	0.81 (0.42-1.56)	4.58×10^{-5}	0.09 (0.03-0.28)	8.50×10^{-6}	6.76 (2.92-15.69)
	Additive	T	219 (52.1%)	189 (51.6%)	314 (78.5%)	Ref	-	Ref	-	Ref	-
		C	201 (47.9%)	177 (48.4%)	86 (21.5%)	0.538	0.90 (0.66-1.25)	2.68×10^{-7}	0.25 (0.15-0.42)	1.35×10^{-8}	3.08 (2.09-4.54)
	Dominant	TT	57 (27.1%)	45 (24.6%)	127 (63.5%)	Ref	-	Ref	-	Ref	-
		TC+CC	153 (72.9%)	138 (75.4%)	73 (36.5%)	0.807	1.07 (0.64-1.79)	4.59×10^{-6}	0.19 (0.09-0.39)	1.58×10^{-8}	4.76 (2.77-8.19)
	Recessive	TT+TC	162 (77.1%)	144 (78.7%)	187 (93.5%)	Ref	-	Ref	-	Ref	-
		CC	48 (22.9%)	39 (21.3%)	13 (6.5%)	0.189	0.69 (0.39-1.20)	0.001	0.15 (0.05-0.45)	0.003	3.36 (1.50-7.51)
	Overdominant	TT+CC	105 (50.0%)	84 (45.9%)	140 (70.0%)	Ref	-	Ref	-	Ref	-
		TC	105 (50.0%)	99 (54.1%)	60 (30.0%)	0.195	1.35 (0.86-2.12)	0.030	0.47 (0.24-0.93)	3.26×10^{-4}	2.62 (1.55-4.44)

SNP	Genetic Model	Genotype/ Allele	HAPE-f (n=210)	HAPE-p (n=183)	HLs (n=200)	HAPE-p vs HAPE-f		HLs vs HAPE-f		HAPE-p vs HLs	
						p value	OR (95%CI)	p value	OR (95%CI)	p value	OR (95%CI)
rs75932631	Codominant	TT	162 (77.1%)	146 (79.8%)	113 (56.5%)	Ref	-	Ref	-	Ref	-
		TC	42 (20.0%)	33 (18.0%)	70 (35.0%)	0.806	0.93 (0.51-1.68)	1.66×10^{-4}	4.47 (2.05-9.73)	2.42×10^{-4}	0.33 (0.18-0.59)
		CC	6 (2.9%)	4 (2.2%)	17 (8.5%)	0.799	0.83 (0.19-3.55)	0.012	5.86 (1.48-23.16)	0.001	0.09 (0.02-0.36)
	Additive	T	366 (87.1%)	325 (88.8%)	296 (74.0%)	Ref	-	Ref	-	Ref	-
		C	54 (12.9%)	41 (11.2%)	104 (26.0%)	0.720	0.91 (0.56-1.50)	1.42×10^{-5}	3.52 (1.99-6.22)	9.00×10^{-7}	0.30 (0.18-0.48)
	Dominant	TT	162 (77.1%)	146 (79.8%)	113 (56.5%)	Ref	-	Ref	-	Ref	-
		TC+CC	48 (22.9%)	37 (20.2%)	87 (43.5%)	0.747	0.91 (0.52-1.59)	3.61×10^{-5}	4.57 (2.22-9.39)	1.09×10^{-5}	0.28 (0.16-0.49)
	Recessive	TT+TC	204 (97.1%)	179 (97.8%)	183 (91.5%)	Ref	-	Ref	-	Ref	-
		CC	6 (2.9%)	4 (2.2%)	17 (8.5%)	0.838	0.86 (0.20-3.67)	0.051	3.86 (0.99-14.98)	0.004	0.15 (0.04-0.56)
	Overdominant	TT+CC	168 (80.0%)	150 (82.0%)	130 (65.0%)	Ref	-	Ref	-	Ref	-
		TC	42 (20.0%)	33 (18.0%)	70 (35.0%)	0.798	0.93 (0.52-1.66)	0.001	3.57 (1.72-7.42)	0.001	0.38 (0.21-0.68)

SPSS 16.0 was used to obtain p values after adjusting with age and gender. Significance was maintained at ≤0.05 after FDR correction. *TEP1,* telomerase associated protein 1; HAPE-f, HAPE-free; HAPE-p, HAPE-patients; HLs, Highlanders; n, number of samples; OR, odds ratio; CI, confidence interval; Ref, reference.

[B] Comparison of genotype and allele distribution in HLs with Han Chinese (CHB+CHS) population and Japanese (JPT) population.

TEP1								
**SNP**	**Genotype/ Allele**	**CHB+CHS (Ref, n=204)**	**JPT (Ref, n=104)**	**HLs (n=200)**	**HLs vs CHB+CHS**		**HLs vs JPT**	
					**p value**	**OR (95%CI)**	**p value**	**OR (95%CI)**
rs2104978	AA	208 (100%)	104 (100%)	192 (96.0%)	Ref	-	Ref	-
	AG	0 (0%)	0 (0%)	3 (1.5%)	*	-	*	-
	GG	0 (0%)	0 (0%)	5 (2.5%)	*	-	*	-
	A	416 (100.0%)	208 (100.0%)	387 (96.8%)	Ref	-	Ref	-
	G	0 (0.0%)	0 (0.0%)	13 (3.3%)	*	-	*	-
rs1713449	CC	86 (41.3%)	61 (58.7%)	99 (49.5%)	Ref	-	Ref	-
	CT	102 (49.0%)	37 (35.6%)	69 (34.5%)	0.013	0.59 (0.39-0.90)	0.594	1.15 (0.69-1.92)
	TT	20 (9.6%)	6 (5.8%)	32 (16.0%)	0.304	1.39 (0.74-2.61)	0.009	3.29 (1.30-8.32)
	C	274 (65.9%)	159 (76.4%)	267 (66.8%)	Ref	-	Ref	-
	T	142 (34.1%)	49 (23.6%)	133 (33.3%)	0.789	0.96 (0.72-1.29)	0.013	1.62 (1.10-2.37)
rs2228041	GG	208 (100%)	104 (100%)	192 (96.0%)	Ref	-	Ref	-
	GA	0 (0%)	0 (0%)	6 (3.0%)	*	-	*	-
	AA	0 (0%)	0 (0%)	2 (1.0%)	*	-	*	-
	G	416 (100.0%)	208 (100.0%)	390 (97.5%)	Ref	-	Ref	-
	A	0 (0.0%)	0 (0.0%)	10 (2.5%)	*	-	*	-
rs2228036	GG	153 (73.6%)	69 (66.3%)	127 (63.5%)	Ref	-	Ref	-
	GT	55 (26.4%)	32 (30.8%)	57 (28.5%)	0.321	1.25 (0.81-1.94)	1.000	0.97 (0.57-1.63)
	TT	0 (0.0%)	3 (2.9%)	16 (8.0%)	*	-	0.125	2.90 (0.82-10.29)
	G	361 (86.8%)	170 (81.7%)	311 (77.8%)	Ref	-	Ref	-
	T	55 (13.2%)	38 (18.3%)	89 (22.3%)	0.001	1.88 (1.30-2.72)	0.252	1.28 (0.84-1.96)

SNP	Genotype/ Allele	CHB+CHS (Ref, n=204)	JPT (Ref, n=104)	HLs (n=200)	HLs vs CHB+CHS		HLs vs JPT	
					p value	OR (95%CI)	p value	OR (95%CI)
rs1760898	CC	70 (33.7%)	49 (47.1%)	90 (45.0%)	Ref	-	Ref	-
	CA	101 (48.6%)	43 (41.3%)	88 (44.0%)	0.071	0.68 (0.44-1.04)	0.674	1.11 (0.67-1.84)
	AA	37 (17.8%)	12 (11.5%)	22 (11.0%)	0.013	0.46 (0.25-0.85)	1.000	1.00 (0.46-2.19)
	C	241 (57.9%)	141 (67.8%)	268 (67.0%)	Ref	-	Ref	-
	A	175 (42.1%)	67 (32.2%)	132 (33.0%)	0.008	0.68 (0.51-0.90)	0.844	1.04 (0.73-1.48)
rs1760897	TT	129 (62.0%)	71 (68.3%)	73 (36.5%)	Ref	-	Ref	-
	TC	69 (33.2%)	31 (29.8%)	90 (45.0%)	1.05×10^{-4}	2.30 (1.51-3.53)	7.90×10^{-5}	2.82 (1.67-4.76)
	CC	10 (4.8%)	2 (1.9%)	37 (18.5%)	<0.000001	6.54 (3.07-13.91)	1.00×10^{-6}	17.99 (4.18-77.47)
	T	327 (78.6%)	173 (83.2%)	236 (59.0%)	Ref	-	Ref	-
	C	89 (21.4%)	35 (16.8%)	164 (41.0%)	<0.000001	2.55 (1.88-3.47)	<0.000001	3.43 (2.27-5.20)
rs2184282	AA	120 (57.7%)	65 (62.5%)	97 (48.5%)	Ref	-	Ref	-
	AG	76 (36.5%)	33 (31.7%)	69 (34.5%)	0.589	0.89 (0.58-1.36)	0.203	1.40 (0.83-2.36)
	GG	12 (5.8%)	6 (5.8%)	34 (17.0%)	3.19×10^{-4}	3.51 (1.72-7.13)	0.003	3.80 (1.51-9.56)
	A	316 (76.0%)	163 (78.4%)	263 (65.8%)	Ref	-	Ref	-
	G	100 (24.0%)	45 (21.6%)	137 (34.3%)	0.001	1.65 (1.21-2.23)	0.001	1.89 (1.28-2.79)
rs4246977	TT	90 (43.3%)	51 (49.0%)	127 (63.5%)	Ref	-	Ref	-
	TC	98 (47.1%)	45 (43.3%)	60 (30.0%)	8.50×10^{-5}	0.43 (0.29-0.66)	0.015	0.54 (0.32-0.89)
	CC	20 (9.6%)	8 (7.7%)	13 (6.5%)	0.039	0.46 (0.22-0.97)	0.370	0.65 (0.26-1.67)
	T	278 (66.8%)	147 (70.7%)	314 (78.5%)	Ref	-	Ref	-
	C	138 (33.2%)	61 (29.3%)	86 (21.5%)	1.88×10^{-4}	0.55 (0.40-0.76)	0.032	0.66 (0.45-0.97)

SNP	Genotype/ Allele	CHB+CHS (Ref, n=204)	JPT (Ref, n=104)	HLs (n=200)	HLs vs CHB+CHS		HLs vs JPT	
					p value	OR (95%CI)	p value	OR (95%CI)
rs75932631	TT	131 (63.0%)	67 (64.4%)	113 (56.5%)	Ref	-	Ref	-
	TC	68 (32.7%)	33 (31.7%)	70 (35.0%)	0.407	1.19 (0.79-1.81)	0.380	1.26 (0.75-2.10)
	CC	9 (4.3%)	4 (3.8%)	17 (8.5%)	0.064	2.19 (0.94-5.10)	0.146	2.52 (0.81-7.80)
	T	330 (79.3%)	167 (80.3%)	296 (74.0%)	Ref	-	Ref	-
	C	86 (20.7%)	41 (19.7%)	104 (26.0%)	0.072	1.35 (0.97-1.87)	0.084	1.43 (0.95-2.15)

SISA online tool was used to obtain p values. Significance was maintained at ≤0.05 after FDR correction. *Analysis could not be performed because the representatives of an allele were absent in a study group. *TEP1,* telomerase associated protein 1; CHB+CHS, Han Chinese population comprising of Han Chinese in Beijing (CHB) and Southern Han Chinese (CHS); JPT, Japanese population; HLs, highlanders; Ref, reference; n, number of samples; OR, odds ratio; CI, confidence interval.

Supplementary Table S12: Genotype and allele distribution of *HSP90AA1* SNP

[A] Comparison of genotype and allele distribution among HAPE-f, HAPE-p and HLs.

HSP90AA1											
**SNP**	**Genetic Model**	**Genotype / Allele**	**HAPE-f (n=210)**	**HAPE-p (n=183)**	**HLs (n=200)**	**HAPE-p vs HAPE-f**		**HLs vs HAPE-f**		**HAPE-p vs HLs**	
						**p value**	**OR (95%CI)**	**p value**	**OR (95%CI)**	**p value**	**OR (95%CI)**
rs8005905	Codominant	AA	166 (79.0%)	143 (78.1%)	137 (68.5%)	Ref	-	Ref	-	Ref	-
		AT	38 (18.1%)	36 (19.7%)	61 (30.5%)	0.976	1.01 (0.56-1.82)	0.681	0.84 (0.36-1.95)	0.374	0.77 (0.43-1.38)
		TT	6 (2.9%)	4 (2.2%)	2 (1.0%)	0.988	1.01 (0.23-4.37)	0.001	0.01 (0.001-0.16)	0.553	1.93 (0.22-16.96)
	Additive	A	370 (88.1%)	322 (88.0%)	335 (83.8%)	Ref	-	Ref	-	Ref	-
		T	50 (11.9%)	44 (12.0%)	65 (16.2%)	0.993	1.00 (0.61-1.65)	0.198	0.61 (0.29-1.29)	0.625	0.88 (0.52-1.48)
	Dominant	AA	166 (79.0%)	143 (78.1%)	137 (68.5%)	Ref	-	Ref	-	Ref	-
		AT+TT	44 (21.0%)	40 (21.9%)	63 (31.5%)	0.992	1.00 (0.58-1.75)	0.413	0.71 (0.32-1.61)	0.472	0.81 (0.45-1.44)
	Recessive	AA+AT	204 (97.1%)	179 (97.8%)	198 (99.0%)	Ref	-	Ref	-	Ref	-
		TT	6 (2.9%)	4 (2.2%)	2 (1.0%)	0.999	1.00 (0.23-4.27)	0.001	0.01 (0.001-0.15)	0.518	2.14 (0.21-21.35)
	Overdominant	AA+TT	172 (81.9%)	147 (80.3%)	139 (69.5%)	Ref	-	Ref	-	Ref	-
		AT	38 (18.1%)	36 (19.7%)	61 (30.5%)	0.991	1.00 (0.56-1.80)	0.817	0.91 (0.40-2.06)	0.362	0.76 (0.42-1.37)

SPSS 16.0 was used to obtain p values after adjusting with age and gender. Significance was maintained at ≤0.05 after FDR correction. *HSP90AA1,* heat shock protein 90; HAPE-f, HAPE-free; HAPE-p, HAPE-patients; HLs, Highlanders; n, number of samples; OR, odds ratio; CI, confidence interval; Ref, reference.

[B] Comparison of genotype and allele distribution in HLs with Han Chinese (CHB+CHS) population and Japanese (JPT) population.

HSP90AA1								
**SNP**	**Genotype/ Allele**	**CHB+CHS (Ref, n=204)**	**JPT (Ref, n=104)**	**HLs (n=200)**	**HLs vs CHB+CHS**		**HLs vs JPT**	
					**p value**	**OR (95%CI)**	**p value**	**OR (95%CI)**
rs8005905	AA	134 (64.4%)	66 (63.5%)	137 (68.5%)	Ref	-	Ref	-
	AT	64 (30.8%)	35 (33.7%)	61 (30.5%)	0.746	0.93 (0.61-1.42)	0.501	0.84 (0.51-1.40)
	TT	10 (4.8%)	3 (2.9%)	2 (1.0%)	0.035	0.20 (0.04-0.91)	0.335	0.32 (0.05-1.97)
	A	332 (79.8%)	167 (80.3%)	335 (83.8%)	Ref	-	Ref	-
	T	84 (20.2%)	41 (19.7%)	65 (16.3%)	0.145	0.77 (0.54-1.10)	0.286	0.79 (0.51-1.22)

SISA online tool was used to obtain p values. Significance was maintained at ≤0.05 after FDR correction. *HSP90AA1,* heat shock protein 90; CHB+CHS, Han Chinese population comprising of Han Chinese in Beijing (CHB) and Southern Han Chinese (CHS); JPT, Japanese population; HLs, highlanders; Ref, reference; n, number of samples; OR, odds ratio; CI, confidence interval.

Supplementary Table S13: Genotype and allele distribution and comparison of *PTGES3* SNPs in HAPE-f, HAPE-p and HLs

PTGES3												
**SNP**	**Genetic Model**	**Genotype / Allele**	**HAPE-f (n=210)**	**HAPE-p (n=183)**	**HLs (n=200)**	**HAPE-p vs HAPE-f**		**HLs vs HAPE-f**		**HAPE-p vs HLs**		
						**p value**	**OR (95%CI)**	**p value**	**OR (95%CI)**	**p value**	**OR (95%CI)**
rs191628793	Codominant	AA	197 (93.8%)	165 (90.2%)	190 (95.0%)	Ref	-	Ref	-	Ref	-
		AC	11 (5.2%)	11 (6.0%)	5 (2.5%)	0.971	0.98 (0.35-2.75)	0.009	0.08 (0.01-0.54)	0.157	2.64 (0.69-10.12)
		CC	2 (1.0%)	7 (3.8%)	5 (2.5%)	0.322	2.42 (0.42-13.98)	0.165	4.57 (0.53-38.97)	0.740	1.27 (0.31-5.29)
	Additive	A	405 (96.4%)	341 (93.2%)	385 (96.3%)	Ref	-	Ref	-	Ref	-
		C	15 (3.6%)	25 (6.8%)	15 (3.8%)	0.324	1.47 (0.69-3.13)	0.974	0.98 (0.28-3.37)	0.232	1.64 (0.73-3.70)
	Dominant	AA	197 (93.8%)	165 (90.2%)	190 (95.0%)	Ref	-	Ref	-	Ref	-
		AC+CC	13 (6.2%)	18 (9.8%)	10 (5.0%)	0.589	1.27 (0.53-3.03)	0.329	0.46 (0.09-2.21)	0.207	1.90 (0.70-5.15)
	Recessive	AA+AC	208 (99.0%)	176 (96.2%)	195 (97.5%)	Ref	-	Ref	-	Ref	-
		CC	2 (1.0%)	7 (3.8%)	5 (2.5%)	0.295	2.50 (0.45-13.98)	0.160	4.47 (0.55-36.20)	0.779	1.23 (0.30-5.08)
	Overdominant	AA+CC	199 (94.8%)	172 (94.0%)	195 (97.5%)	Ref	-	Ref	-	Ref	-
		AC	11 (5.2%)	11 (6.0%)	5 (2.5%)	0.939	0.96 (0.34-2.69)	0.009	0.08 (0.01-0.54)	0.158	2.63 (0.69-10.02)

SPSS 16.0 was used to obtain p values after adjusting with age and gender. Significance was maintained at ≤0.05 after FDR correction. *PTGES3,* Prostaglandin E Synthase 3; HAPE-f, HAPE-free; HAPE-p, HAPE-patients; HLs, Highlanders; n, number of samples; OR, odds ratio; CI, confidence interval; Ref, reference.

Note: The genotype data for P23 rs191628793A/C in CHB+CHS and JPT populations were unavailable.

Supplementary Table S14: Correlation analysis between biomarkers and clinical parameters in the three study groups

HAPE-f					
**Clinical parameters/ Biomarkers**	**Relative telomere length**	**Telomerase Activity**	**TERT**	**8-iso PGF2α**	**Total antioxidant activity**
**MAP**	r = –0.003<br>p = 0.97	r = 0.02<br>p = 0.81	r = –0.04<br>p = 0.71	r = 0.07<br>p = 0.35	r = 0.10<br>p = 0.37
**SaO_2**	r = –0.04<br>p = 0.59	r = 0.05<br>p = 0.59	r = –0.09<br>p = 0.46	r = –0.15<br>p = 0.05	r = 0.09<br>p = 0.44
**HAPE-p**					
**Clinical parameters/ Biomarkers**	**Relative telomere length**	**Telomerase Activity**	**TERT**	**8-iso PGF2α**	**Total antioxidant activity**
**MAP**	r = –0.05<br>p = 0.52	r = –0.06<br>p = 0.57	r = 0.21<br>p = 0.10	r = –0.03<br>p = 0.80	r = –0.22<br>p = 0.08
**SaO_2**	r = –0.02<br>p = 0.77	r = –0.23<br>p = 0.02	r = 0.14<br>p = 0.27	r = 0.07<br>p = 0.46	r = 0.19<br>p = 0.13
**HLs**					
**Clinical parameters/ Biomarkers**	**Relative telomere length**	**Telomerase Activity**	**TERT**	**8-iso PGF2α**	**Total antioxidant activity**
**MAP**	r = 0.09<br>p = 0.20	r = 0.09<br>p = 0.26	r = –0.07<br>p = 0.40	r = 0.05<br>p = 0.48	r = –0.25<br>p = 0.003
**SaO_2**	r = 0.08<br>p = 0.30	r = 0.10<br>p = 0.22	r = 0.13<br>p = 0.11	r = –0.08<br>p = 0.31	r = 0.33<br>p = 5.35×10^{-5}

SPSS 16.0 was used to obtain p values and the significance was maintained at $p \leq 0.05$ after Bonferroni's correction. P values were obtained after adjusting with age, gender, and BMI. HAPE-f, HAPE-free; HAPE-p, HAPE-patients; HLs, Highlanders; MAP, Mean arterial pressure; SaO_2, arterial oxygen saturation; TERT, Telomerase reverse transcriptase; 8-iso PGF2α. 8-isoprostaglandin F2α; r, correlation coefficient; p, p value.

Supplementary Table S15: Correlation analysis between the biomarkers in the three study groups

(A) HAPE-f

**Relative telomere length**	r = 1				
**Telomerase Activity**	r = -0.01<br>p = 0.92	r = 1			
**TERT**	r = –0.15<br>p = 0.24	r = 0.15<br>p = 0.23	r = 1		
**8-iso PGF2α**	r = 0.15<br>p = 0.05	r = 0.17<br>p = 0.06	r = –0.01<br>p = 0.96	r = 1	
**Total antioxidant activity**	r = –0.03<br>p = 0.77	r = –0.22<br>p = 0.09	r = –0.34<br>p = 0.002	r = 0.03<br>p = 0.80	r = 1
**Biomarkers**	**Relative telomere length**	**Telomerase Activity**	**TERT**	**8-iso PGF2α**	**Total antioxidant activity**

(B) HAPE-p

**Relative telomere length**	r = 1				
**Telomerase Activity**	r = 0.09<br>p = 0.35	r = 1			
**TERT**	r = –0.14<br>p = 0.27	r = 0.09<br>p = 0.46	r = 1		
**8-iso PGF2α**	r = –0.03<br>p = 0.80	r = 0.04<br>p = 0.77	r = –0.24<br>p = 0.06	r = 1	
**Total antioxidant activity**	r = –0.03<br>p = 0.81	r = 0.24<br>p = 0.06	r = 0.35<br>p = 0.004	r = 0.09<br>p = 0.49	r = 1
**Biomarkers**	**Relative telomere length**	**Telomerase Activity**	**TERT**	**8-iso PGF2α**	**Total antioxidant activity**

(C) HLs

**Relative telomere length**	r = 1				
**Telomerase Activity**	r = –0.05<br>p = 0.49	r = 1			
**TERT**	r = 0.02<br>p = 0.83	r = –0.15<br>p = 0.07	r = 1		
**8-iso PGF2α**	r = 0.03<br>p = 0.68	r = –0.05<br>p = 0.49	r = 0.02<br>p = 0.81	r = 1	
**Total antioxidant activity**	r = –0.05<br>p = 0.53	r = 0.03<br>p = 0.68	r = 0.10<br>p = 0.25	r = –0.23<br>p = 0.005	r = 1
**Biomarkers**	**Relative telomere length**	**Telomerase Activity**	**TERT**	**8-iso PGF2α**	**Total antioxidant activity**

SPSS 16.0 was used to obtain p values, and the significance was maintained at $p \leq 0.05$ after Bonferroni's correction. P values were obtained after adjusting with age, gender and BMI. HAPE-f, HAPE-free; HAPE-p, HAPE-patients; HLs, Highlanders; TERT, Telomerase reverse transcriptase; 8-iso PGF2α. 8-isoprostaglandin F2α; r, correlation coefficient; p, p-value.
